# Resolving a Nearly Two‐Centuries‐Old Mystery: On the Structural Chemistry and Physicochemical Properties of Compounds Associated With the Term Ammelide

**DOI:** 10.1002/chem.202503587

**Published:** 2025-12-31

**Authors:** Thaddäus J. Koller, Kristian Witthaut, Reinhard M. Pritzl, Simon M. J. Endraß, Markus Rösch, Georg Krach, Nadine Lammer, Thomas M. Klapötke, Wolfgang Schnick

**Affiliations:** ^1^ Department of Chemistry University of Munich (LMU) Munich Germany

**Keywords:** hydrogen bonds, self‐assembly, solid‐state structures, stacking interactions, tautomerism

## Abstract

Ammelide, also known as melanuric acid, is a simple molecular compound, which can be regarded as the double hydrolysis product of the industrially relevant compound melamine. Within this work, the first structural description of ammelide was provided almost 200 years after its discovery. This not only gave the first unambiguous evidence that ammelide's preferred tautomeric form in the solid is 6‐amino‐1,3,5‐triazine‐2,4(1*H*,3*H*)‐dione, but also showed that ammelide adopts a layered structure similar to the closely related ammeline. Moreover, the crystal structures of three modifications of the 1:1 adduct between ammeline and ammelide were elucidated, which form layers with a honeycomb motif and hexagonal voids analogous to melamine cyanurate. Differential thermal analysis and thermogravimetric analysis demonstrated that both pure ammelide and its adduct with ammeline have exceptionally high thermal stabilities, resulting from their dense hydrogen bonding networks. Finally, the synthesis and structural characterization of the ammelide's nitrate and perchlorate salts were carried out to examine the potential of ammelide as part of insensitive high‐energy‐density materials through sensitivity measurements and theoretical calculations.

## Graphical abstract

The crystal structure of ammelide, also known as melanuric acid, was solved almost 200 years after its discovery by Liebig. Moreover, three modifications of its 1:1 adduct with ammeline were structurally elucidated, consisting of layers with a honeycomb motif analogous to melamine cyanurate. Finally, ammelide's nitrate and perchlorate salts were prepared to examine the potential of ammelide as part of insensitive high‐energy‐density materials.

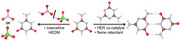

## 1 Introduction

**SCHEME 1:**
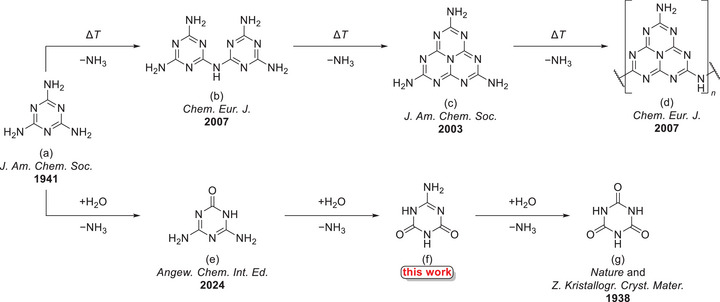
Molecular structures of melamine's (a) pyrolysis products melam (b), melem (c) and melon (d) as well as hydrolysis products ammeline (e), ammelide (f) and cyanuric acid (g) with the journal and year of their first structural description depicted below [4, 5, 6, 7, 8, 9, 10, 11, 12, 13, 14].

In 1834, the famous chemist Justus von Liebig described a series of nitrogen‐rich heterocyclic compounds [1]. Among these is melamine (2,4,6‐triamino‐1,3,5‐triazine), which finds nowadays industrial use in combination with formaldehyde as a resin material used for the preparation of thermosetting plastics [2, 3]. In addition, Liebig investigated several pyrolysis and hydrolysis products of melamine (Scheme 1).

**SCHEME 2:**
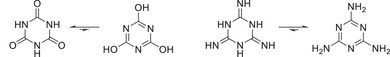
Illustration of the prevalent tautomeric form in the solid of cyanuric acid (left) and melamine (right) determined from their crystal structures.

A key feature of these compounds is the absence of any C─C and C─H bonds, resulting in high thermal stability and exceptional resistance to oxidation. Their second defining trait is their multitude of hydrogen bond acceptor (HBA) and donor (HBD) functionalities, which give them the ability to form dense hydrogen bonding networks, thereby further enhancing their thermal stabilities. Furthermore, these allow them to form self‐assembling monolayers (SAM) on surfaces of materials such as graphite and gold, which proved to be effective template structures for the adsorption of additional molecules [15, 16]. For SAM and other applications based on molecular self‐assembly, it is crucial to know a compound's crystal structure to fully understand the underlying hydrogen bonding network. In addition, as these compounds are potentially prone to tautomerization [17, 18, 19], crystal structures are necessary to determine the compounds’ prevalent tautomers in the solid (Scheme 2).

While melamine [4, 5] and cyanuric acid [6, 7, 8, 9] were among the first structurally elucidated molecular compounds by single crystal X‐ray diffraction (SCXRD), the crystal structure of melamine's pyrolysis products melam [10], melem [11, 12], and melon [13] have only been determined at the beginning of this century. Similarly, a structural description of ammeline has only been published in 2024 [14]. However, the first crystal structures of ammelinium (i.e., protonated ammeline) salts were already reported in 2006 [20]. Liu et al. expanded the scope of elucidated ammelinium salt structures and further showed that ammelinium, in combination with energetic counter anions such as perchlorate can be considered for insensitive high‐energy‐density materials (HEDMs) [21]. In stark contrast, a structural description of ammelide or any of its salts remained elusive to this date.

**FIGURE 1:**
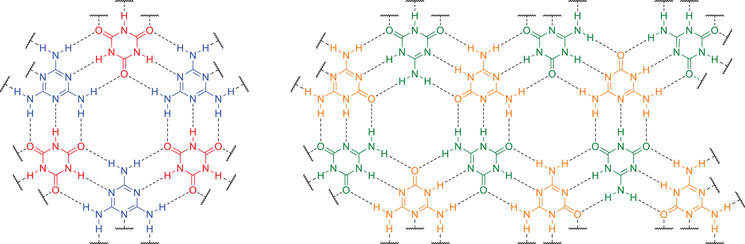
Illustration of the honeycomb arrangement within a layer of melamine cyanurate [27, 28] (left) compared to that conceivable for ammeline melanurate (right).

As already mentioned above, the term ammelide was first introduced by Liebig, who used it to name the material he obtained by hydrolysis of melam in concentrated H_2_SO_4_ at 180–190 °C [1]. However, it was later demonstrated that this material does not consist of a singular molecular species, but rather a mixture of ammeline and melanuric acid [22, 23, 24], which was first found by Wöhler and Liebig in the residue of urea distillations [25, 26]. As a result, the terms ammelide and melanuric acid have since been used to denote the same molecule [22, 23, 24]. For the material originally coined ammelide, Liebig determined an empirical formula of C_2_H_3_N_3_O [1], which is the same as that of melamine cyanurate. Melamine cyanurate is the trivial name for the 1:1 adduct between melamine and cyanuric acid. These two molecules can arrange into layers with hexagonal voids resembling a honeycomb [27, 28]. In addition to the aforementioned potential as a SAM template structure, these voids enable exfoliated melamine cyanurate nanosheets to embed Co^2+^‐based nanoparticles, preventing their aggregation, while at the same time boosting their catalytic hydrogen evolution reaction (HER) activity [29]. Similarly, SAM of melamine cyanurate were shown to enhance the catalytic HER activity when deposited on MoS_2_ [30]. Finally, melamine cyanurate is commonly employed as flame retardant for polyamide 6 and 66 due to its improved thermal stability compared to melamine and cyanuric acid [31]. Since ammeline and ammelide have complementary amounts of HBAs and HBDs, analogous to melamine and cyanuric acid, it is conceivable that these two compounds are also able to form a 1:1 adduct structurally closely related to melamine cyanurate. This adduct may be called ammeline melanurate by analogy with melamine cyanurate (Table 1 and Figure 1).

**TABLE 1:** Number of HBA and HBD functionalities as well as aqueous solubility at 20 °C (*S*_aq_) of melamine, ammeline, ammelide, cyanuric acid, melamine cyanurate and ammeline melanurate.

Name	HBA^a^	HBD	*S*_aq_ [32] [mg/l]
Melamine	3	6	3240
Ammeline	4	5	75
Ammelide	5	4	77
Cyanuric acid	6	3	2000
Melamine cyanurate^b^	4.5	4.5	2
Ammeline melanurate^b^	4.5	4.5	—^c^

^a^ Counting only lone pairs that are not involved in the π‐electron system.

^b^ Referring to the average per molecule.

^c^ Not yet determined.

The existence of such an adduct is supported by toxicokinetic studies on sheep, where the co‐exposure of ammeline and ammelide proved to be more toxic than either of the two compounds alone, as is also the case with melamine and cyanuric acid [33]. The reason for this is the much lower aqueous solubility of melamine cyanurate compared to its two components alone, which in turn results in the formation of melamine cyanurate crystals in the renal system, ultimately leading to kidney failure [32, 33]. It can therefore be assumed that Liebig's original ammelide synthesis did not result in the mere formation of an arbitrary mixture between ammeline and ammelide, but in the preparation of their 1:1 adduct.

The project's aim was to shed light on the structural chemistry of compounds associated with the term ammelide. For this purpose, straightforward methods were developed for the preparation of ammeline melanurate and its resolution into its two components. Outgoing of these materials, crystal growth strategies were developed to gain access to single crystals suitable for SCXRD, enabling the structure determination of ammelide itself as well as of three modifications of ammeline melanurate. Additionally, ammelidium (i.e., protonated ammelide) nitrate and perchlorate were prepared and analyzed on their potential as insensitive HEDM in comparison to their ammelinium analogues. Ammelide's higher oxygen content promised improved energetic performance values, while retaining the low sensitivity toward friction, impact and electrostatic discharge.

## 2 Results and Discussion

### 2.1 Preparation and Resolution of Ammeline Melanurate

**SCHEME 3:**
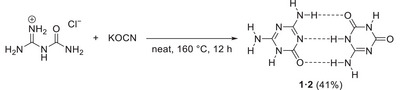
Synthetic conditions used for the preparation of ammeline melanurate (**1·2**) by reaction of guanylurea hydrochloride with KOCN.

Ammeline melanurate (**1·2**), the 1:1 adduct between ammeline (**1**) and ammelide (**2**), was prepared through solid‐state reaction of guanylurea hydrochloride with KOCN in a 1:1 molar ratio. This gave **1·2** in 41% yield after removal of the by‐product KCl through stirring in boiling H_2_O (Scheme 3). The employed guanylurea hydrochloride was prepared by the hydrolysis of dicyandiamide in aqueous HCl (2 m) (Figure S16), closely following a previously reported procedure [34].

Resolution of **1·2** was achieved by twofold recrystallization from boiling aqueous H_3_PO_4_ (0.2 m), whereby **2** was selectively precipitated, taking advantage of its lower basicity compared to **1** (p*K*_b_ 12.2 vs. 9.5) [35]. Neutralization of the filtrate of the first recrystallization from aqueous H_3_PO_4_ and subsequent twofold recrystallization of the resulting precipitate from boiling aqueous Na_2_CO_3_ (0.2 m) yielded **1** (Figure S20), exploiting its lower acidity compared to **2** (p*K*_a_ 9.4 vs. 6.9) [35].

### 2.2 Crystallization and Structural Analysis of Ammelide and Ammeline Melanurate

Since the analysis by powder X‐ray diffraction (PXRD) of the prepared **2** revealed its low crystallinity (Figure S21), an additional crystallization method was required for the elucidation of its crystal structure. The acquisition of suitable single crystals from an aqueous NH_3_ solution analogous to **1** [14] was not possible, because **2** forms a stable ammonium salt [36]. Therefore, attention was alternatively drawn to crystallization experiments under hydrothermal conditions in an autoclave, as these were also employed to prepare crystalline melamine cyanurate [27, 28]. Initially, an attempt was made to recrystallize **2** in pure H_2_O at 150 °C. However, this led to no significant improvement in crystallinity, probably because the solubility of **2** is still far too low at this temperature. Consequently, higher temperatures (≥ 200 °C) were examined, but these only resulted in the hydrolysis of **2** into cyanuric acid. Therefore, the solubility of **2** was alternatively increased by adjusting the pH value to around nine via the addition of K_2_HPO_4_. This yielded single crystals from which the crystal structure of **2** was elucidated by SCXRD. The obtained structure model was verified by Rietveld refinement of the PXRD pattern from the bulk material (Figure S22).

**FIGURE 2:**
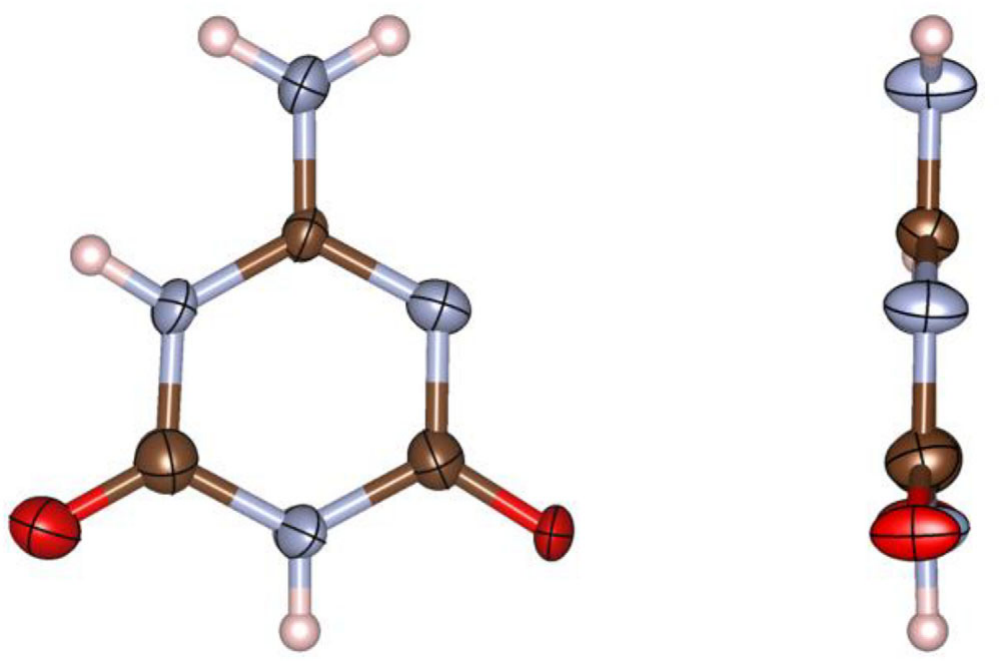
Asymmetric unit of ammelide (**2**) in the crystal viewed perpendicular (left) and parallel (right) to the triazine plane. All atoms besides the H atoms are drawn as thermal ellipsoids at the 80% probability level (atomic coloring: H white, C brown, N light‐blue, O red).

**2** crystallizes in space group *P*2_1_/*c* (no. 14; with *a* = 6.3345(12), *b* = 9.7509(13), *c* = 7.9355(14) Å; *β* = 98.535(9)° at −100 °C, Tables S3, S10, S11) [37] with four formula units per unit cell. In this structure, **2** occurs exclusively in its 6‐amino‐1,3,5‐triazine‐2,4(1*H*,3*H*)‐dione tautomeric form, as evidenced by the C─NH_2_ (1.327(5) Å) and C─O bond lengths (1.237(6) and 1.238(5) Å), indicating single and double bond character, respectively (Figure 2).

The bond angles within the triazine ring coincide with analogous angles in previously reported triazine structures. That means that the OC─N(H)─CO and HN─C(O)─NH bond angles of 124.0(4)° and 114.9(4)° are close to the C─N─C (Ø 125°) and N─C─N (Ø 115°) bond angles of cyanuric acid [38]. Likewise, the C─N─C and N─C─N bond angles of 122.0(4)° and 119.0(4)° at the other NH‐ and CO‐group, respectively, match well to those reported for **1** (120.79(11)° and 118.86(12)°) [14]. Finally, the remaining C─N─C (117.4(4)°) and N─C─N (122.6(4)°) bond angles are the only ones of their kind with a value below and above the regular hexagonal angle of 120°, respectively, analogous to the respective angles in melamine (Ø 114° and Ø 126°) [39].

**FIGURE 3:**
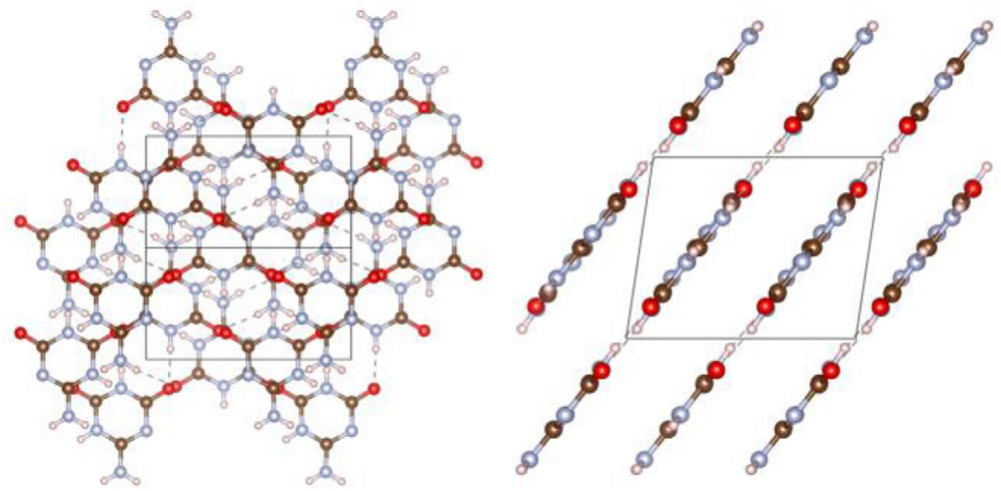
Crystal structure of ammelide (**2**) viewed along [101] (left) and [010] (right) (atomic coloring: H white, C brown, N light‐blue, O red).

The molecules of **2** arrange into layers parallel to (102), which are interconnected by staggered parallel π–π stacking. Within the layers, each molecule is surrounded by three others, to whom it is connected by a total of eight hydrogen bonds. This leads to a distorted honeycomb molecular arrangement as in **1** (Figure 3).

**FIGURE 4:**
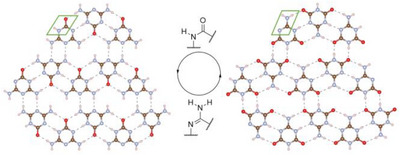
Section of one layer of ammeline (**1**) (left) and ammelide (**2**) (right) in comparison (atomic coloring: H white, C brown, N light‐blue, O red).

The strong similarity between the layers of **1** and **2** becomes clear when the two are shown side by side, revealing that one can be converted into the other when each OCNH moiety is replaced by an H_2_NCN one (Figure 4).

**FIGURE 5:**
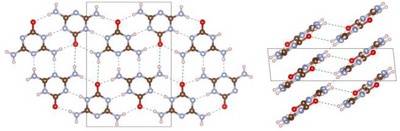
Crystal structure of ammeline (**1**) viewed along [100] (left) and [010] (right) with the attractive inter‐layer interactions highlighted in green (atomic coloring: H white, C brown, N light‐blue, O red).

In contrast to **1**, the layers of **2** are not slightly corrugated and instead perfectly planar. The corrugation in **1** causes the hydrogen atoms that are not part of the hydrogen bonding network within the layers to face more toward the oxygen atoms of adjacent layers. This implies attractive inter‐layer interactions between them via π‐electrons at the oxygen atoms (Figure 5), which also coincides with the fact that the stacking distance of **1** (3.012 Å) [14] is smaller than that of **2** (3.123 Å). Nevertheless, the density of **2** (1.755 g/cm^3^) is still marginally higher than of **1** (1.749 g/cm^3^), which can be attributed to the slightly shorter hydrogen bond acceptor A to donor D distances (i.e., A···H─D) within the layers of **2** (2.77–2.91 Å) compared to those found in **1** (2.86–2.96 Å) [14]. Additionally, the stacking pattern of the layers in **1** and **2** is different. While the layers of **1** exhibit an almost ecliptic stacking, the layers of **2** are strongly shifted against each other.

**FIGURE 6:**
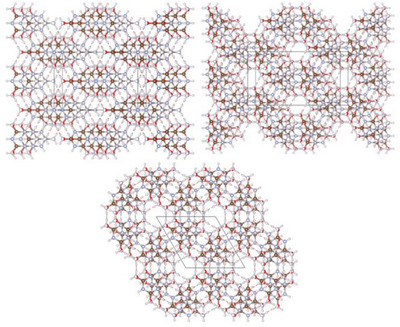
Crystal structure of α‐ammeline melanurate (**1·2α**) viewed along [10|10|] (top left), β‐ammeline melanurate (**1·2β**) viewed along [10|12|] (top right) and γ‐ammeline melanurate (**1·2γ**) viewed along [001] (bottom) (atomic coloring: H white, C brown, N light‐blue, O red).

**FIGURE 7:**
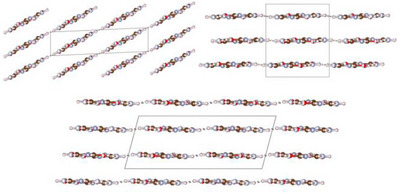
Crystal structure of α‐ammeline melanurate (**1·2α**) viewed along [010] (top left), β‐ammeline melanurate (**1·2β**) viewed along [010] (bottom) and γ‐ammeline melanurate (**1·2γ**) viewed along [100] (top right) (atomic coloring: H white, C brown, N light‐blue, O red).

After the successful structural elucidation of **2**, the focus was placed on the preparation of single crystals of **1·2** under hydrothermal conditions. As starting materials, either amorphous **1·2** or a 1:1 molar mixture of **1** and **2** were used. As for **2**, crystallization experiments in pure H_2_O did not provide sufficient samples for analysis via SCXRD. But adding K_2_HPO_4_ likewise made it possible to obtain a crystalline sample of **1·2** (Figure S17), consisting of a mixture of three distinct crystallographic modifications (Figure S18): α‐ammeline melanurate (**1·2α**) crystallizing in space group *C*2/*m* (no. 12; with *a* = 14.726(4), *b* = 9.570(2), *c* = 3.5570(9) Å; *β* = 92.571(9)° at −100 °C; Tables S2, S4, S5) [24] with two formula units per unit cell, β‐ammeline melanurate (**1·2β**) crystallizing in space group *C*2/*c* (no. 15; with *a* = 16.5876(14), *b* = 9.5937(8), *c* = 6.5959(6) Å; *β* = 104.985(3)° at −100 °C; Tables S2, S6, S7) [24] with four formula units per unit cell and γ‐ammeline melanurate (**1·2γ**) crystallizing in space group *P*3_1_21 (no. 152; with *a* = *b* = 9.5855(6), *c* = 9.4906(4) Å at −100 °C; Tables S2, S8, S9) [37] with three formula units per unit cell. In all three modifications, the molecules arrange into layers with a honeycomb motif interconnected by hydrogen bonding analogous to melamine cyanurate. As with **1**, adjacent layers are offset from each other by approximately one aromatic C─N bond length to achieve electrostatic attractive π−π stacking, since this positions partially negatively charged nitrogen/oxygen atoms above partially positively charged carbon atoms (Figures 6, 7).

**FIGURE 8:**
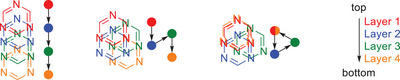
Illustration of the stacking patterns of α‐ammeline melanurate (**1·2α**) (left), β‐ammeline melanurate (**1·2β**) (center) and γ‐ammeline melanurate (**1·2γ**) in comparison.

The main difference between the modifications is the pattern of the staggering. In **1·2α**, the displacement occurs always in the same direction as in melamine cyanurate (AA staggering), whereas in **1·2β** and **1·2γ**, the direction of the displacement changes after each layer. In **1·2β**, the staggering pattern consists of two alternating displacement directions (AB staggering), while **1·2γ** has a spiral‐shaped staggering pattern (ABC staggering) (Figure 8). Strikingly, the staggering pattern of **1·2γ** results in the direction of its channels being essentially perpendicular to the molecular planes.

In contrast to melamine cyanurate, no completely ordered structure could be determined for either modification, and the molecules had to be refined as a superposition of all different possible orientations of **1** and **2** (Figures S9–S11). In melamine cyanurate, only two different stacking options are possible, namely stacking of cyanuric acid on cyanuric acid and melamine on melamine as well as alternating stacking of cyanuric acid and melamine (Figure S35). Performed theoretical calculations suggest that the latter stacking option is around 8 kJ/mol more stable than the first (Table S20), which is consistent with previous theoretical calculation results [27, 28]. For **1·2**, six different stacking options are feasible due to the three possible orientations of **1** and **2** (Figure S34). Theoretical calculations analogous to those carried out for melamine cyanurate indicate that the three stacking options with alternating stacking of **1** and **2** exhibit greater stability in comparison to the three stacking options with stacking of **1** on **1** and **2** on **2**. However, the determined energetic difference of around 1 kJ/mol is significantly lower than that of melamine cyanurate. Furthermore, within these two groups of stacking options, the energetic difference is even smaller (< 0.1 kJ/mol) (Table S19). These results help to explain why no fully ordered structure of **1·2** could be identified. Due to this disorder, XRD investigation alone could not rule out that the analyzed samples consisted partly or solely of melamine cyanurate, which is why the obtained products were dissolved in aqueous HNO_3_ (2 m) in large excess (≈ 40 eq.). After solvent evaporation, the composition of the resulting residue was analyzed by Rietveld refinement of its PXRD pattern, which revealed that it is comprised of ammelinium nitrate (**3**) and ammelidium nitrate (**4**) in an approximate molar 1:1 ratio (Figure S19).

For a better comparison with the determined structures of **2** and **1·2**, commercially purchased melamine and cyanuric acid as well as melamine cyanurate prepared therefrom were analyzed by PXRD (Figures S22–24 and Tables 2 and S18). Rietveld refinement showed that all modifications of **1·2** have a larger stacking distance between their layers than melamine cyanurate, which can be attributed to its ordered layer arrangement compared to **1·2**. Nevertheless, every modification of **1·2** has a greater density than melamine cyanurate, as the molecules within the layers are more densely packed. However, all three modifications of **1·2** have a lower density and a larger stacking distance than their two components **1** and **2**, which is in contrast to melamine cyanurate, whose density and stacking distance are roughly the average between those of melamine and cyanuric acid. Of the three modifications of **1·2**, **1·2α** possesses the highest density and lowest stacking distance, while both are approximately equal in the case of **1·2β** and **1·2γ**.

**TABLE 2:** Determined densities *ρ* and stacking distances *d*_π–π_ alongside the associated stacking reflections of the different compounds at 20 °C by Rietveld refinement.

Name	*ρ* [g/cm^3^]	*d*_π–π_ [Å] (*hkl*)
Melamine	1.570	3.398 (−103)
Ammeline (**1**) [14]	1.714	3.066 (−103)
Ammelide (**2**)	1.725	3.166 (102)
Cyanuric acid	1.773	2.999 (−101)
Melamine cyanurate	1.658	3.171 (101)
α‐Ammeline melanurate (**1·2α**)	1.676	3.179 (201)
β‐Ammeline melanurate (**1·2β**)	1.666	3.196 (002)
γ‐Ammeline melanurate (**1·2γ**)	1.666	3.201 (003)

### 2.3 Preparation and Structural Analysis of Ammelidium Nitrate and Perchlorate

**SCHEME 4:**
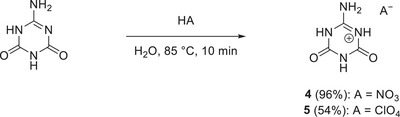
Synthetic conditions used for the preparation of ammelidium nitrate (**4**) and ammelidium perchlorate (**5**).

With access now to pure ammelide (**2**), its reactivity toward aqueous HNO_3_ and HClO_4_ (2 m each) was examined for the synthesis of ammelidium nitrate (**4**) and ammelidium perchlorate (**5**), respectively (Scheme 4).

**4** was obtained in near quantitative yield of 96%, as the resulting solution in aqueous HNO_3_ could easily be completely evaporated off at room temperature. This was not possible in the case of **5** due to the much lower volatility of HClO_4_ compared to HNO_3_. Even after gently heating at 50 °C in a crystallization dish for one week, a small amount of solution remained. However, storage of this solution in a fridge for 2 h resulted in the formation of a precipitate. Isolation of this precipitate gave **5** in 54% yield. Higher yields may be achievable when the solution is further narrowed down. For both compounds, Rietveld refinement confirmed the crystallographic phase purity of the bulk materials (Figures S27, S28).

**FIGURE 9:**
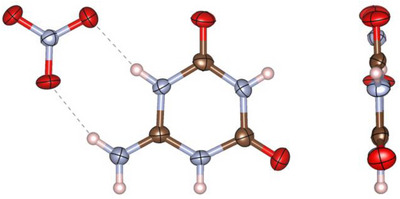
Asymmetric unit of ammelidium nitrate (**4**) in the crystal viewed perpendicular (left) and parallel (right) to the triazine plane. All atoms besides the H atoms are drawn as thermal ellipsoids at the 80% probability level (atomic coloring: H white, C brown, N light‐blue, O red).

**FIGURE 10:**
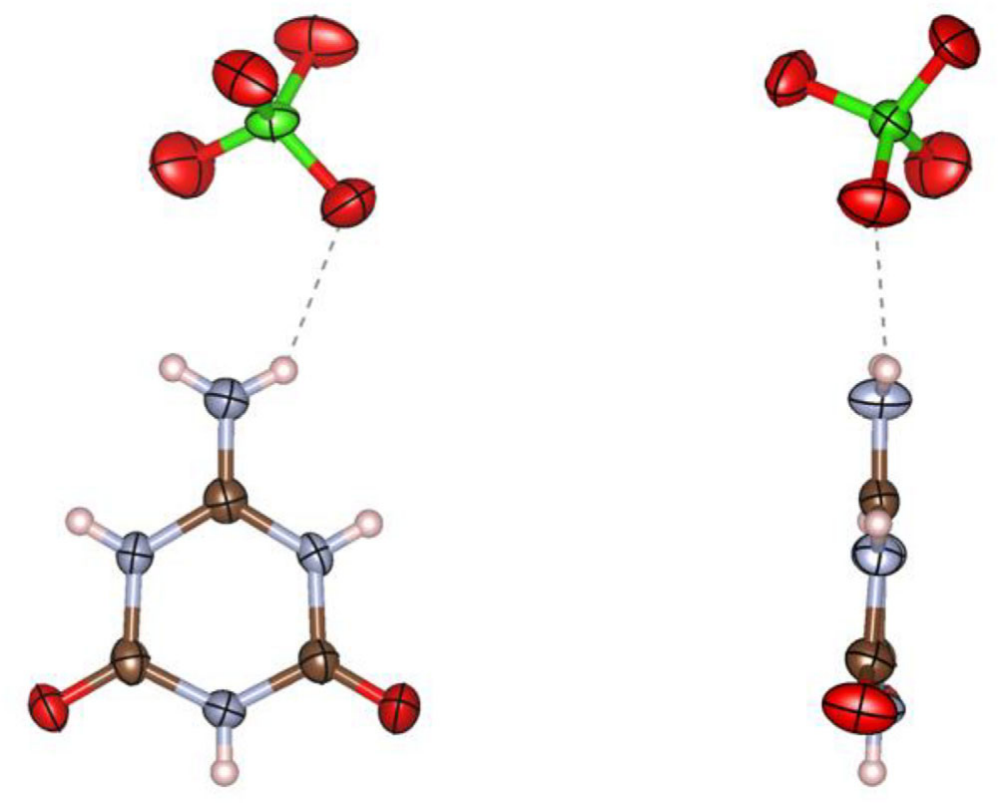
Asymmetric unit of ammelidium perchlorate (**5**) in the crystal viewed perpendicular (left) and parallel (right) to the triazine plane. All atoms besides the H atoms are drawn as thermal ellipsoids at the 80% probability level (atomic coloring: H white, C brown, N light‐blue, O red, Cl green).

Both **4** and **5** crystallize in space group *P*2_1_/*n* (no. 14; with *a* = 6.2224(4), *b* = 4.6285(2), *c* = 23.7842(15) Å; *β* = 94.006(2)° in the case of **4** and with *a* = 7.6704(5), *b* = 10.2444(6), *c* = 10.2540(6) Å; *β* = 90.731(2)° in the case of **5** at −100 °C, Tables S3, S14–S17) [34] with four formula units per unit cell. In both structures, the ammelidium cations occur exclusively in the same tautomeric form as **2**, indicative by the significantly shorter C─O bond lengths (1.202(2) and 1.212(2) Å in **4**; 1.207(2) and 1.209(2) Å in **5**) in comparison to the C─NH_2_ bond lengths (1.301(2) Å in **4**; 1.296(3) Å in **5**). However, all these bonds are significantly reduced in length when compared with the corresponding bonds of **2** due to protonation, a phenomenon that was also observed in the case of **1** [14]. In addition, the ammelidium cations have two almost identical OC─N(H)─C(NH_2_) bond angles in **4** (123.57(16)° and 124.04(16)°) and **5** (123.57(16)° and 123.66(16)°), which had very distinct values prior to protonation. As a result, the ammelidium cations exhibit an approximate *C*_2_ *_v_* molecular symmetry analogous to ammelinium [14, 20, 21] (Figures 9, 10).

**FIGURE 11:**
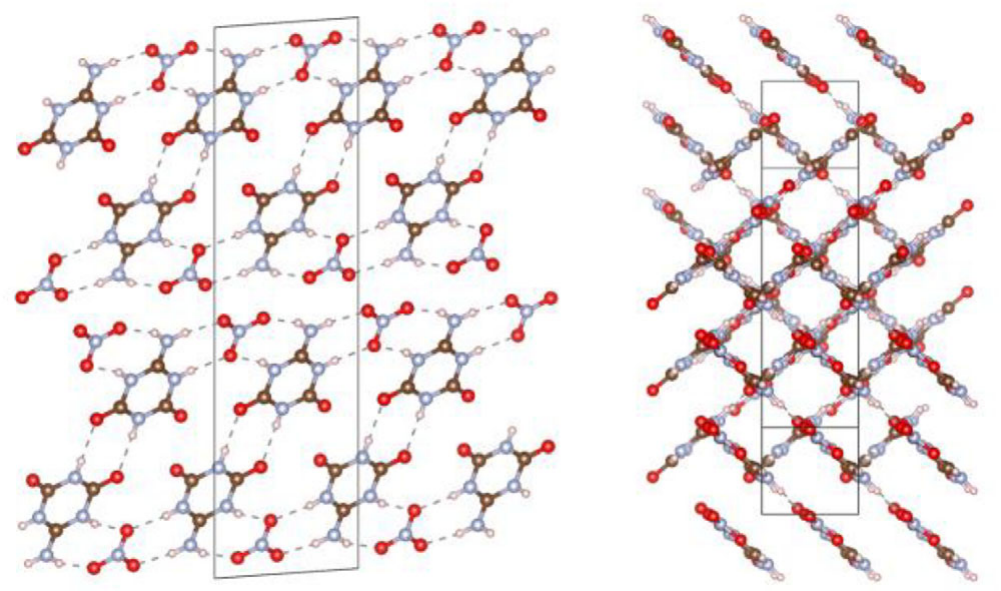
Crystal structure of ammelidium nitrate (**4**) viewed along [010] (left) and [−401] (right) (atomic coloring: H white, C brown, N light‐blue, O red).

The nitrate anions in **4** have an almost regular trigonal‐planar molecular geometry (i.e., *D*_3_ *_h_*), resulting in a narrow distribution of their O─N─O bond angles (119–121°) and N─O bond lengths (1.24–1.26 Å). Each nitrate anion forms hydrogen bonds to two different ammelidium cations via its oxygen atoms, resulting in infinite rows of alternating ammelidium and nitrate molecules parallel to either (114) or (1−14). These rows are bound to other rows with parallel orientation by π−π stacking, while they are connected to rows with different orientations via hydrogen bonding between oxygen and hydrogen atoms of ammelidium cations. Overall, this leads to a very efficiently packed structure, resulting in a density of **4** (1.858 g/cm^3^) that is significantly higher than that of ammelinium nitrate (**3**) (1.690 g/cm^3^) (Figure 11).

**FIGURE 12:**
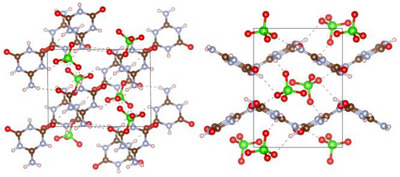
Crystal structure of ammelidium perchlorate (**5**) viewed along [010] (left) and [001] (right) (atomic coloring: H white, C brown, N light‐blue, O red, Cl green).

The perchlorate anions in **5** exhibit nearly regular tetrahedral molecular geometry (i.e., *T_d_*), as can be seen from the determined O─Cl─O bond angles (107–111°) and Cl─O bond lengths (1.42–1.46 Å). Within the structure of **5**, ammelidium cations are approximately parallel to either (130) or (−130) and form hydrogen bonds to each other and the perchlorate anions. In contrast to **4** and all modifications of ammelinium perchlorate, no form of π−π stacking is observable in **5**. As a result, its density of 1.884 g/cm^3^ was found to be lower than that of α‐ and β‐ammelinium perchlorate with a density of 1.900 and 1.922 g/cm^3^ [14], respectively (Figure 12).

### 2.4 Physicochemical Characterization

Differential thermal analysis (DTA) and thermogravimetric analysis (TGA) measurements were performed to analyze the thermal behavior of the synthesized compounds (Figures S29–S33). The synthesized ammelidium salts were additionally characterized on their sensitivities and energetic properties, which were compared to their ammelinium counterparts as well as to the commonly employed HEDMs 2,4,6‐trinitrotoluene (TNT) [40], 2,4,6‐triamino‐1,3,5‐trinitrobenzene (TATB) [40], and 1,3,5‐trinitro‐1,3,5‐triazinane (RDX) [40] (Tables 3, S21–S24).

Ammelide (**2**) showed high thermal stability, as it only lost mass significantly above 360 °C, which is later than melamine (305.15 °C) [41] and cyanuric acid (310 °C) [42], but earlier than ammeline (**1**) (390 °C) [14]. For ammeline melanurate (**1·2**), the significant mass loss began at 390 °C and thus considerably later compared to melamine cyanurate (334.14 °C) [43]. As in the case of **1**, the mass losses of **2** and **1·2** were accompanied by endothermic peaks. In both cases, no significant material resublimation was observed during the weight loss, and a yellow amorphous solid resembling melon in appearance remained after the thermal treatment. Ammelinium nitrate (**3**) decreased in mass in two major steps. The first occurred at 225–275 °C, in which the sample was reduced to around 67% of its initial mass, matching well with the expected mass loss for HNO_3_. The second one started at 380 °C, and thus at a similar temperature at which the mass loss of **1** set in. The DTA was found to feature an endothermic peak corresponding to this mass loss. Additionally, an endothermic peak at 300 °C immediately followed by an exothermic peak was observed, which fits the previously reported decomposition temperature of **3** (303.3 °C) [20]. In the case of ammelidium nitrate (**4**), a significant mass loss was detected at 130–215 °C, which was accompanied by an endothermic peak. Analogous to **3**, this mass loss can be attributed to the release of HNO_3_. Starting from the decomposition temperature of **2**, a similar series of endothermic peaks was observed with a simultaneous substantial increase of the mass loss rate. For ammelidium perchlorate (**5**), the mass loss began at 180 °C, which was accompanied by an exothermic peak. Additionally, the DTA showed a pair of exothermic peaks and an endothermic peak at 240 °C and 325 °C, respectively.

**TABLE 3:** Sensitivities, densities, decomposition temperatures, and calculated energetic properties of the nitrate and perchlorate salts of ammelinium (i.e., C_3_H_6_N_5_O^+^) and ammelidium (i.e., C_3_H_5_N_4_O_2_^+^) compared to TNT, TATB, and RDX.

Compound	(C_3_H_6_N_5_O)NO_3_ (3)	(C_3_H_5_N_4_O_2_)NO_3_ (4)	β‐(C_3_H_6_N_5_O)ClO_4_ [14]	(C_3_H_5_N_4_O_2_)ClO_4_ (5)	TNT [40]	TATB [40]	RDX [40]
IS^a^ [J]	>40	>40	>40	>40	15	>40	7.5
FS^b^ [N]	>360	>360	>360	>360	>360	>360	120
ESD^c^ [J]	>1.5	>1.5	>1.5	>1.5	>1.5	>1.5	0.2
*ρ* ^d^ [g/cm^3^]	1.66	1.82	1.89	1.85	1.65	1.94	1.80
*T*_dec_ ^e^ [°C]	225	130	240	180	290	350	210
Δ_f_ *H*°_(s)_ ^f^ [kJ/mol]	−497.9	−635.8	−563.8	−656.7	−59.4	−154.0	70.3
Δ_f_ *U*°_(s)_ ^g^ [kJ/mol]	−478.1	−617.2	−542.8	−637.9	−42.0	−139.7	92.6
Δ_Ex_ *U*°^h^ [kJ/kg]	−1881	−2085	−2466	−2824	−4359	−3840	−5699
*T*_det_ ^i^ [K]	1771	1891	2199	2494	3165	2747	3736
*p*_det_ ^j^ [kbar]	160	204	217	218	185	278	340
*v*_det_ ^k^ [m/s]	6764	7337	7388	7284	6804	8193	8807

^a^ Impact sensitivity (BAM drophammer (1 of 6)).

^b^ Friction sensitivity (BAM friction tester (1 of 6)).

^c^ Electrostatic discharge sensitivity (OZM XSpark 10 (1 of 6)).

^d^ Room temperature density.

^e^ Decomposition temperature.

^f^ Solid‐state enthalpy of formation.

^g^ Solid‐state energy of formation.

^h^ Heat of detonation.

^i^ Detonation temperature.

^j^ Detonation pressure.

^k^ Detonation velocity.

The conducted sensitivity measurements demonstrated that compounds **3–5** have no relevant sensitivities against friction, impact and electrostatic discharge in the same way as ammelinium perchlorate and TATB, but in contrast to TNT and RDX. Of these three compounds, **3** was calculated to have the by far lowest detonation pressure and velocity, slightly below those of TNT, which can be attributed to its comparatively much lower density and poorer oxygen balance. On the other hand, the calculated detonation pressure and velocity of **4** and **5** were determined to be similar to those of β‐ammelinium perchlorate and thus significantly higher than those of TNT, but still lower than those of TATB and RDX.

## 3 Conclusion

Ammelide (**2**), also known as melanuric acid, is a simple molecular compound, which has been mentioned in over 800 different scientific works to date [44]. The term ammelide was conceived by Liebig almost 200 years ago, which, however, was initially not used to describe **2**, but rather a 1:1 molar mixture of **2** with the closely related ammeline (**1**).

In this work, a structural description of **2** was presented for the first time, thereby eliminating the last missing crystal structure of the most prevalent hydrolysis and pyrolysis products of melamine. Moreover, this unequivocally demonstrated that **2** occurs exclusively as 6‐amino‐1,3,5‐triazine‐2,4(1*H*,3*H*)‐dione in the solid, while also showing that **2** adopts a layered structure with a distorted honeycomb motif within the layers, analogous to **1**. The sample of **2** used in the crystallization experiments under hydrothermal conditions for its structural analysis was isolated from the aforementioned mixture between **1** and **2**, originally referred to by Liebig as ammelide [1]. By employing crystallization conditions analogous to those used for **2**, it was possible to confirm by SCXRD that this material is not just an arbitrary mixture between **1** and **2**, but a distinct compound, namely their 1:1 adduct ammeline melanurate (**1·2**). It was found that **1·2** forms layers with a honeycomb motif and hexagonal voids analogous to melamine cyanurate. Remarkably, three distinct modifications of **1·2** were successfully structurally elucidated. The presented straightforward synthesis method, alongside its higher thermal stability and structural variety, should make **1·2** a viable or possibly superior alternative to melamine cyanurate as a flame retardant as well as for molecule and nanoparticle adsorption.

Despite its amphoteric properties, no structural data on any salts of **2** have been published to date. Therefore, first efforts were made to remedy this critical lack of knowledge. Ammelidium nitrate (**4**) and ammelidium perchlorate (**5**) were chosen as the salts of **2** to be investigated, since it was figured that they might be suitable as insensitive HEDM with improved energetic properties compared to their ammelinium counterparts due to their better oxygen balance. The performed theoretical calculations based on their solved crystal structures indicate that, while the energetic properties of **5** do not improve in comparison to ammelinium perchlorate, the detonation pressure and velocity of **4** are significantly higher than those of ammelinium nitrate (**3**). However, although **4** and **5** also showed no detectable sensitivities against friction, impact and electrostatic discharge, their thermal stabilities turned out to be much lower compared to their ammelinium counterparts. It can be concluded that in some cases improvements in the energetic properties can be achieved by replacing **1** with **2**, but at the expense of the compound's thermal stability, resulting in far lower decomposition temperatures compared to TATB, which is considered the golden standard for insensitive HEDM [45].

Overall, this study gives an extensive overview of the structural and physicochemical properties of **2** and some of its compounds, not only resolving the long‐standing scientific debate surrounding the term ammelide [22, 23, 24, 46, 47, 48, 49], but also providing first insights into the potential of **2**‐containing compounds for future functional materials.

## Conflicts of Interest

The authors declare no conflict of interest.

## Supporting information



The authors have cited additional references within the Supporting Information [1–35].

Supporting File 1: chem70637‐sup‐0002‐SuppMat.zip

## Data Availability

The data that support the findings of this study are available in the supplementary material of this article.
