# Supplementary material for: Resolving a Nearly Two‐Centuries‐Old Mystery: On the Structural Chemistry and Physicochemical Properties of Compounds Associated With the Term Ammelide
Source: Chemistry. 2025 Dec 31;32(9):e03587. doi: 10.1002/chem.202503587 (PMC12958097; doi:10.1002/chem.202503587)
Supplement: Supplementary file 1 — The authors have cited additional references within the Supporting Information [1–35]. [file CHEM-32-e03587-s002.pdf]

Supporting Information
©Wiley-VCH 2025
69451 Weinheim, Germany

Resolving a Nearly Two-Centuries-Old Mystery: On the Structural Chemistry and Physicochemical Properties of Compounds Associated with the Term Ammelide

Thaddäus J. Koller, Kristian Witthaut, Reinhard M. Pritzl, Simon M. J. Endraß, Markus Rösch, Georg Krach, Nadine Lammer, Thomas M. Klapötke, and Wolfgang Schnick

Ammelide, also known as melanuric acid, is a simple molecular compound, which can be regarded as the double hydrolysis product of the industrially relevant compound melamine. Within this work, the first structural description of ammelide was provided almost 200 years after its discovery. This not only gave the first unambiguous evidence that ammelide's preferred tautomeric form in the solid is 6-amino-1,3,5-triazine-2,4(1*H*,3*H*)-dione, but also showed that ammelide adopts a layered structure similar to the closely related ammeline. Moreover, the crystal structures of three modifications of the 1:1 adduct between ammeline and ammelide were elucidated, which form layers with a honeycomb motif and hexagonal voids analogous to melamine cyanurate. Differential thermal analysis and thermogravimetric analysis demonstrated that both pure ammelide and its adduct with ammeline have exceptionally high thermal stabilities, resulting from their dense hydrogen bonding networks. Finally, the synthesis and structural characterization of the ammelide's nitrate and perchlorate salt was carried out to examine the potential of ammelide as part of insensitive high-energy-density materials through sensitivity measurements and theoretical calculations.

DOI: 10.1002/chem.2025XXXXX

Table of Contents

Deployed Analysis Methods **3**

Fourier Transformed Infrared Spectroscopy (FTIR) 3

Elemental Analysis (EA) 3

Single Crystal X-Ray Diffraction (SCXRD) 3

Powder X-Ray Diffraction (PXRD) 3

Differential Thermal Analysis (DTA) and Thermogravimetric Analysis (TGA) 3

Sensitivity Characterization 3

Computational Details **4**

Calculations Regarding Stacking Arrangements 4

Calculations Regarding Detonation Parameters 5

Experimental Procedures **6**

Information on the Chemicals used in the Experiments 6

Preparation of Guanylurea Hydrochloride 6

Preparation of Ammeline Melanurate (1·2) 6

Resolution of Ammeline Melanurate (1·2) into Ammeline (1) and Ammelide (2) 6

Preparation of Crystalline Ammeline Melanurate (1·2) and Ammelide (2) 7

Preparation of Melamine Cyanurate 7

Preparation of Ammelinium Nitrate (3) 7

Preparation of Ammelidium Nitrate (4) 7

Preparation of Ammelidium Perchlorate (5) 7

Analytical Results **8**

FTIR Spectra 8

SCXRD Data 12

PXRD Data 26

DTA and TGA Curves 33

Computational Results 36

References **39**

Author Contributions **40**

Deployed Analysis Methods

Fourier Transformed Infrared Spectroscopy (FTIR)

FTIR spectra were recorded in a wavenumber range of 4400–650 cm^{-1} using a *Perkin Elmer Spectrum BX-II* equipped with a *Smiths Detection DuraSampl IR II Diamond ATR* sensor. The spectra were visualized with the program *Origin 2019b*.[1] The wavenumbers $\tilde{\nu}$ of the most significant absorption bands are reported in cm^{-1}.

Elemental Analysis (EA)

For the determination of C, H and N contents, combustion analyses were performed using an *Elementar vario el* or *Elementar vario micro* instrument. Cl contents were determined by potentiometric titration with $AgNO_3$ on a *Metrohm Titrando 888*. All values are reported in wt.% alongside the theoretically expected values calculated by the program *ChemDraw 20*.[2]

Single Crystal X-Ray Diffraction (SCXRD)

SCXRD measurements for the determination of crystal structures were conducted on a *Bruker D8 Venture* (Mo-K_α radiation) diffractometer. Raw data integration and absorption correction was done using the program *APEX3*.[3] Structure solutions were performed with *SHELXT-2018/2* and the resulting solutions were refined with *SHELXL-2018/3*.[4] Structure visualization was done with the program *VESTA 3.4.6*.[5]

Powder X-Ray Diffraction (PXRD)

PXRD measurements at 20 °C on samples filled in glass capillaries (Ø 0.50 mm) were performed on a *Stoe Stadi-P* diffractometer (Cu-$K_{\alpha 1}$ radiation) equipped with a Ge(111)-monochromator and *Dectris Mythen 1 K* silicon strip detector. Theoretical PXRD patterns for comparison were created with the program *WinXPow 3.0.2.1*.[6] For quantitative phase analysis of the products and determination of the compounds' cell parameters at 20 °C, the obtained PXRD patterns were analyzed by the Rietveld method[7] (i.e. Rietveld refinement) using the program *TOPAS 6*.[8] All experimental PXRD patterns and Rietveld refinement results were visualized with *Origin 2019b*.[1]

Differential Thermal Analysis (DTA) and Thermogravimetric Analysis (TGA)

DTA and TGA measurements with a heating rate of 5 °C/min were conducted on an *OZM Research DTA 552-Ex* and a *Perkin Elmer TGA4000* instrument, respectively. The TGA measurements were carried out at a constant N_2 gas flow of 20 ml/min. The stated values are the onset temperatures and were rounded to the closest multiple of 5. The resulting data was visualized using *Origin 2019b*.[1]

Sensitivity Characterization

Sensitivities toward impact (IS), friction (FS) and electric discharge (ESD) were determined using a *BAM drop hammer*, a *BAM friction apparatus* and an *OZM XSpark 10*, respectively, applying the 1 of 6 method.

Computational Details

Calculations Regarding Stacking Arrangements

The ground state energies of each stacking option (U_0) were calculated from ordered structure models of ammeline melanurate (**1·2**) and melamine cyanurate using the *Vienna ab initio simulation package Version 5.4.4* (VASP) based on periodic density functional theory calculations.[9] Core and valence electrons are separated exploiting projector-augmented waves (PAW).[10] The exchange and correlation energy is calculated using the generalized gradient approximation (GGA), as described by Perdew, Burke and Ernzerhof (PBE).[11] Non-spherical contributions to the gradient of the density in the PAW spheres are included. An additional dispersion correction term was calculated within the DFT-D3 method with Becke-Johnson damping function[12] to properly consider van der Waals (vdW) interactions between the organic molecules.

For the optimization the Brillouin zones were sampled on a 2x1x5 Γ-centered *k*-point grid (resolution ~0.2/Å). For this the RMM-DIIS algorithm, as originally described by Pulay was used.[13] The optimization was done with full ionic degrees of freedom, i.e. atomic positions, cell shape and cell volume. The interpolation of the *k*-space was done via tetrahedron method with Blöchl corrections. The energy convergence criterion was set to 10^{-5} eV and the residual atomic forces were relaxed until the convergence criterion of 10^{-4} eV/Å was reached. For all calculations a plane wave energy cutoff of 600 eV was used.

The optimized layers were then isolated into two, infinitely extending layers, for which static calculations were performed with a convergence criterion of 10^{-5} eV and Gaussian smearing. In these calculations, a 6x2x4 Γ-centered *k*-point grid was employed.

The difference between the calculated energies was used to compare different stacking options as indicated by the relative energetic difference (ΔU_0).

Calculations Regarding Detonation Parameters

Electronic molecular and atomic enthalpies ($H°$(M) and $H°$(A)) were calculated with the program *Gaussian 16*[14] using the CBS-QB3 method, which was modified via inclusion of diffuse functions in the geometry optimization step.[15] CBS-QB3 begins with geometry optimization and subsequent frequency calculations at the B3LYP level. Afterwards, single-point calculations at the CCSD(T) and MP4SDQ level are performed. The final values are obtained by extrapolation to the infinite basis set limit using pair natural orbital energies at the MP2 level as well as an additive correction to the CCSD(T) level. The obtained $H°$(M) and $H°$(A) values were utilized alongside the atomic gas phase enthalpies of formation ($\Delta_f H°_{(g)}$(A)) according to the Active Thermochemical Tables (ATcT) Ver. 1.130[16] to determine the molecular gas phase enthalpies of formations ($\Delta_f H°_{(g)}$(M)) by the atomization method (Equation S1, Table S21 and S22).

$$\Delta_f H°_{(g)}(\mathrm{M}) = H°(\mathrm{M}) - \sum H°(\mathrm{A}) + \sum \Delta_f H°_{(g)}(\mathrm{A}) \quad \text{(S1)}$$

The ground state energies of each compound (U_0(F)) and of each isolated molecule (U_0(M)) were calculated outgoing from the solid state structure models as determined by SCXRD using the *Vienna ab initio simulation package Version 5.4.4* (VASP) based on periodic density functional theory calculations.[9] All structures were optimized as described in the previous section.[10–13] For the isolated molecules, static calculations were performed with the same convergence criterion as for the isolated double layers, but using a Γ-only grid.

Ultimately, a compound's lattice energy (ΔU_L) was determined by the difference of U_0(F) and the sum of U_0(M) of all molecules M per formula F (Equation S2, Table S23), from which the lattice enthalpy (ΔH_L) could be derived (Equation S3).

$$\Delta U_L = U_0(\mathrm{F}) - \sum U_0(\mathrm{M}) \quad \text{(S2)}$$

$$\Delta H_L = U_L(\mathrm{F}) + \Delta n_L \mathrm{R} T° \quad \text{(S3)}$$

The solid-state enthalpy of formation ($\Delta_f H°_{(s)}$) of each compound was determined from ΔH_L and the sum of $\Delta_f H°_{(g)}$(M) of all molecules per formula (Equation S4), which were used to calculate the solid-state energies of formation ($\Delta_f U°_{(s)}$) (Equation S5, Table S24).

$$\Delta_f H°_{(s)} = \sum \Delta_f H°_{(g)}(\mathrm{M}) + \Delta H_L \quad \text{(S4)}$$

$$\Delta_f U°_{(s)} = \Delta_f H°_{(s)} - \Delta_f n \mathrm{R} T° \quad \text{(S5)}$$

From these values and the room temperature densities derived from Rietveld refinement (Table S18), the compounds' detonation parameters at the Chapman-Jouguet point were calculated with the program *EXPLO5 V7.01.01*.[17]

Experimental Procedures

Information on the Chemicals used in the Experiments

Table S1. Formula, commercial source and purity of the chemicals and solvents used in the following syntheses.

Compound	Formula	Source	Purity
Dicyandiamide	$C_2H_4N_4$	Acros Organics	99.5%
Melamine	$C_3H_6N_6$	Sigma Aldrich	99%
Cyanuric acid	$C_3H_3N_3O_3$	Sigma Aldrich	98%
Potassium cyanate	KOCN	Acros Organics	97%
Dipotassium phosphate	K_2HPO_4	Grüssing GmbH	99%
Sodium carbonate	Na_2CO_3	Staub & Co. - Silbermann	technical
Aqueous hydrochloric acid (2 M)	HCl (aq.)	VWR	technical
Aqueous phosphoric acid (85 wt.%)	H_3PO_4 (aq.)	VWR	technical
Aqueous perchloric acid (60 wt.%)	$HClO_4$ (aq.)	VWR	technical
Aqueous nitric acid (2 M)	HNO_3 (aq.)	VWR	technical
Diethyl ether	$C_4H_{10}O$	Staub & Co. - Silbermann	≥99.5%

Preparation of Guanylurea Hydrochloride

Dicyandiamide (6.73 g, 80.0 mmol, 1.0 eq.) was stirred in aqueous HCl (200 ml, 2 M, 400 mmol, 5.0 eq.) at room temperature and then at 110 °C under reflux for 15 min each. The resulting solution was transferred into a crystallization dish and allowed to evaporate at room temperature. To remove the last remnants of solvent, the residue was finely ground and subsequently dried at 80 °C for 12 h, which yielded the title compound (11.0 g, 79.4 mmol, 99%) as a white solid.
FTIR (neat, ATR, cm^{-1}): $\tilde{\nu}$ = 3147, 3009, 2923, 1758, 1728, 1683, 1639, 1610, 1578, 1524, 1461, 1342, 1133, 1112, 1046, 729, 684.
EA (wt.%): calc. for $C_2H_7ClN_4O$: C 17.34, H 5.09, Cl 25.59, N 40.44; found: C 17.21, H 4.87, Cl 25.53, N 40.45.

Preparation of Ammeline Melanurate (1·2)

Guanylurea hydrochloride (6.93 g, 50.0 mmol, 1.0 eq.) and KOCN (4.06 g, 50.0 mmol, 1.0 eq.) were finely ground together using an agate mortar and pestle. The resulting fine powder was transferred into a corundum crucible, which was subsequently covered with a lid. The corundum crucible was placed into a muffle furnace, in which it was heated at 160 °C for 12 h. The resulting crude product was removed from the crucible, ground again and stirred in boiling H_2O (250 ml) for 1 h. After cooling to room temperature, the resulting suspension was suction filtered, followed by washing of the residue with H_2O (3 × 50 ml), which yielded the title compound (2.64 g. 10.3 mmol, 41%) as a white solid.
FTIR (neat, ATR, cm^{-1}): $\tilde{\nu}$ = 3313, 3125, 2853, 2632, 1730, 1667, 1543, 1503, 1434, 1267, 1188, 1170, 1101, 1045, 985, 880, 772, 693, 665.
EA (wt.%): calc. for $C_6H_9N_9O_3$: C 28.24 H 3.55, N 49.40; found: C 27.72, H 3.62, N 48.37.

Resolution of Ammeline Melanurate (1·2) into Ammeline (1) and Ammelide (2)

Finely ground ammeline melanurate (**1·2**) (1.28 g, 5.00 mmol, 1.0 eq.) was recrystallized from boiling H_3PO_4 (0.2 M, 500 ml). The resulting suspension was suction filtered, followed by washing of the residue with H_2O (3 × 10 ml).
The residue was recrystallized a second time from boiling H_3PO_4 (0.2 M, 400 ml). The resulting suspension was suction filtered, followed by washing of the residue with additional H_2O (3 × 10 ml), yielding ammelide (**2**) (395 mg, 3.08 mmol, 62%) as a white solid.
FTIR (neat, ATR, cm^{-1}): $\tilde{\nu}$ = 3286, 3116, 2811, 2601, 1809, 1731, 1699, 1677, 1642, 1557, 1534, 1460, 1415, 1266, 1180, 1092, 1044, 987, 906, 864, 774, 658.
EA (wt.%): calc. for $C_3H_4N_4O_2$: C 28.13, H 3.15, N 43.74; found: C 27.89, H 3.24, N 43.55.

The filtrate was brought to a pH of 7 by dropwise addition of aqueous NH_3 (25 wt.%), after which the resulting suspension was suction filtered, followed by washing of the residue with H_2O (3 × 10 ml). The residue was then recrystallized from boiling Na_2CO_3 (0.2 M, 200 ml). The resulting suspension was suction filtered, followed by washing of the residue with H_2O (3 × 10 ml). Subsequently, the residue was recrystallized a second time from boiling Na_2CO_3 (0.2 M, 200 ml). The resulting suspension was suction filtered, followed by washing of the residue with additional H_2O (3 × 10 ml), yielding ammeline (**1**) (352 mg, 2.77 mmol; 55%) as a white solid.
FTIR (neat, ATR, cm^{-1}): $\tilde{\nu}$ = 3465, 3084, 2863, 2654, 1715, 1685, 1610, 1508, 1446, 1410, 1165, 1038, 991, 868, 788, 685.
EA (wt.%): calc. for $C_3H_5N_5O$: C 28.35, H 3.97, N 55.10; found: C 27.95, H 4.03, N 53.37.

Preparation of Crystalline Ammeline Melanurate (1·2) and Ammelide (2)

For the preparation of single crystals suitable for SCXRD, either ammeline melanurate (**1·2**) (255 mg, 1.00 mmol, 1.0 eq.) or ammelide (**2**) (258 mg, 2.00 mmol, 1.0 eq.) was recrystallized from aqueous K_2HPO_4 (0.4 M, 50 ml) inside of a Teflon lined autoclave with 50 ml capacity. In the case of ammeline melanurate (**1·2**), a 1:1 molar mixture of ammeline (**1**) (127 mg, 1.00 mmol, 1.0 eq.) and ammelide (**2**) (128 mg, 1.00 mmol, 1.0 eq.) can also be used as starting material. The closed autoclave was heated to 150 °C with a heating rate of 100 °C/h, kept at this temperature for 12 h and subsequently cooled to room temperature using a cooling rate 1 °C/h. The resulting crystalline material was suction filtered off and washed with H_2O (3 × 10 ml).

Preparation of Melamine Cyanurate

Melamine (126 mg, 1.00 mmol, 1.0 eq.) and cyanuric acid (129 mg, 1.00 mmol, 1.0 eq.) were added to H_2O (50 ml) inside of a Teflon lined autoclave with 50 ml capacity. After closing, the autoclave was heated to 150 °C with a heating rate of 100 °C/h, kept at this temperature for 12 h and subsequently cooled to room temperature using a cooling rate 1 °C/h. The resulting suspension was suction filtered and the residue washed with H_2O (3 × 10 ml), yielding the title compound (148 mg, 0.58 mmol, 58%) as a white solid.
FTIR (neat, ATR, cm^{-1}): $\tilde{\nu}$ = 3339, 3267, 3169, 3042, 2974, 2901, 2778, 1838, 1753, 1686, 1660, 1603, 1531, 1399, 1337, 1202, 1114, 1047, 980, 796, 769, 687.
EA (wt.%): calc. for $C_6H_9N_9O_3$: C 28.24 H 3.55, N 49.40; found: C 28.07, H 3.72, N 49.56.

Preparation of Ammelinium Nitrate (3)

Ammeline (**1**) (254 mg, 2.00 mmol, 1.0 eq.) was dissolved in aqueous HNO_3 (2 M, 10 ml, 20.0 mmol, 10 eq.) by stirring at 85 °C for 10 min. The resulting solution was placed into a fridge at 4 °C for 12 h. The resulting suspension was filtered and was washed with diethyl ether (2 × 20 ml), yielding the title compound (282 mg, 1.48 mmol, 74%) as a white solid.
FTIR (neat, ATR, cm^{-1}): $\tilde{\nu}$ = 3339, 3267, 3169, 3042, 2974, 2901, 2778, 1838, 1753, 1686, 1660, 1603, 1531, 1399, 1337, 1202, 1114, 1047, 980, 796, 769, 687.
EA (wt.%): calc. for $C_3H_6N_6O_4$: C 18.95, H 3.18, N 44.20; found: C 18.95, H 3.19, N 44.11.

Preparation of Ammelidium Nitrate (4)

Ammelide (**2**) (256 mg, 2.00 mmol, 1.0 eq.) was dissolved in aqueous HNO_3 (2 M, 20 ml, 40.0 mmol, 20 eq.) by stirring at 85 °C for 10 min. The resulting solution was placed into a crystallization dish and allowed to evaporate at room temperature. The residue was added to diethyl ether (50 ml), in which it was gently stirred for 1 min and then suction filtered off, yielding the title compound (365 mg, 1.92 mmol, 96%) as a white solid.
FTIR (neat, ATR, cm^{-1}): $\tilde{\nu}$ = 3335, 3233, 3138, 2996, 2774, 1795, 1778, 1727, 1685, 1562, 1418, 1368, 1321, 1250, 1067, 1050, 998, 823, 757, 722.
EA (wt.%): calc. for $C_3H_5N_5O_5$: C 18.86, H 2.64, N 36.65; found: C 18.86, H 2.56, N 36.59.

Preparation of Ammelidium Perchlorate (5)

Ammelide (**2**) (256 mg, 2.00 mmol, 1.0 eq.) was dissolved in aqueous $HClO_4$ (2 M, 10 ml, 20.0 mmol, 10 eq.) by stirring at 85 °C for 10 min. The resulting solution was placed into a crystallization dish and was narrowed down by heating at 50 °C for one week. Subsequently, the solution was stored in a fridge at 4 °C for 2 h, leading to the formation of a precipitate, which was removed from the remaining solution and added to diethyl ether (50 ml). The resulting suspension was gently stirred for 1 min and then suction filtered off, yielding the title compound (247 mg, 1.08 mmol, 54%) as a white solid.
FTIR (neat, ATR, cm^{-1}): $\tilde{\nu}$ = 3350, 3256, 3179, 2823, 1805, 1739, 1696, 1609, 1579, 1450, 1417, 1374, 1267, 1109, 1049, 1011, 993, 931, 755, 692, 669.
EA (wt.%): calc. for $C_3H_5ClN_4O_6$: C 15.77, H 2.21, Cl 15.51, N 24.52; found: C 15.79, H 2.35, Cl 15.69, N 24.58.

Analytical Results

FTIR Spectra

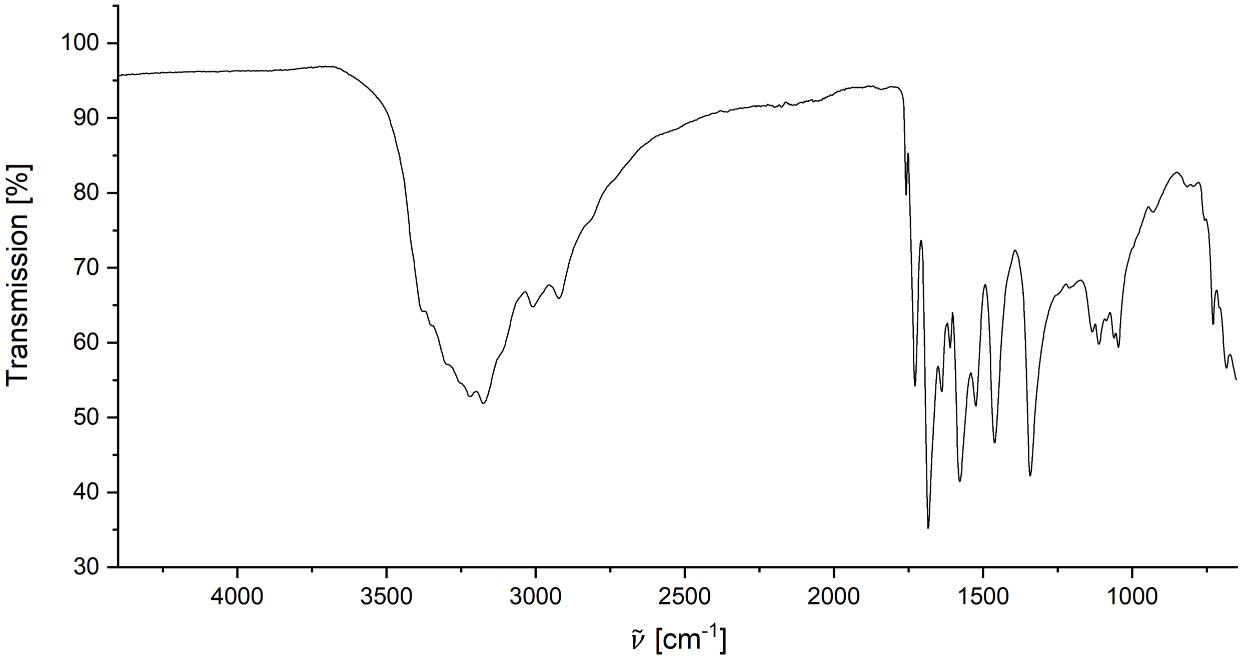

Figure S1. FTIR spectrum of guanylurea hydrochloride.

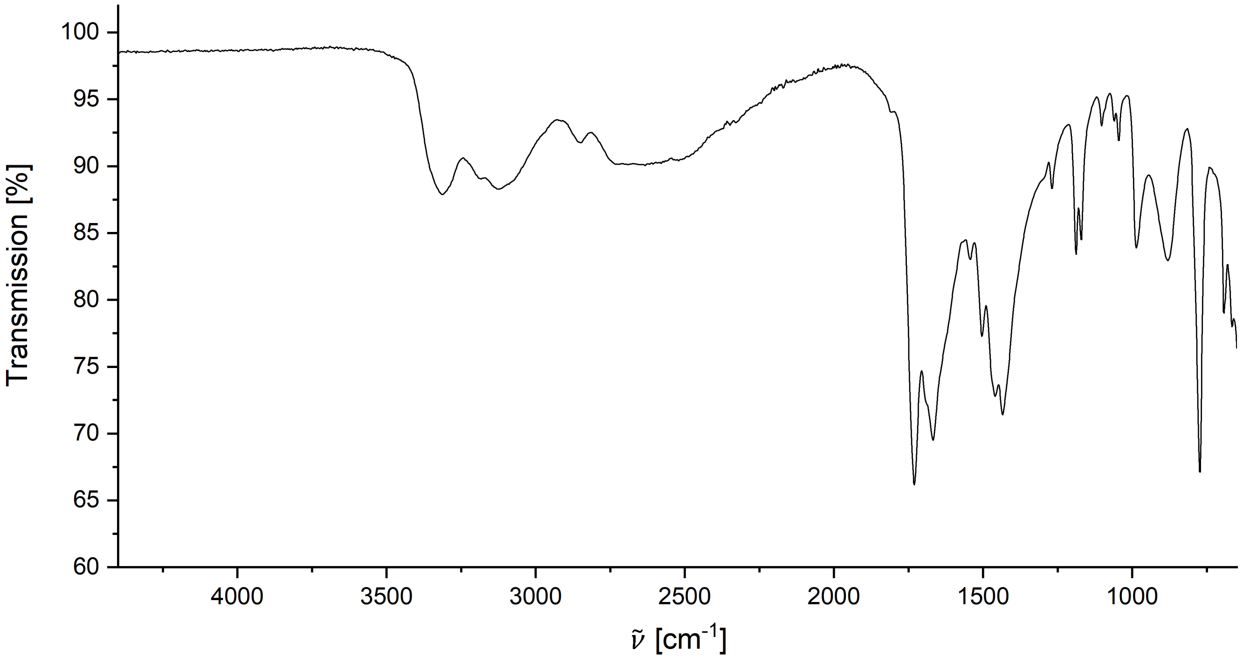

Figure S2. FTIR spectrum of ammeline melanurate (**1·2**).

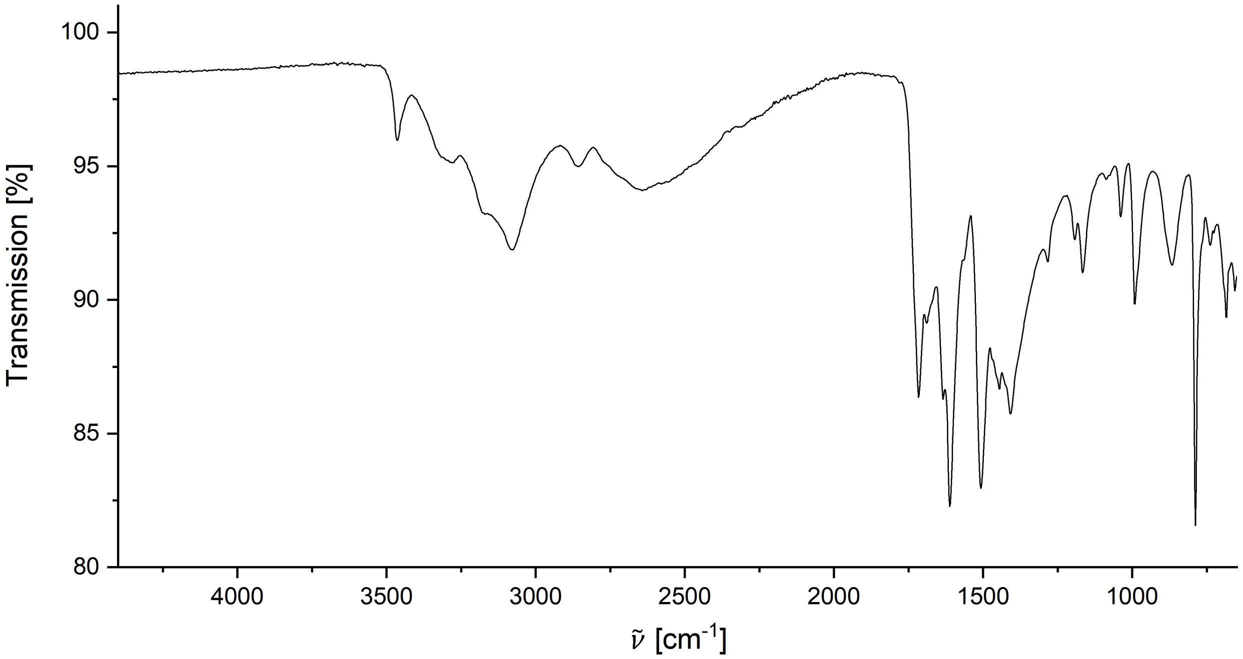

Figure S3. FTIR spectrum of ammeline (**1**).

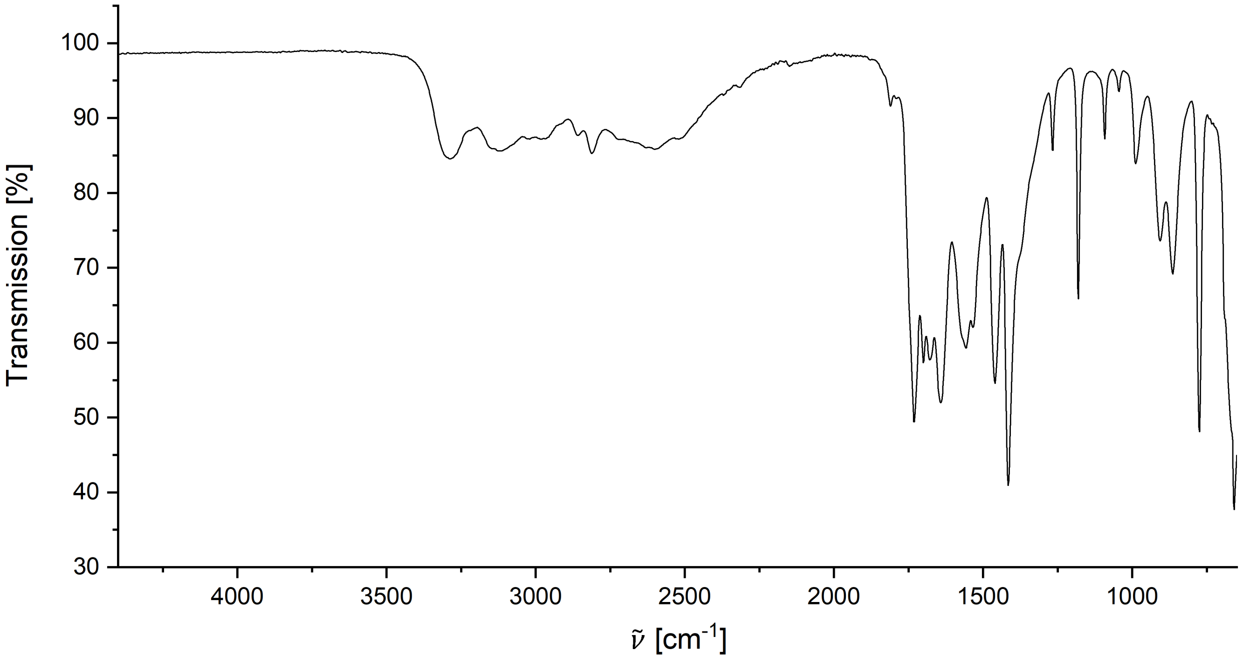

Figure S4. FTIR spectrum of ammelide (**2**).

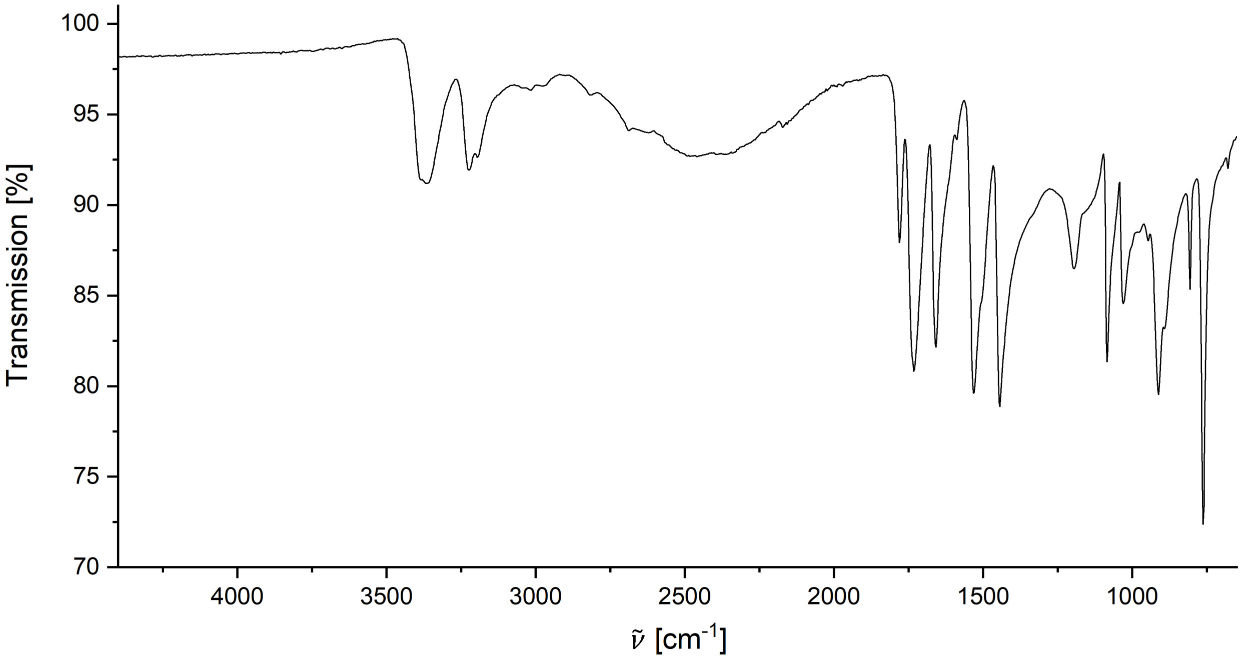

Figure S5. FTIR spectrum of melamine cyanurate.

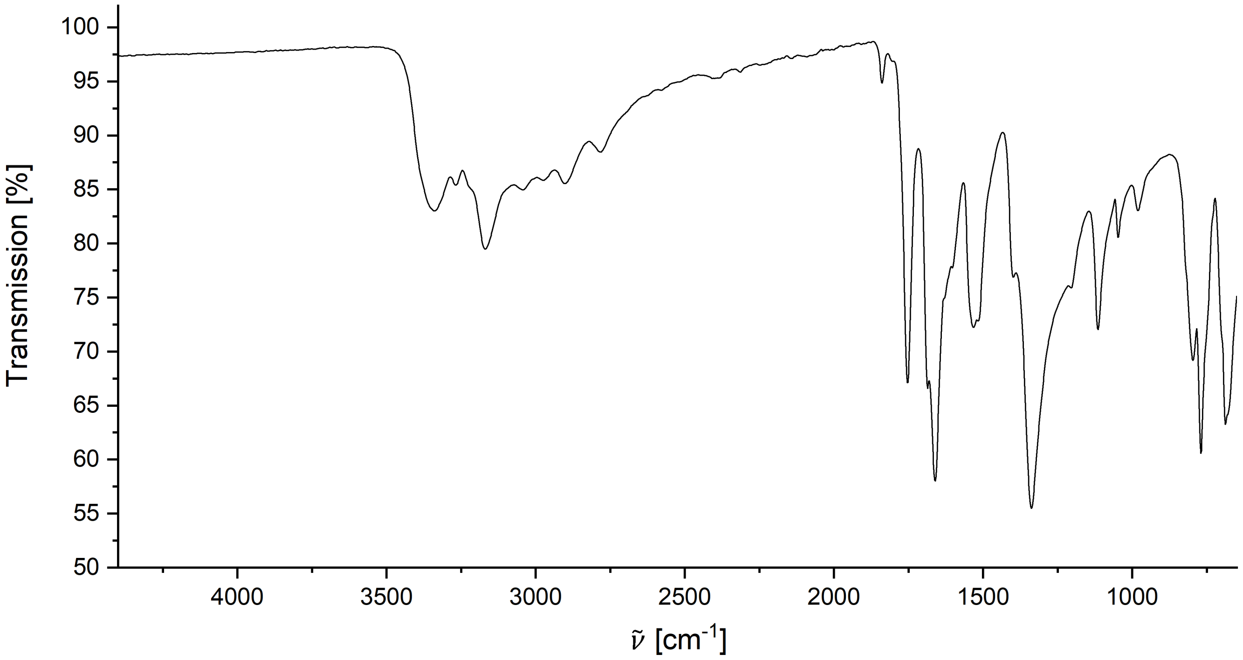

Figure S6. FTIR spectrum of ammelinium nitrate (**3**).

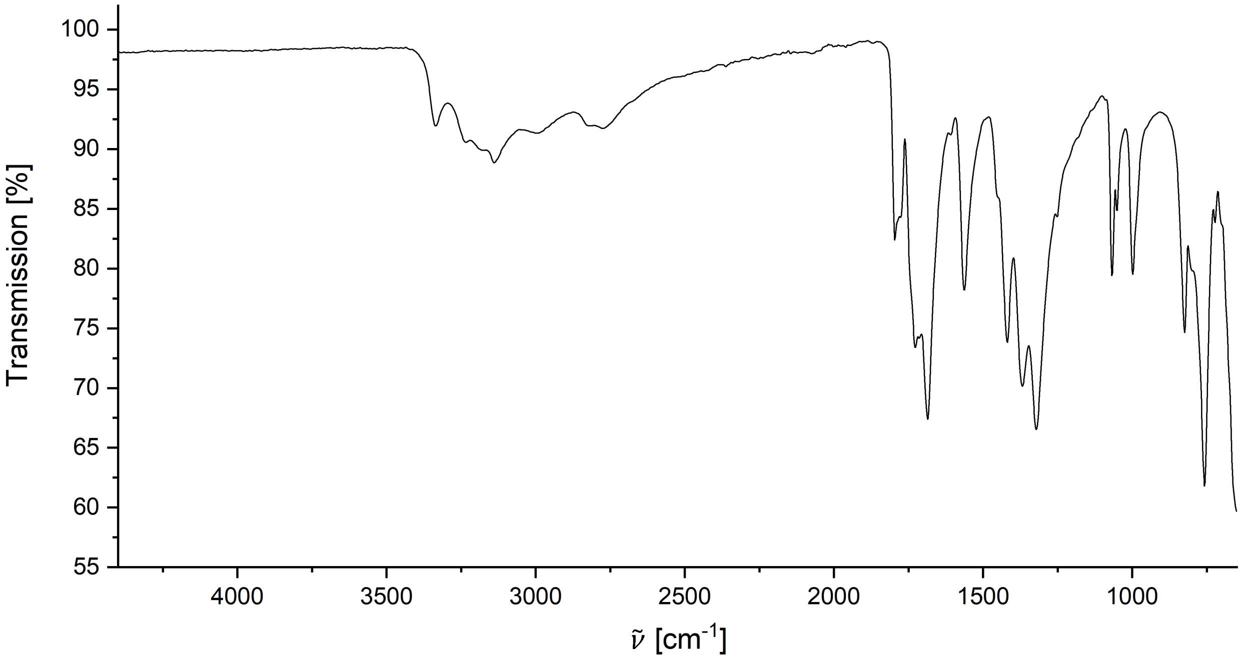

Figure S7. FTIR spectrum of ammelidium nitrate (**4**).

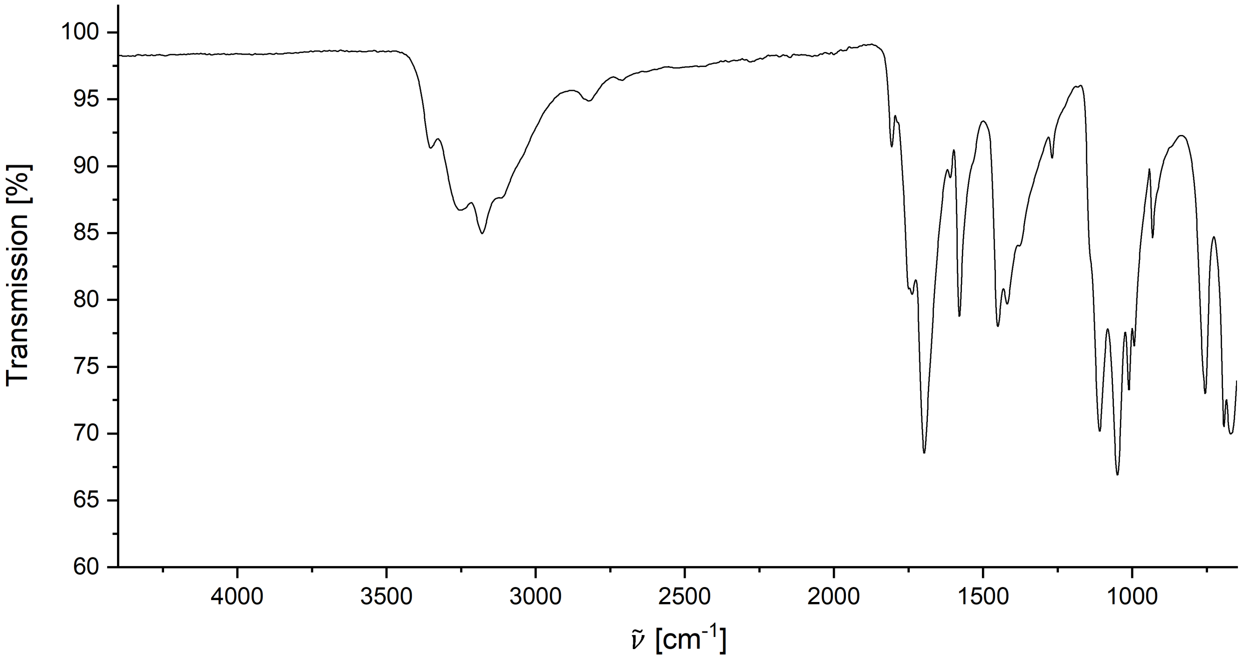

Figure S8. FTIR spectrum of ammelidium perchlorate (**5**).

SCXRD Data

Table S2. Selected crystallographic data of the three ammeline melanurate modifications (**1·2α**, **1·2β** and **1·2γ**).

Formula	α-$C_3H_5N_5O·C_3H_4N_4O_2$ (**1·2α**)	β-$C_3H_5N_5O·C_3H_4N_4O_2$ (**1·2β**)	γ-$C_3H_5N_5O·C_3H_4N_4O_2$ (**1·2γ**)
Molar mass [g/mol]	255.22	255.22	255.22
Crystal system	monoclinic	monoclinic	trigonal
Space group	*C*2/*m* (no. 12)	*C*2/*c* (no. 15)	$P3_121$ (no. 152)[a]
Lattice parameters [Å; °]	*a* = 14.726(4)<br>*b* = 9.570(2)<br>*c* = 3.5570(9)<br>*β* = 92.571(9)	*a* = 16.5876(14)<br>*b* = 9.5937(8)<br>*c* = 6.5959(6)<br>*β* = 104.985(3)	*a* = 9.5855(6)<br>*b* = 9.5855(6)<br>*c* = 9.4906(4)
Unit cell volume [Å^3]	500.8(2)	1013.95(15)	755.19(10)
Formula per unit cell	2	4	3
Calculated density [g/cm^3]	1.693	1.672	1.684
F(000)	264	528	396
Absorption coefficient [mm^{-1}]	0.139	0.137	0.138
Temperature [K]	173(2)	173(2)	173(2)
Radiation wavelength [Å]	0.71073 (Mo-K_α)	0.71073 (Mo-K_α)	0.71073 (Mo-K_α)
Diffractometer	Bruker D8 Venture	Bruker D8 Venture	Bruker D8 Venture
θ range [°]	$4.259 \leq \theta \leq 24.980$	$3.775 \leq \theta \leq 24.992$	$3.260 \leq \theta \leq 25.363$
Index ranges	$-17 \leq h \leq 17$<br>$-11 \leq k \leq 11$<br>$-3 \leq l \leq 4$	$-19 \leq h \leq 18$<br>$-11 \leq k \leq 11$<br>$-7 \leq l \leq 7$	$-11 \leq h \leq 11$<br>$-11 \leq k \leq 11$<br>$-11 \leq l \leq 11$
Total number of reflections	2367	5225	8287
Independent reflections [$I \geq 2\sigma(I)$ / all data]	410 / 466	664/ 894	753 / 923
R_σ / R_{int}	0.0296 / 0.0365	0.0302 / 0.0462	0.0183 / 0.0393
Parameters	51	91	85
Restraints	2	4	6
R-values [$I \geq 2\sigma(I)$]	R_1 = 0.0598; wR_2 = 0.1559	R_1 = 0.0439; wR_2 = 0.1072	R_1 = 0.0499; wR_2 = 0.1265
R-values (all data)	R_1 = 0.0672; wR_2 = 0.1607	R_1 = 0.0627; wR_2 = 0.1143	R_1 = 0.0604; wR_2 = 0.1358
Goodness of fit	1.111	1.037	1.157
Max. / min. residual electron density [e/Å^3]	0.533 / -0.348	0.395 / -0.186	0.702 / -0.330
CCDC	2486965	2486966	2486968

[a] The structure can equally be solved in space group $P3_221$ (no. 154).

Table S3. Selected crystallographic data for ammelide (**2**), ammelinium nitrate (**3**), ammelidium nitrate (**4**) and ammelidium perchlorate (**5**).

Formula	$C_3H_4N_4O_2$ (**2**)	$(C_3H_6N_5O)NO_3$ (**3**)	$(C_3H_5N_4O_2)NO_3$ (**4**)	$(C_3H_5N_4O_2)ClO_4$ (**5**)
Molar mass [g/mol]	128.10	190.14	191.12	228.56
Crystal system	monoclinic	monoclinic	monoclinic	monoclinic
Space group	$P2_1/c$ (no. 14)	$P2_1/c$ (no. 14)	$P2_1/n$ (no. 14)	$P2_1/n$ (no. 14)
Lattice parameters [Å; °]	*a* = 6.3345(12)<br>*b* = 9.7509(13)<br>*c* = 7.9355(14)<br>*β* = 98.535(9)	*a* = 9.2996(4)<br>*b* = 6.2638(3)<br>*c* = 13.4898(6)<br>*β* = 108.019(2)	*a* = 6.2224(4)<br>*b* = 4.6285(2)<br>*c* = 23.7842(15)<br>*β* = 94.006(2)	*a* = 7.6704(5)<br>*b* = 10.2444(6)<br>*c* = 10.2540(6)<br>*β* = 90.731(2)
Unit cell volume [Å^3]	484.72(14)	747.25(6)	683.32(7)	805.68(8)
Formula per unit cell	4	4	4	4
Calculated density [g/cm^3]	1.755	1.690	1.858	1.884
F(000)	264	392	392	464
Absorption coefficient [mm^{-1}]	0.149	0.153	0.497	0.491
Temperature [K]	173(2)	173(2)	173(2)	173(2)
Radiation wavelength [Å]	0.71073 (Mo-K$_\alpha$)	0.71073 (Mo-K$_\alpha$)	0.71073 (Mo-K$_\alpha$)	0.71073 (Mo-K$_\alpha$)
Diffractometer	Bruker D8 Venture	Bruker D8 Venture	Bruker D8 Venture	Bruker D8 Venture
θ range [°]	$3.252 \le \theta \le 24.993$	$3.176 \le \theta \le 27.497$	$3.334 \le \theta \le 26.388$	$3.297 \le \theta \le 27.490$
Index ranges	$-7 \le h \le 7$<br>$-11 \le k \le 11$<br>$-9 \le l \le 9$	$-12 \le h \le 12$<br>$-8 \le k \le 7$<br>$-17 \le l \le 17$	$-7 \le h \le 7$<br>$0 \le k \le 5$<br>$0 \le l \le 29$	$-9 \le h \le 9$<br>$-13 \le k \le 13$<br>$-13 \le l \le 13$
Total number of reflections	6870	12996	-[a]	17615
Independent reflections [$I \ge 2\sigma(I)$ / all data]	509 / 801	1373 / 1710	1381 / 1465	1768 / 1851
R_σ / R_{int}	0.0448 / 0.0929	0.0282 / 0.0462	0.0270 / -[a]	0.0183 / 0.0290
Parameters	82	142	139	148
Restraints	0	0	0	0
R-values [$I \ge 2\sigma(I)$]	R_1 = 0.0947; wR_2 = 0.2240	R_1 = 0.0458; wR_2 = 0.1003	R_1 = 0.0319; wR_2 = 0.0799	R_1 = 0.0335; wR_2 = 0.0879
R-values (all data)	R_1 = 0.1322; wR_2 = 0.2448	R_1 = 0.0622; wR_2 = 0.1060	R_1 = 0.0367; wR_2 = 0.0844	R_1 = 0.0354; wR_2 = 0.0895
Goodness of fit	1.019	1.082	1.161	1.141
Max. / min. residual electron density [e/Å^3]	0.967 / -0.287	0.188 / -0.219	0.202 / -0.221	0.404 / -0.428
CCDC	2486970	2486975	2486976	2486977

[a] The crystal was integrated and solved as a twin. The twin law corresponds to a real axis rotation of 180° about *a*.

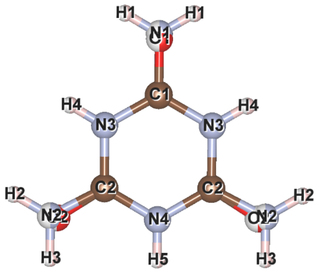

Figure S9. Twofold asymmetric unit of α-ammeline melanurate (**1·2α**) with all atoms labeled.

Table S4. Fractional atomic coordinates and isotropic atomic displacement parameters U_{iso} of all independent atoms of α-ammeline melanurate (**1·2α**).

Atom label	*x*	*y*	*z*	U_{iso} [Å^2]
O1	0.827(2)	0.500000	0.397(12)	0.0209(14)
O2	0.5890(19)	0.269(3)	0.851(9)	0.019(2)
N1	0.835(3)	0.500000	0.394(15)	0.0209(14)
N2	0.589(2)	0.253(3)	0.822(11)	0.019(2)
N3	0.71185(13)	0.3764(2)	0.6171(6)	0.0167(7)
N4	0.58718(18)	0.500000	0.8538(8)	0.0168(8)
C1	0.7530(2)	0.500000	0.5426(9)	0.0150(8)
C2	0.62818(17)	0.3772(2)	0.7707(7)	0.0162(7)
H1	0.861768	0.420366	0.342849	0.025
H2	0.617128	0.168305	0.767808	0.022
H3	0.532376	0.252819	0.926698	0.022
H4	0.738692	0.296806	0.567279	0.020
H5	0.534436	0.500001	0.960747	0.020

Table S5. Anisotropic atomic displacement parameters U_{ij} of all independent atoms of α-ammeline melanurate (**1·2α**).

Atom label	U_{11} [Å^2]	U_{22} [Å^2]	U_{33} [Å^2]	U_{23} [Å^2]	U_{13} [Å^2]	U_{12} [Å^2]
O1	0.019(4)	0.0141(14)	0.0307(18)	0.000	0.012(3)	0.000
O2	0.0185(11)	0.009(5)	0.030(4)	-0.006(3)	0.0113(19)	-0.001(3)
N1	0.019(4)	0.0141(14)	0.0307(18)	0.000	0.012(3)	0.000
N2	0.0185(11)	0.009(5)	0.030(4)	-0.006(3)	0.0113(19)	-0.001(3)
N3	0.0171(11)	0.0114(12)	0.0220(12)	-0.0023(8)	0.0060(9)	0.0021(8)
N4	0.0148(14)	0.0151(16)	0.0211(17)	0.000	0.0072(12)	0.000
C1	0.0146(16)	0.0145(17)	0.0161(17)	0.000	0.0031(13)	0.000
C2	0.0154(12)	0.0138(13)	0.0197(13)	0.0002(9)	0.0041(10)	0.0002(9)

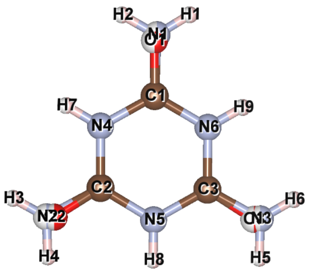

Figure S10. Asymmetric unit of β-ammeline melanurate (**1·2β**) with all atoms labeled.

Table S6. Fractional atomic coordinates and isotropic atomic displacement parameters U_{iso} of all independent atoms of β-ammeline melanurate (**1·2β**).

Atom label	*x*	*y*	*z*	U_{iso} [Å^2]
O1	0.0893(12)	-0.1598(19)	0.238(4)	0.0280(11)
O2	0.3210(11)	0.078(3)	0.249(4)	0.0306(14)
O3	0.0889(14)	0.308(2)	0.230(4)	0.0340(12)
N1	0.0815(16)	-0.168(2)	0.228(4)	0.0280(11)
N2	0.3286(13)	0.069(3)	0.240(5)	0.0306(14)
N3	0.0820(17)	0.314(2)	0.237(5)	0.0340(12)
N4	0.20638(10)	-0.05009(16)	0.2327(2)	0.0249(4)
N5	0.20614(10)	0.19574(16)	0.2375(2)	0.0272(5)
N6	0.08267(10)	0.07341(15)	0.2311(3)	0.0255(4)
C1	0.12435(12)	-0.04886(18)	0.2326(3)	0.0225(5)
C2	0.24721(12)	0.07242(18)	0.2392(3)	0.0247(5)
C3	0.12397(12)	0.19628(18)	0.2344(3)	0.0251(5)
H1	0.030952	-0.170863	0.229377	0.034
H2	0.109444	-0.246634	0.230049	0.034
H3	0.355811	-0.005399	0.243184	0.037
H4	0.353858	0.149755	0.247321	0.037
H5	0.108614	0.391079	0.237850	0.041
H6	0.030680	0.313379	0.236413	0.041
H7	0.232468	-0.129492	0.228548	0.030
H8	0.232916	0.275018	0.238428	0.033
H9	0.029306	0.073049	0.227912	0.031

Table S7. Anisotropic atomic displacement parameters U_{ij} of all independent atoms of β-ammeline melanurate (**1·2β**).

Atom label	U_{11} [Å^2]	U_{22} [Å^2]	U_{33} [Å^2]	U_{23} [Å^2]	U_{13} [Å^2]	U_{12} [Å^2]
O1	0.021(3)	0.0215(17)	0.0427(19)	-0.0013(15)	0.011(2)	-0.0015(17)
O2	0.021(3)	0.021(3)	0.0535(18)	-0.0010(16)	0.015(2)	0.0031(19)
O3	0.025(3)	0.0193(15)	0.0626(18)	-0.0029(13)	0.0201(18)	0.0023(15)
N1	0.021(3)	0.0215(17)	0.0427(19)	-0.0013(15)	0.011(2)	-0.0015(17)
N2	0.021(3)	0.021(3)	0.0535(18)	-0.0010(16)	0.015(2)	0.0031(19)
N3	0.025(3)	0.0193(15)	0.0626(18)	-0.0029(13)	0.0201(18)	0.0023(15)
N4	0.0234(9)	0.0213(9)	0.0310(10)	-0.0004(6)	0.0090(7)	0.0011(7)
N5	0.0242(9)	0.0221(10)	0.0356(10)	-0.0010(7)	0.0086(7)	0.0009(7)
N6	0.0226(8)	0.0232(9)	0.0325(9)	-0.0002(7)	0.0104(7)	0.0000(7)
C1	0.0258(10)	0.0220(11)	0.0199(10)	-0.0006(7)	0.0064(7)	-0.0022(8)
C2	0.0243(10)	0.0247(10)	0.0257(10)	0.0005(9)	0.0077(7)	-0.0017(9)
C3	0.0277(11)	0.0202(11)	0.0285(10)	-0.0016(8)	0.0089(8)	0.0002(8)

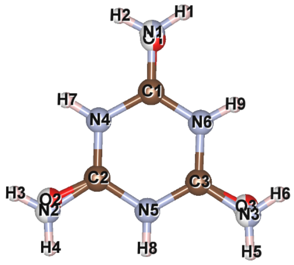

Figure S11. Asymmetric unit of γ-ammeline melanurate (**1·2γ**) with all atoms labeled.

Table S8. Fractional atomic coordinates and isotropic atomic displacement parameters U_{iso} of all independent atoms of γ-ammeline melanurate (**1·2γ**).

Atom label	*x*	*y*	*z*	U_{iso} [Å^2]
O1	0.7414(19)	0.923(5)	0.511(4)	0.0222(13)
O2	0.288(2)	0.4567(12)	0.510(2)	0.0167(17)
O3	0.283(3)	0.926(2)	0.5109(14)	0.0167(17)
N1	0.759(2)	0.929(7)	0.508(5)	0.0222(13)
N2	0.259(3)	0.4379(15)	0.512(3)	0.0167(17)
N3	0.261(4)	0.921(3)	0.5002(17)	0.0167(17)
N4	0.5133(7)	0.6828(7)	0.5122(3)	0.0180(9)
N5	0.2663(4)	0.6816(7)	0.5130(2)	0.0169(8)
N6	0.5132(7)	0.9282(3)	0.5094(3)	0.0178(7)
C1	0.5956(4)	0.8485(7)	0.5104(3)	0.0173(8)
C2	0.3524(8)	0.6018(4)	0.5122(3)	0.0182(8)
C3	0.3483(7)	0.8437(8)	0.5092(3)	0.0163(9)
H1	0.817407	1.032534	0.508235	0.027
H2	0.803451	0.870679	0.507668	0.027
H3	0.304632	0.379771	0.512619	0.020
H4	0.156135	0.392682	0.511343	0.020
H5	0.157680	0.876413	0.500441	0.020
H6	0.319973	1.023716	0.498204	0.020
H7	0.566679	0.630142	0.513382	0.022
H8	0.160359	0.627917	0.516012	0.020
H9	0.565172	1.034165	0.508817	0.021

Table S9. Anisotropic atomic displacement parameters U_{ij} of all independent atoms of γ-ammeline melanurate (**1·2γ**).

Atom label	U_{11} [Å^2]	U_{22} [Å^2]	U_{33} [Å^2]	U_{23} [Å^2]	U_{13} [Å^2]	U_{12} [Å^2]
O1	0.004(3)	0.013(3)	0.0459(19)	-0.003(3)	0.004(3)	0.001(4)
O2	0.004(5)	0.014(2)	0.0317(12)	0.001(2)	-0.003(2)	0.004(3)
O3	0.004(5)	0.014(2)	0.0317(12)	0.001(2)	-0.003(2)	0.004(3)
N1	0.004(3)	0.013(3)	0.0459(19)	-0.003(3)	0.004(3)	0.001(4)
N2	0.004(5)	0.014(2)	0.0317(12)	0.001(2)	-0.003(2)	0.004(3)
N3	0.004(5)	0.014(2)	0.0317(12)	0.001(2)	-0.003(2)	0.004(3)
N4	0.020(2)	0.0134(19)	0.0240(13)	0.002(2)	0.003(2)	0.0106(13)
N5	0.0161(18)	0.021(2)	0.0195(12)	-0.002(2)	-0.0004(10)	0.014(2)
N6	0.011(2)	0.0124(16)	0.0262(13)	0.0003(10)	-0.001(2)	0.0033(19)
C1	0.0142(17)	0.009(2)	0.0223(14)	0.002(2)	0.0016(12)	0.001(2)
C2	0.036(3)	0.0167(18)	0.0145(12)	-0.0005(11)	-0.0001(19)	0.022(3)
C3	0.008(2)	0.021(3)	0.0186(13)	-0.0029(19)	-0.0027(19)	0.0068(15)

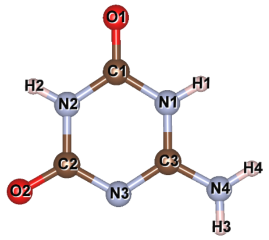

Figure S12. Asymmetric unit of ammelide (**2**) with all atoms labeled.

Table S10. Fractional atomic coordinates and isotropic atomic displacement parameters U_{iso} of all independent atoms of ammelide (**2**).

Atom label	*x*	*y*	*z*	U_{iso} [Å^2]
O1	0.8277(5)	0.3548(4)	0.0858(4)	0.0247(10)
O2	0.8217(5)	-0.1107(3)	0.0853(4)	0.0176(9)
N1	0.5805(6)	0.2398(4)	0.2121(5)	0.0159(11)
N2	0.8329(6)	0.1212(4)	0.0923(5)	0.0188(10)
N3	0.5768(6)	-0.0022(4)	0.2174(5)	0.0194(12)
N4	0.3327(6)	0.1239(4)	0.3411(5)	0.0201(10)
C1	0.7542(8)	0.2448(5)	0.1290(6)	0.0197(13)
C2	0.7458(7)	-0.0020(5)	0.1304(6)	0.0173(12)
C3	0.4985(6)	0.1209(5)	0.2565(5)	0.0123(10)
H1	0.520617	0.317106	0.237356	0.019
H2	0.946804	0.119593	0.040923	0.023
H3	0.276666	0.046820	0.371288	0.024
H4	0.278916	0.203081	0.366874	0.024

Table S11. Anisotropic atomic displacement parameters U_{ij} of all independent atoms of ammelide (**2**).

Atom label	U_{11} [Å^2]	U_{22} [Å^2]	U_{33} [Å^2]	U_{23} [Å^2]	U_{13} [Å^2]	U_{12} [Å^2]
O1	0.0225(19)	0.023(2)	0.0307(19)	-0.0033(16)	0.0124(15)	-0.0046(15)
O2	0.0198(17)	0.0066(18)	0.0292(18)	-0.0036(15)	0.0129(14)	-0.0013(14)
N1	0.018(2)	0.009(2)	0.022(2)	0.0020(15)	0.0084(19)	0.0038(15)
N2	0.017(2)	0.012(2)	0.030(2)	0.0057(19)	0.0113(16)	0.0042(18)
N3	0.020(2)	0.017(3)	0.024(2)	0.0015(17)	0.0115(18)	-0.0007(17)
N4	0.022(2)	0.011(2)	0.030(2)	-0.0039(19)	0.0129(16)	-0.0003(19)
C1	0.019(3)	0.017(3)	0.023(3)	0.001(2)	0.004(2)	0.000(2)
C2	0.017(3)	0.013(3)	0.022(2)	0.0006(19)	0.005(2)	-0.0014(19)
C3	0.015(2)	0.009(2)	0.014(2)	-0.0004(17)	0.0035(17)	0.0017(17)

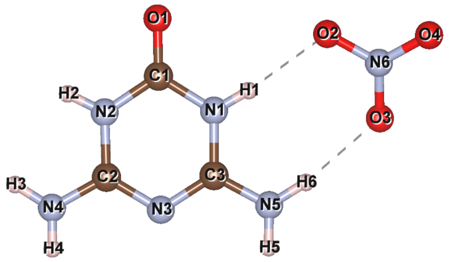

Figure S13. Asymmetric unit of ammelinium nitrate (**3**) with all atoms labeled.

Table S12. Fractional atomic coordinates and isotropic atomic displacement parameters U_{iso} of all independent atoms of ammelinium nitrate (**3**).

Atom label	*x*	*y*	*z*	U_{iso} [Å^2]
O1	0.70343(15)	0.0839(2)	0.31750(9)	0.0263(3)
O2	0.30776(14)	0.1369(2)	0.28986(10)	0.0261(3)
O3	0.12838(15)	0.1236(2)	0.14209(10)	0.0303(4)
O4	0.07542(15)	0.1661(3)	0.28655(10)	0.0331(4)
N1	0.51511(17)	0.1694(3)	0.16948(11)	0.0204(3)
N2	0.76332(18)	0.1617(2)	0.17082(11)	0.0202(3)
N3	0.57240(17)	0.2520(2)	0.01463(11)	0.0201(3)
N4	0.82239(19)	0.2506(3)	0.02313(13)	0.0239(4)
N5	0.32788(17)	0.2494(3)	0.01667(13)	0.0212(4)
N6	0.17002(17)	0.1427(2)	0.23903(12)	0.0211(3)
C1	0.6638(2)	0.1351(3)	0.22619(13)	0.0194(4)
C2	0.7174(2)	0.2222(3)	0.06852(13)	0.0181(4)
C3	0.4731(2)	0.2251(3)	0.06639(13)	0.0185(4)
H1	0.449(2)	0.159(4)	0.2004(16)	0.024(5)
H2	0.860(3)	0.154(4)	0.2030(17)	0.027(6)
H3	0.921(3)	0.219(4)	0.0601(17)	0.031(6)
H4	0.795(2)	0.287(3)	-0.0418(18)	0.027(6)
H5	0.302(3)	0.291(4)	-0.050(2)	0.042(7)
H6	0.259(3)	0.232(4)	0.0511(18)	0.037(7)

Table S13. Anisotropic atomic displacement parameters U_{ij} of all independent atoms of ammelinium nitrate (**3**).

Atom label	U_{11} [Å^2]	U_{22} [Å^2]	U_{33} [Å^2]	U_{23} [Å^2]	U_{13} [Å^2]	U_{12} [Å^2]
O1	0.0288(7)	0.0342(8)	0.0150(6)	0.0018(5)	0.0058(5)	-0.0009(6)
O2	0.0156(6)	0.0353(8)	0.0243(7)	0.0043(6)	0.0016(5)	-0.0001(6)
O3	0.0236(7)	0.0465(9)	0.0192(7)	-0.0027(6)	0.0045(5)	-0.0020(6)
O4	0.0209(7)	0.0546(10)	0.0259(7)	-0.0017(7)	0.0101(6)	0.0006(7)
N1	0.0179(8)	0.0271(8)	0.0181(7)	-0.0005(6)	0.0085(6)	-0.0004(6)
N2	0.0162(8)	0.0264(8)	0.0162(7)	0.0008(6)	0.0025(6)	0.0011(7)
N3	0.0204(8)	0.0222(8)	0.0170(7)	0.0006(6)	0.0049(6)	0.0001(6)
N4	0.0186(9)	0.0356(9)	0.0174(8)	0.0030(7)	0.0054(7)	0.0012(7)
N5	0.0181(8)	0.0262(8)	0.0188(8)	0.0005(6)	0.0050(6)	0.0003(7)
N6	0.0193(8)	0.0214(8)	0.0220(8)	0.0008(6)	0.0053(6)	-0.0008(6)
C1	0.0230(9)	0.0167(8)	0.0180(9)	-0.0025(7)	0.0058(7)	-0.0008(7)
C2	0.0201(9)	0.0173(8)	0.0165(8)	-0.0019(7)	0.0049(7)	0.0002(7)
C3	0.0205(9)	0.0153(8)	0.0191(8)	-0.0023(7)	0.0051(7)	0.0001(7)

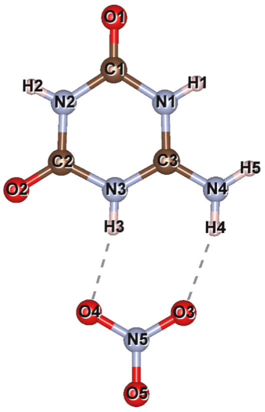

Figure S14. Asymmetric unit of ammelidium nitrate (**4**) with all atoms labeled.

Table S14. Fractional atomic coordinates and isotropic atomic displacement parameters U_{iso} of all independent atoms of ammelidium nitrate (**4**).

Atom label	*x*	*y*	*z*	U_{iso} [Å^2]
O1	0.2356(2)	1.1287(3)	0.29884(6)	0.0245(3)
O2	0.8211(2)	0.5384(3)	0.30332(6)	0.0240(3)
O3	0.6607(2)	1.0031(3)	0.46589(6)	0.0260(4)
O4	0.8903(2)	1.0516(3)	0.40216(5)	0.0213(3)
O5	0.9341(2)	0.7182(3)	0.46586(6)	0.0233(3)
N1	0.2793(2)	0.7842(3)	0.36664(6)	0.0157(3)
N2	0.5293(3)	0.8370(4)	0.29931(7)	0.0173(3)
N3	0.5832(2)	0.4960(3)	0.37039(6)	0.0149(3)
N4	0.3313(3)	0.4398(4)	0.43658(7)	0.0187(4)
N5	0.8292(2)	0.9246(3)	0.44510(6)	0.0145(3)
C1	0.3395(3)	0.9311(4)	0.31915(7)	0.0158(4)
C2	0.6555(3)	0.6191(4)	0.32221(7)	0.0155(4)
C3	0.3956(3)	0.5699(4)	0.39226(7)	0.0139(4)
H1	0.163(4)	0.838(5)	0.3800(10)	0.025(6)
H2	0.571(4)	0.904(6)	0.2698(11)	0.034(7)
H3	0.666(4)	0.364(6)	0.3854(9)	0.022(6)
H4	0.418(4)	0.306(6)	0.4529(10)	0.029(6)
H5	0.204(4)	0.495(5)	0.4494(9)	0.021(6)

Table S15. Anisotropic atomic displacement parameters U_{ij} of all independent atoms of ammelidium nitrate (**4**).

Atom label	U_{11} [Å^2]	U_{22} [Å^2]	U_{33} [Å^2]	U_{23} [Å^2]	U_{13} [Å^2]	U_{12} [Å^2]
O1	0.0259(7)	0.0238(7)	0.0236(7)	0.0069(6)	0.0008(6)	0.0086(6)
O2	0.0194(7)	0.0337(8)	0.0199(7)	-0.0006(6)	0.0077(5)	0.0083(6)
O3	0.0159(7)	0.0300(8)	0.0334(8)	0.0060(6)	0.0109(6)	0.0100(6)
O4	0.0229(7)	0.0244(7)	0.0174(6)	0.0062(6)	0.0063(5)	0.0037(6)
O5	0.0212(7)	0.0231(7)	0.0259(7)	0.0076(6)	0.0038(6)	0.0113(6)
N1	0.0114(7)	0.0179(8)	0.0182(8)	0.0006(6)	0.0034(6)	0.0032(6)
N2	0.0175(8)	0.0218(8)	0.0129(7)	0.0035(6)	0.0036(6)	0.0025(6)
N3	0.0137(7)	0.0151(8)	0.0158(7)	0.0006(6)	0.0017(6)	0.0040(6)
N4	0.0167(8)	0.0204(9)	0.0198(8)	0.0047(7)	0.0055(6)	0.0035(7)
N5	0.0129(7)	0.0160(8)	0.0144(7)	-0.0003(6)	0.0004(6)	0.0015(6)
C1	0.0167(8)	0.0179(9)	0.0124(8)	-0.0021(7)	-0.0011(7)	0.0000(8)
C2	0.0153(8)	0.0191(9)	0.0121(8)	-0.0038(7)	0.0008(6)	-0.0001(7)
C3	0.0122(8)	0.0147(9)	0.0148(8)	-0.0030(7)	0.0003(6)	-0.0005(7)

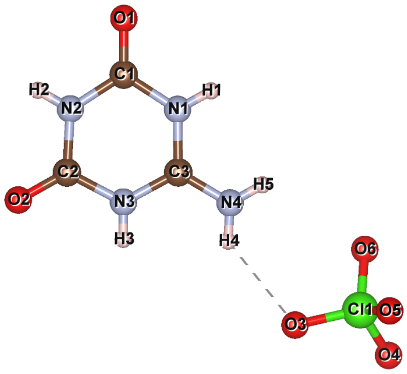

Figure S15. Asymmetric unit of ammelidium perchlorate (**5**) with all atoms labeled.

Table S16. Fractional atomic coordinates and isotropic atomic displacement parameters U_{iso} of all independent atoms of ammelidium perchlorate (**5**).

Atom label	*x*	*y*	*z*	U_{iso} [Å^2]
Cl1	0.38679(6)	0.47919(4)	0.69957(5)	0.01819(14)
O1	0.47964(18)	0.15224(14)	0.45579(13)	0.0191(3)
O2	0.5320(2)	0.13170(17)	0.89501(14)	0.0280(4)
O3	0.2686(2)	0.43776(17)	0.80117(16)	0.0314(4)
O4	0.5627(2)	0.45474(17)	0.7399(2)	0.0361(4)
O5	0.3623(2)	0.61701(14)	0.67951(17)	0.0293(4)
O6	0.3447(2)	0.40968(18)	0.58236(17)	0.0386(4)
N1	0.7225(2)	0.23550(16)	0.55448(15)	0.0172(3)
N2	0.5100(2)	0.13309(17)	0.67483(16)	0.0190(3)
N3	0.7549(2)	0.21702(16)	0.77824(15)	0.0153(3)
N4	0.9651(2)	0.32146(18)	0.65951(18)	0.0215(4)
C1	0.5636(2)	0.17216(18)	0.55434(17)	0.0158(4)
C2	0.5937(2)	0.15918(19)	0.79084(18)	0.0173(4)
C3	0.8180(2)	0.25939(18)	0.66418(17)	0.0154(4)
H1	0.754(3)	0.270(3)	0.485(3)	0.028(7)
H2	0.417(4)	0.097(3)	0.679(3)	0.026(7)
H3	0.810(4)	0.231(3)	0.844(3)	0.028(7)
H4	1.024(3)	0.326(3)	0.728(3)	0.029(7)
H5	1.003(4)	0.344(3)	0.591(3)	0.031(7)

Table S17. Anisotropic atomic displacement parameters U_{ij} of all independent atoms of ammelidium perchlorate (**5**).

Atom label	U_{11} [Å^2]	U_{22} [Å^2]	U_{33} [Å^2]	U_{23} [Å^2]	U_{13} [Å^2]	U_{12} [Å^2]
Cl1	0.0143(2)	0.0156(2)	0.0247(2)	0.00040(17)	0.00163(16)	0.00018(15)
O1	0.0219(7)	0.0205(7)	0.0147(6)	-0.0012(5)	-0.0052(5)	-0.0009(5)
O2	0.0240(8)	0.0459(10)	0.0141(7)	0.0042(6)	0.0001(5)	-0.0076(7)
O3	0.0249(8)	0.0410(9)	0.0284(8)	0.0091(7)	0.0032(6)	-0.0070(7)
O4	0.0162(8)	0.0288(8)	0.0631(12)	0.0052(8)	-0.0049(7)	0.0050(6)
O5	0.0295(8)	0.0168(7)	0.0412(9)	0.0037(6)	-0.0103(7)	0.0007(6)
O6	0.0426(10)	0.0389(10)	0.0346(9)	-0.0165(8)	0.0044(7)	-0.0057(8)
N1	0.0180(8)	0.0225(8)	0.0109(7)	0.0011(6)	0.0003(6)	-0.0036(6)
N2	0.0159(8)	0.0256(9)	0.0155(8)	0.0019(6)	-0.0020(6)	-0.0075(7)
N3	0.0151(7)	0.0191(8)	0.0115(7)	0.0007(6)	-0.0037(6)	-0.0005(6)
N4	0.0180(8)	0.0291(9)	0.0174(8)	0.0033(7)	-0.0018(7)	-0.0070(7)
C1	0.0185(9)	0.0146(8)	0.0143(8)	-0.0003(7)	-0.0010(6)	0.0016(7)
C2	0.0167(9)	0.0206(9)	0.0145(8)	0.0020(7)	-0.0013(7)	-0.0001(7)
C3	0.0158(8)	0.0148(8)	0.0156(8)	-0.0009(7)	0.0001(6)	0.0025(7)

PXRD Data

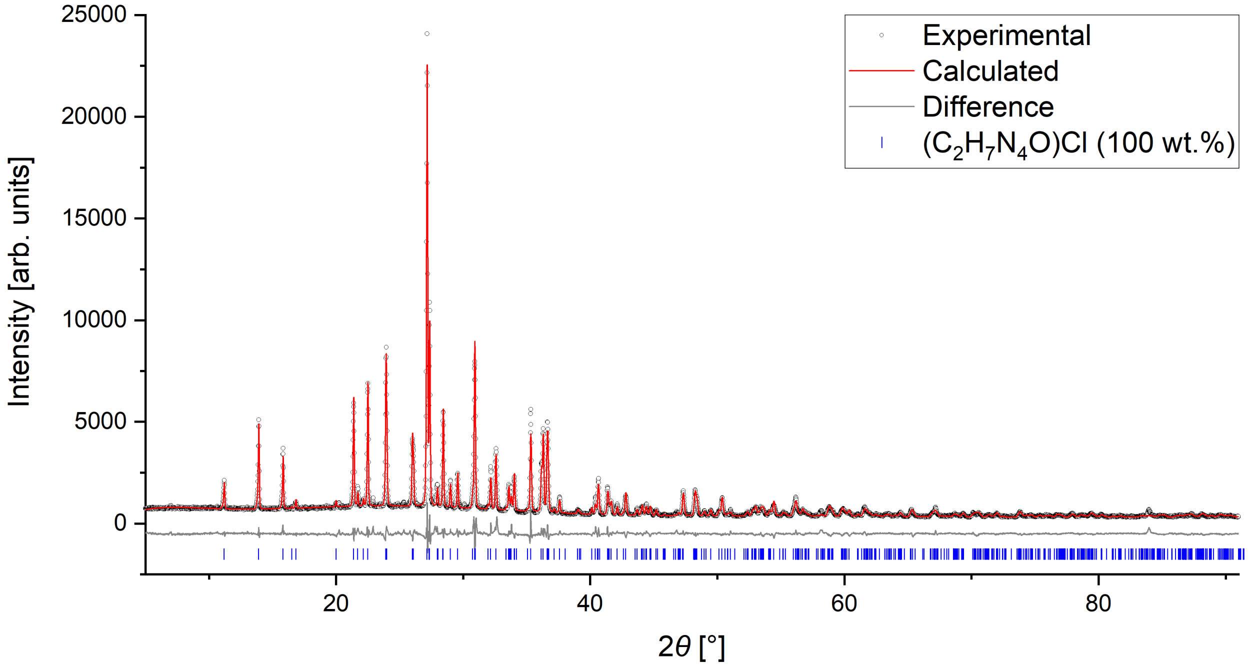

Figure S16. Quantitative phase analysis of the product obtained from the hydrolysis of dicyandiamide in boiling aqueous HCl (2 M) via Rietveld refinement of its PXRD pattern. The resulting difference plot (grey line) is depicted below the experimental (black circles) and calculated (red line) PXRD pattern. Blue lines mark the positions of the Bragg reflections of guanylurea hydrochloride.

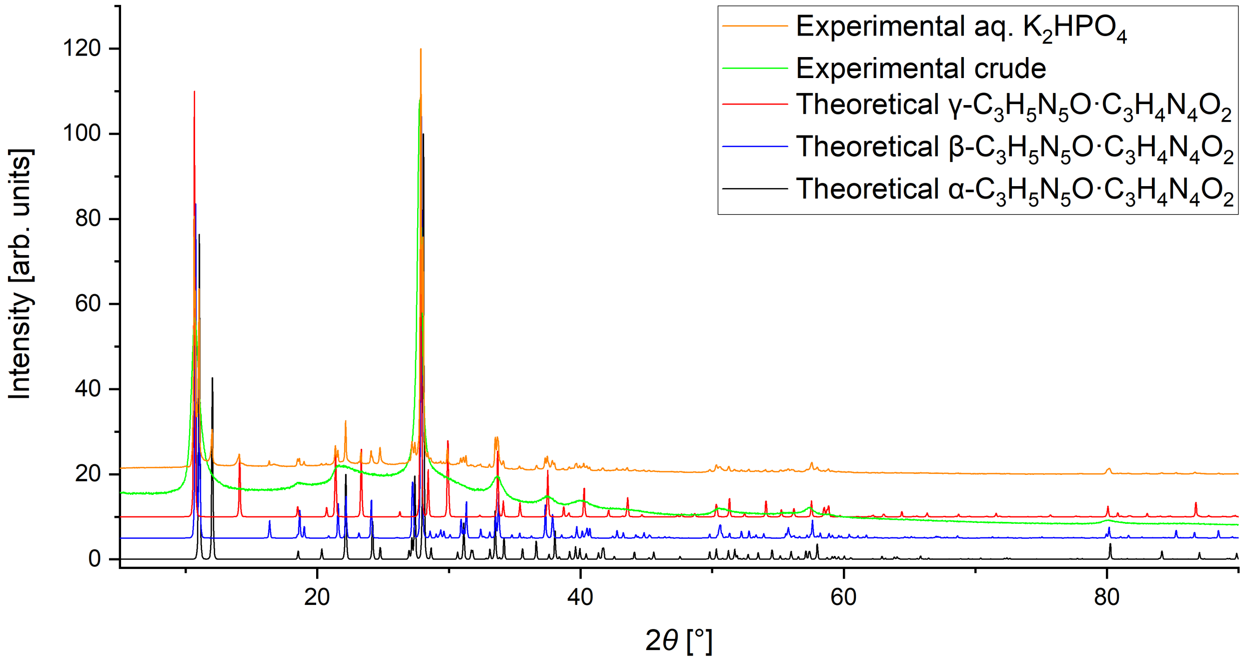

Figure S17. Experimental PXRD patterns of the product obtained from the reaction between guanylurea hydrochloride and KOCN after washing with boiling H_2O and drying at 105 °C (green) and of the product obtained from the recrystallization of a 1:1 molar mixture between ammeline (**1**) and ammelide (**2**) from aqueous K_2HPO_4 (0.4 M) at 150 °C inside of an autoclave (orange) in comparison to the theoretical PXRD patterns of α-ammeline melanurate (**1·2α**) (black), β-ammeline melanurate (**1·2β**) (blue) and γ-ammeline melanurate (**1·2γ**) (red).

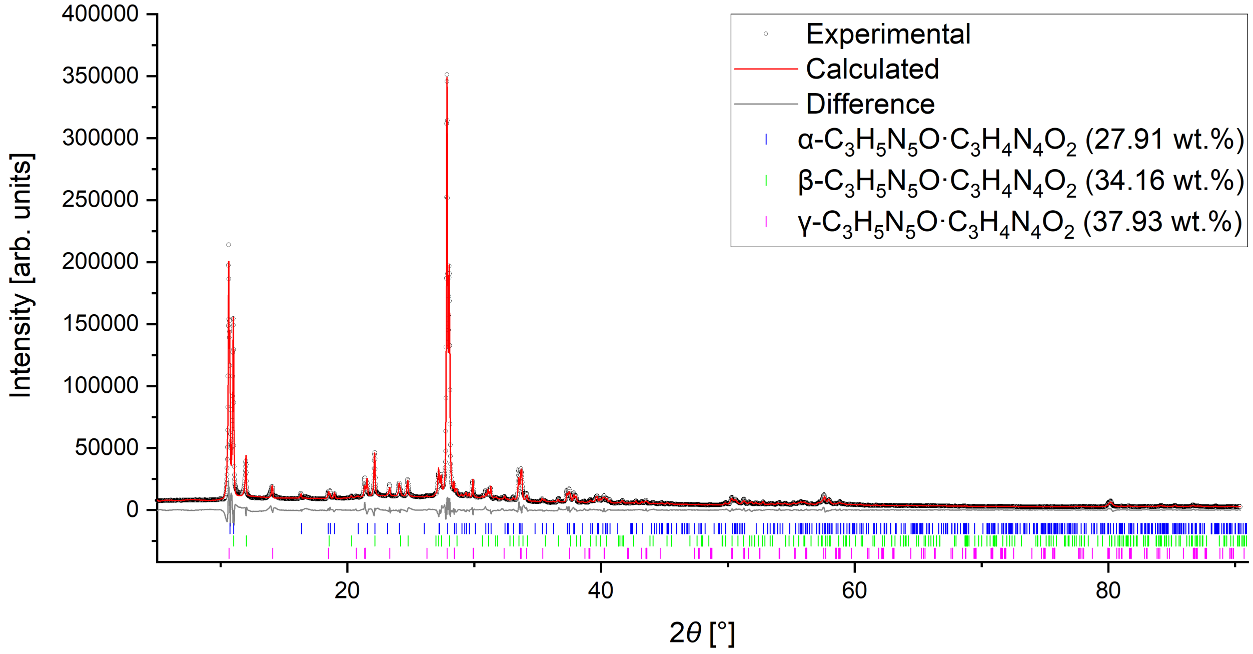

Figure S18. Quantitative phase analysis of the product obtained from the recrystallization of a 1:1 molar mixture between ammeline (**1**) and ammelide (**2**) from aqueous K_2HPO_4 (0.4 M) at 150 °C inside of an autoclave via Rietveld refinement of its PXRD pattern. The resulting difference plot (grey line) is depicted below the experimental (black circles) and calculated (red line) PXRD pattern. Blue, green and pink lines mark the positions of the Bragg reflections of the α-modification (**1·2α**), β-modification (**1·2β**) and γ-modification (**1·2γ**) of ammeline melanurate, respectively.

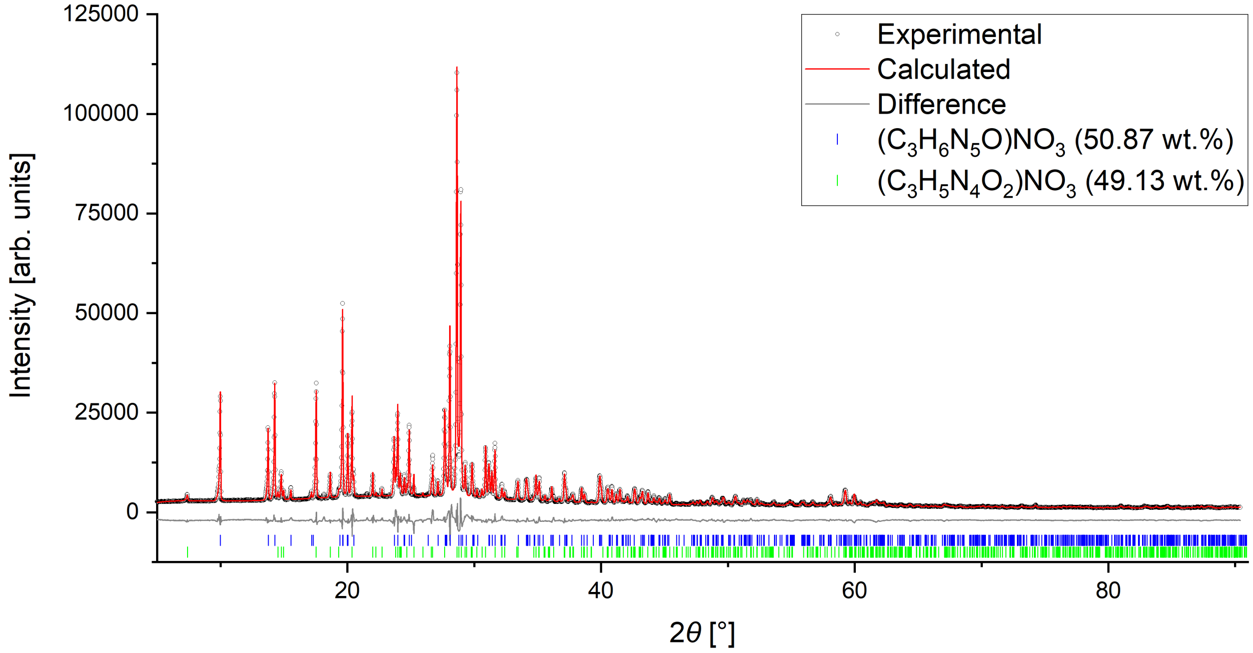

Figure S19. Quantitative phase analysis of the product obtained from the recrystallization of a 1:1 molar mixture between ammeline (**1**) and ammelide (**2**) from aqueous K_2HPO_4 (0.4 M) at 150 °C inside of an autoclave after dissolution in aqueous HNO_3 (2 M) via Rietveld refinement of its PXRD pattern. The resulting difference plot (grey line) is depicted below the experimental (black circles) and calculated (red line) PXRD pattern. Blue and green lines mark the positions of the Bragg reflections ammelinium nitrate (**3**) and ammelidium nitrate (**4**), respectively.

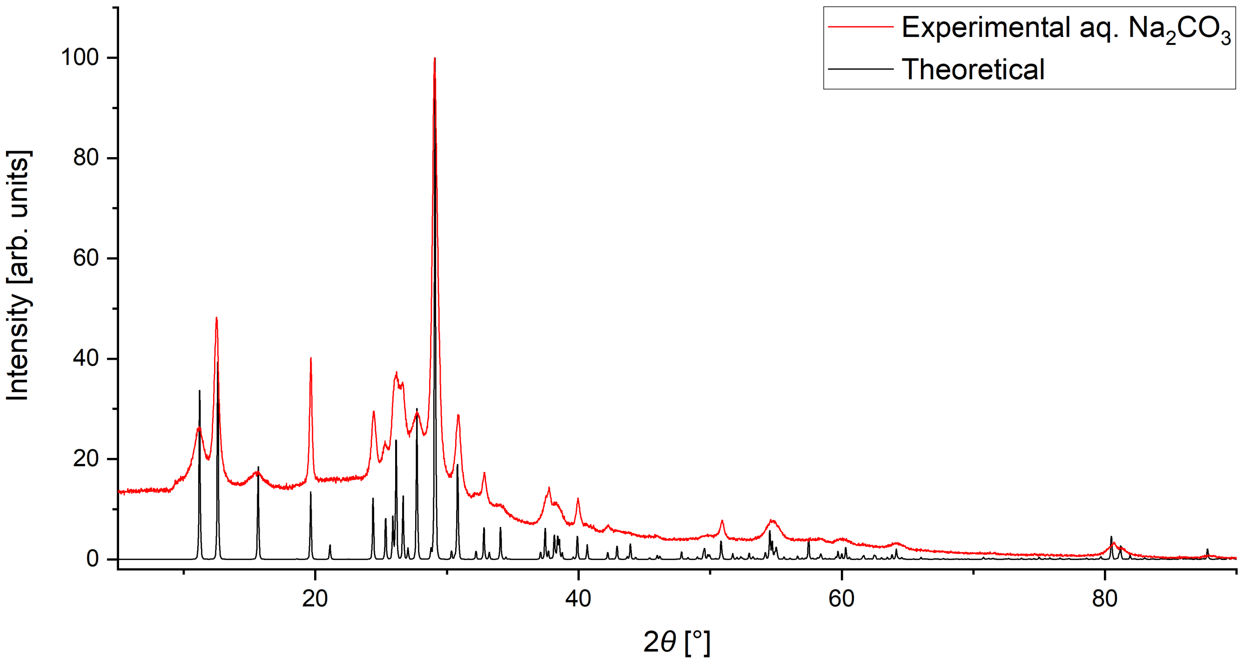

Figure S20. Experimental PXRD pattern of ammeline (**1**) obtained from the neutralized filtrate of a recrystallization of ammeline melanurate (**1·2**) in aqueous H_3PO_4 (0.2 M), followed by twofold recrystallization in aqueous Na_2CO_3 (0.2 M) (red) in comparison to the theoretical PXRD pattern (black).

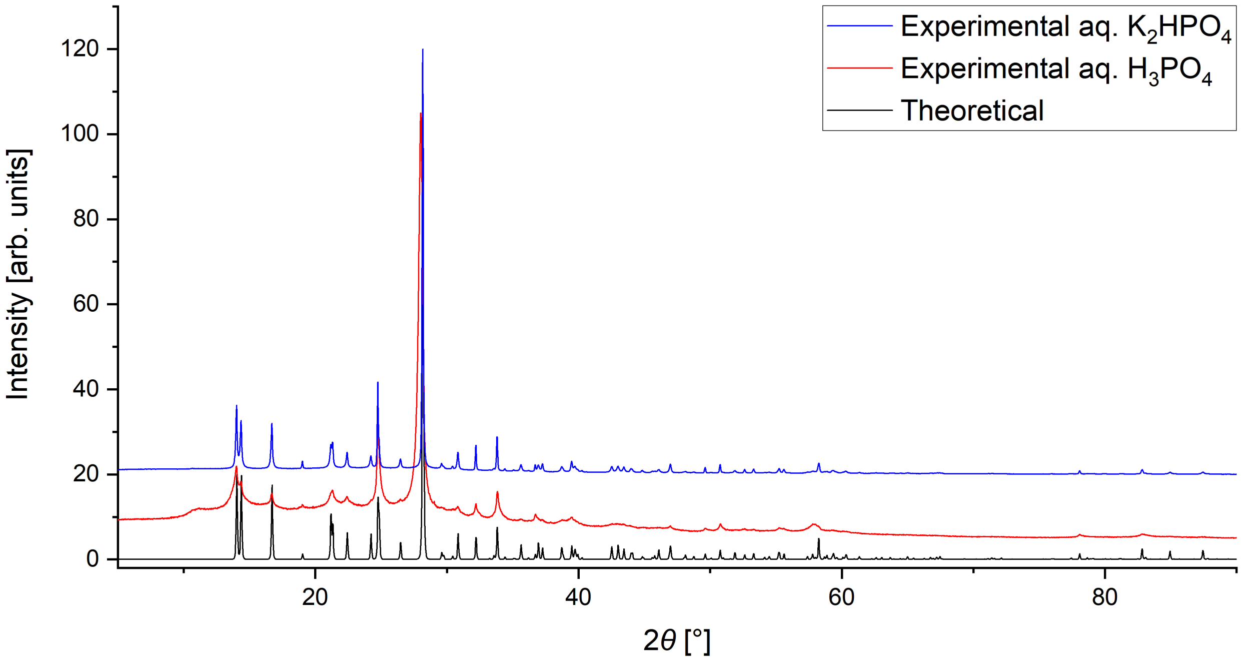

Figure S21. Experimental PXRD patterns of ammelide (**2**) obtained from two consecutive recrystallizations of ammeline melanurate (**1·2**) in aqueous H_3PO_4 (0.2 M) (red) and of the same after an additional recrystallization from aqueous K_2HPO_4 (0.4 M) inside of an autoclave (blue) in comparison to the theoretical PXRD pattern (black).

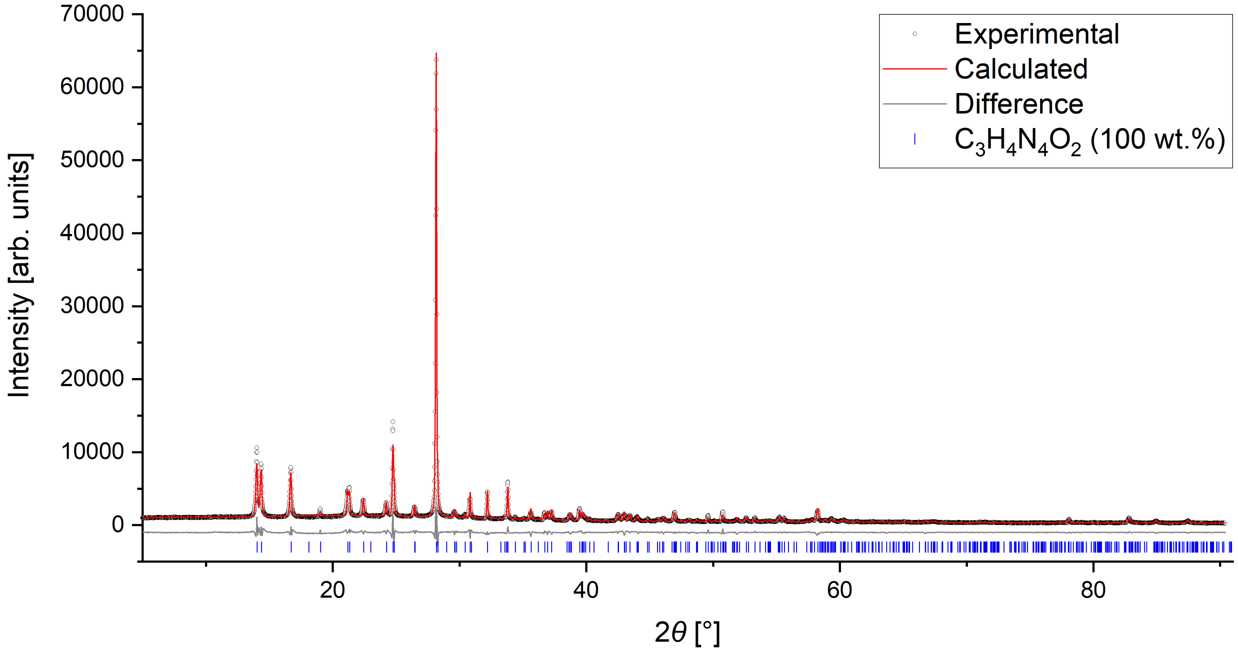

Figure S22. Quantitative phase analysis of the product obtained from two consecutive recrystallizations of ammeline melanurate (**1·2**) in aqueous H_3PO_4 (0.2 M), followed by a recrystallization from aqueous K_2HPO_4 (0.4 M) inside of an autoclave via Rietveld refinement of its PXRD pattern. The resulting difference plot (grey line) is depicted below the experimental (black circles) and calculated (red line) PXRD pattern. Blue lines mark the positions of the Bragg reflections of ammelide (**2**).

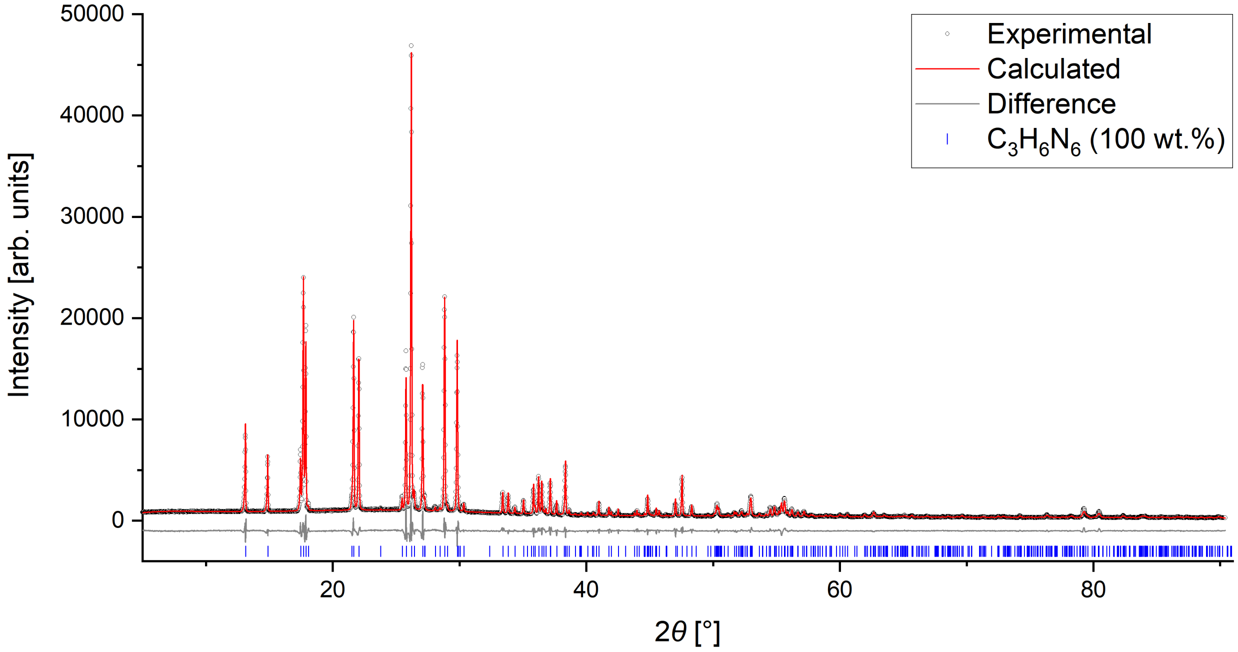

Figure S23. Quantitative phase analysis of the commercially purchased melamine via Rietveld refinement of its PXRD pattern. The resulting difference plot (grey line) is depicted below the experimental (black circles) and calculated (red line) PXRD pattern. Blue lines mark the positions of the Bragg reflections of melamine.

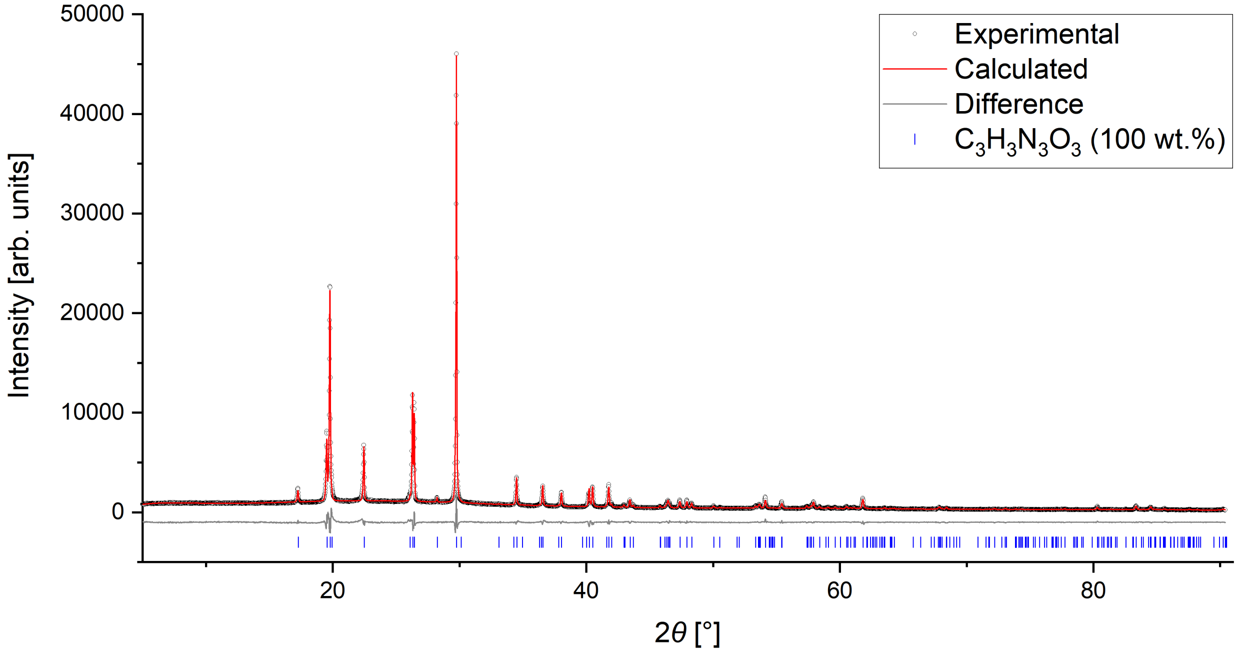

Figure S24. Quantitative phase analysis of the commercially purchased cyanuric acid via Rietveld refinement of its PXRD pattern. The resulting difference plot (grey line) is depicted below the experimental (black circles) and calculated (red line) PXRD pattern. Blue lines mark the positions of the Bragg reflections of cyanuric acid.

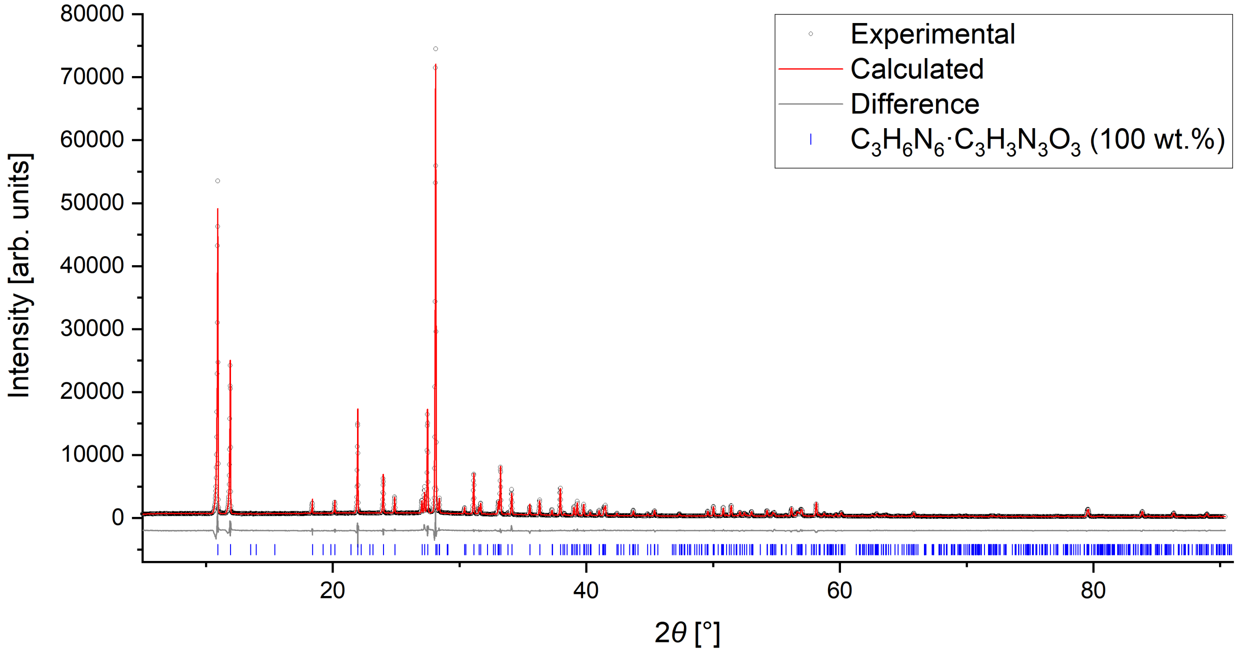

Figure S25. Quantitative phase analysis of the product obtained from the reaction between melamine and cyanuric acid at 150 °C in H_2O inside of an autoclave via Rietveld refinement of its PXRD pattern. The resulting difference plot (grey line) is depicted below the experimental (black circles) and calculated (red line) PXRD pattern. Blue lines mark the positions of the Bragg reflections of melamine cyanurate.

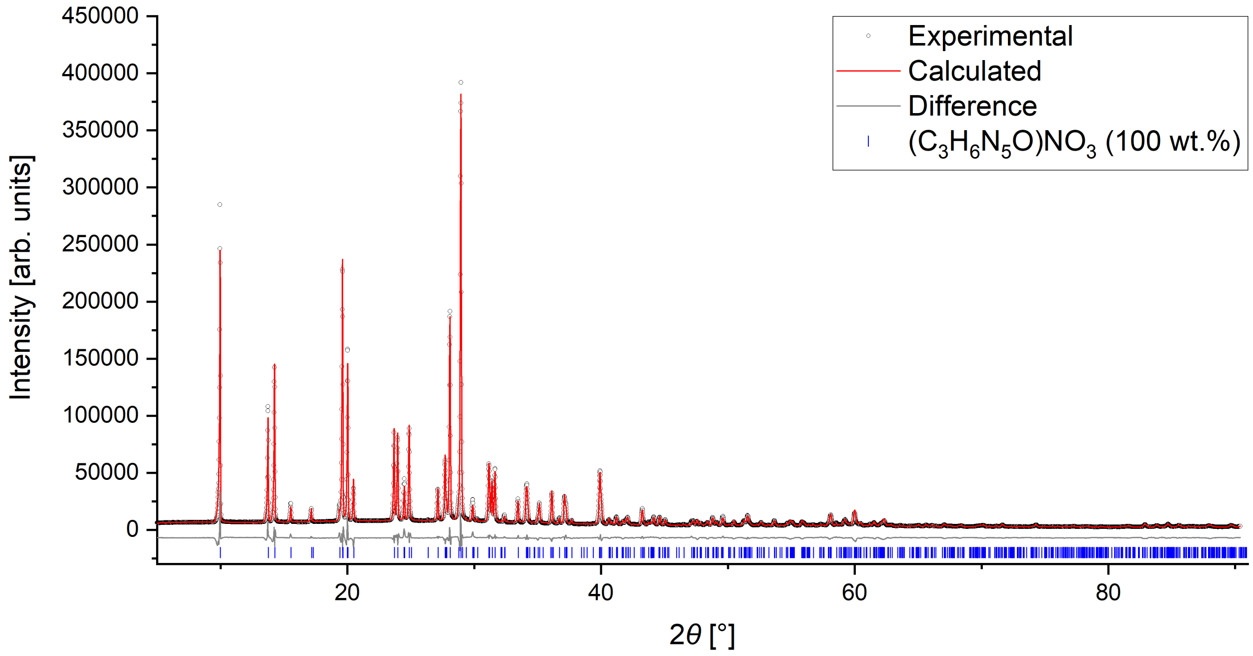

Figure S26. Quantitative phase analysis of the product obtained from the reaction between ammeline (**1**) and aqueous HNO_3 (2 M) via Rietveld refinement of its PXRD pattern. The resulting difference plot (grey line) is depicted below the experimental (black circles) and calculated (red line) PXRD pattern. Blue lines mark the positions of the Bragg reflections of ammelinium nitrate (**3**).

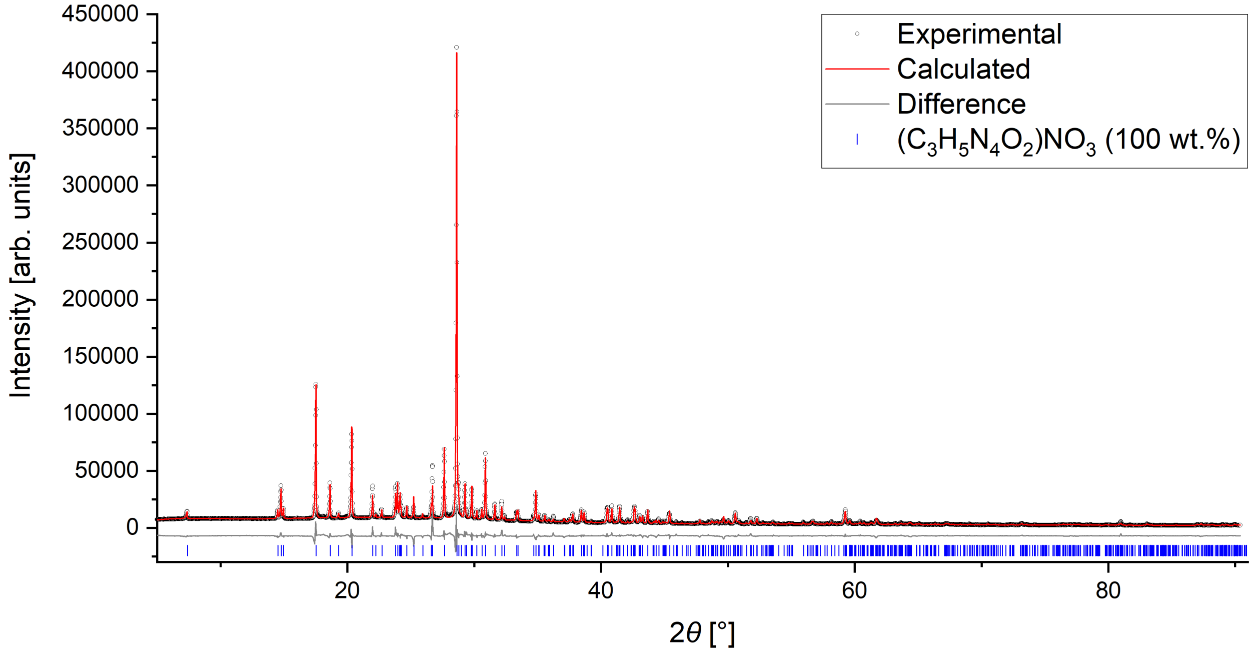

Figure S27. Quantitative phase analysis of the product obtained the reaction between ammelide (**2**) and aqueous HNO_3 (2 M) via Rietveld refinement of its PXRD pattern. The resulting difference plot (grey line) is depicted below the experimental (black circles) and calculated (red line) PXRD pattern. Blue lines mark the positions of the Bragg reflections of ammelidium nitrate (**4**).

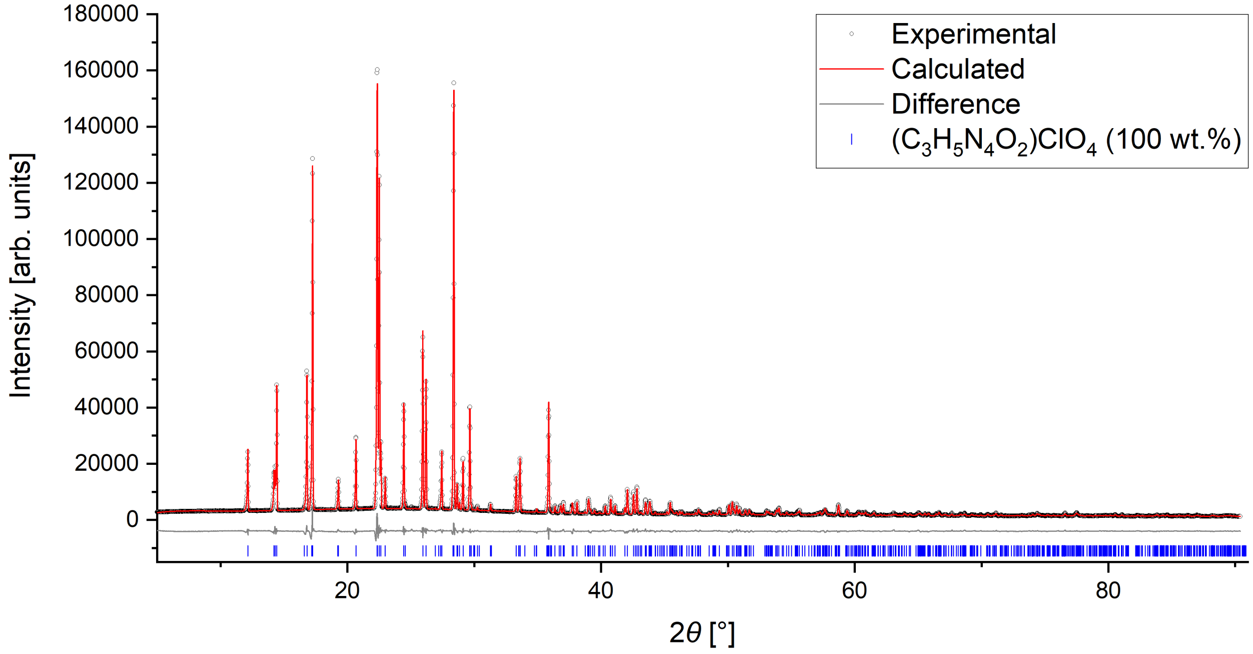

Figure S28. Quantitative phase analysis of the product obtained the reaction between ammelide (**2**) and aqueous $HClO_4$ (2 M) via Rietveld refinement of its PXRD pattern. The resulting difference plot (grey line) is depicted below the experimental (black circles) and calculated (red line) PXRD pattern. Blue lines mark the positions of the Bragg reflections of ammelidium perchlorate (**5**).

Table S18. Determined lattice parameters, cell volumes *V* and densities *ρ* of the different compounds at 20 °C by Rietveld refinement.

Phase[a]	*a* [Å]	*b* [Å]	*c* [Å]	*β* [°]	*V* [Å³]	Space group	*ρ* [g/cm³]
α-Ammeline melanurate (**1·2α**)	14.7207(11)	9.5615(9)	3.59468(19)	92.238(6)	505.57(6)	*C*2/*m* (no. 12)	1.676
β-Ammeline melanurate (**1·2β**)	16.5874(10)	9.5924(6)	6.6191(3)	105.021(7)	1017.20(10)	*C*2/*c* (no. 15)	1.666
γ-Ammeline melanurate (**1·2γ**)	9.5799(5)	9.5799(5)	6.6191(3)	-	763.13(8)	$P3_121$ (no. 152)	1.666
Ammeline (**1**)[b,18]	3.6817(3)	9.5282(8)	14.1433(12)	96.957(4)	492.50(7)	$P2_1/n$ (no. 14)	1.714
Ammelide (**2**)	6.36502(16)	9.7841(3)	7.9957(3)	97.8206(15)	493.31(2)	$P2_1/c$ (no. 14)	1.725
Ammelinium nitrate (**3**)	9.31486(11)	6.34693(7)	13.5359(2)	108.1087(11)	760.614(18)	$P2_1/c$ (no. 14)	1.660
Ammelidium nitrate (**4**)	6.21293(9)	4.67853(8)	23.9975(4)	93.6047(10)	696.16(2)	$P2_1/n$ (no. 14)	1.823
Ammelidium perchlorate (**5**)	7.73795(5)	10.31778(7)	10.27016(9)	90.9515(6)	819.840(10)	$P2_1/n$ (no. 14)	1.852
Guanylurea hydrochloride[19]	8.30077(13)	10.73087(19)	6.89608(11)	107.8363(14)	584.741(17)	$P2_1/c$ (no. 14)	1.574
Melamine[20]	7.27920(9)	7.47811(12)	10.33255(14)	108.4756(8)	533.459(13)	$P2_1/n$ (no. 14)	1.570
Cyanuric acid[21]	7.90913(16)	6.73654(13)	9.07702(19)	90.6870(8)	483.591(17)	*C*2/*c* (no. 15)	1.773
Melamine cyanurate[22]	14.83919(14)	9.63632(10)	7.15476(7)	92.2761(7)	1022.288(17)	*I*2/*m* (no. 12)	1.658

[a] The references cited refer to the structure models employed for the Rietveld refinements. [b] In this case, the cell parameters were adopted directly from the cited reference, as the same PXRD measurement device was used here as for the other compounds.

DTA and TGA Curves

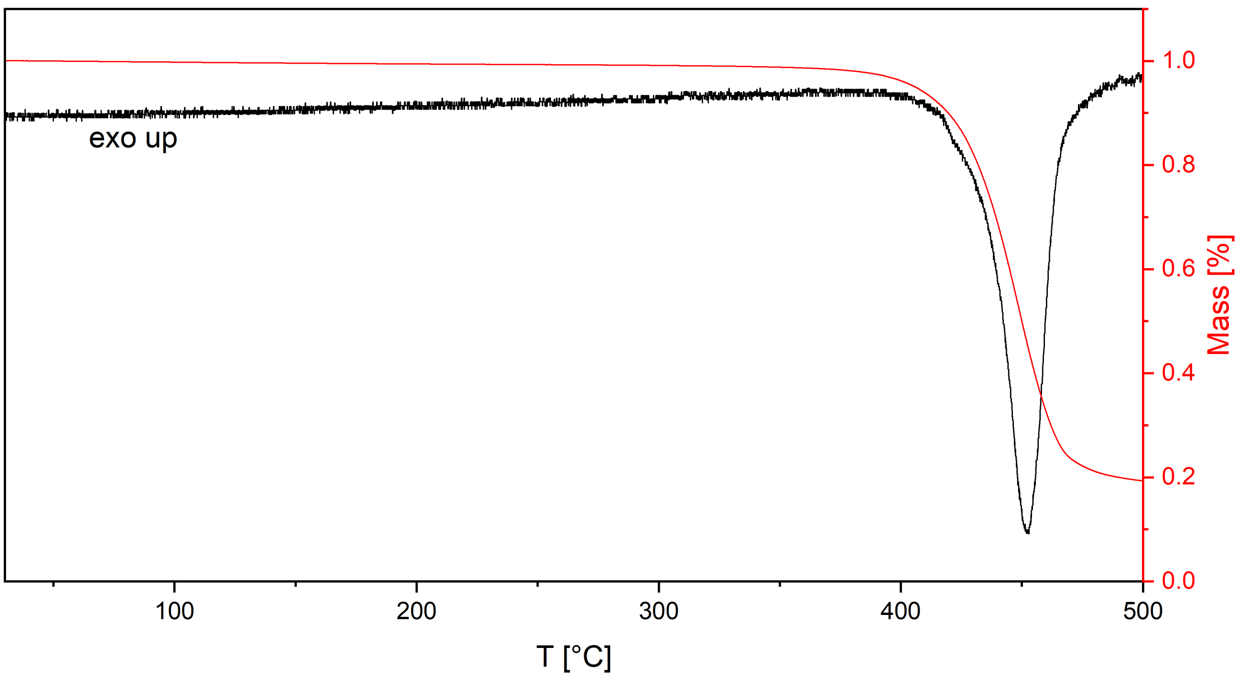

Figure S29. DTA (black) and TGA (red) curves of ammeline melanurate (**1·2**).

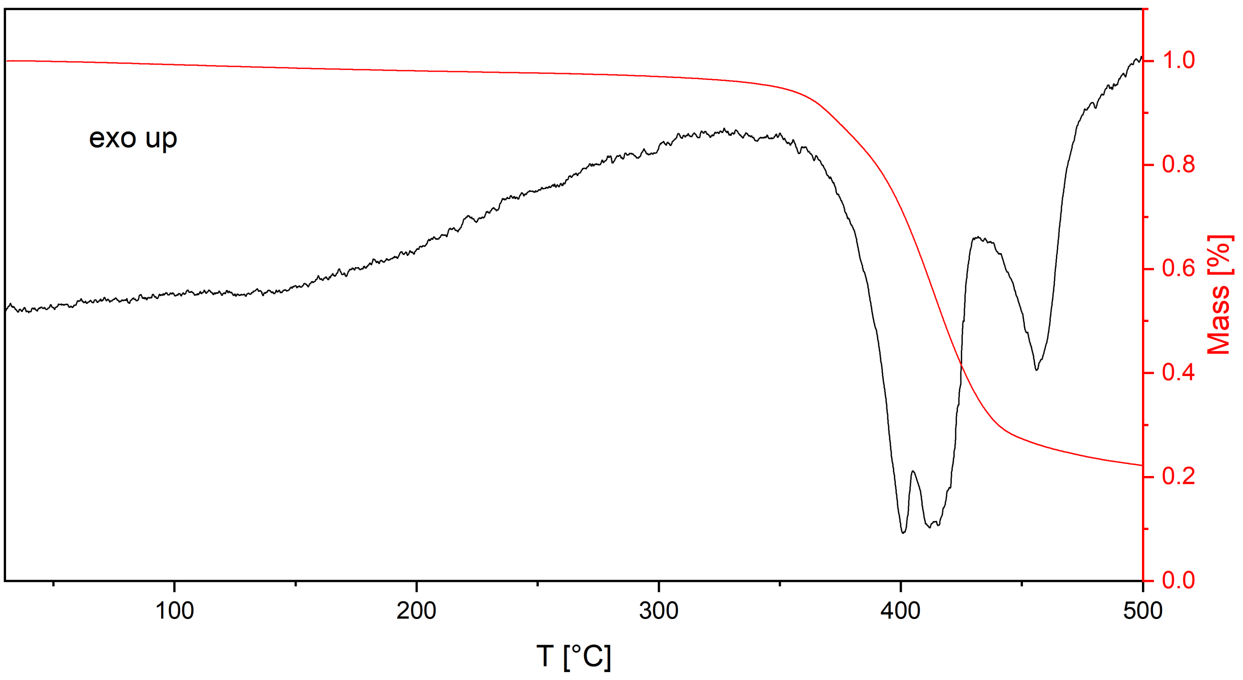

Figure S30. DTA (black) and TGA (red) curves of ammelide (**2**).

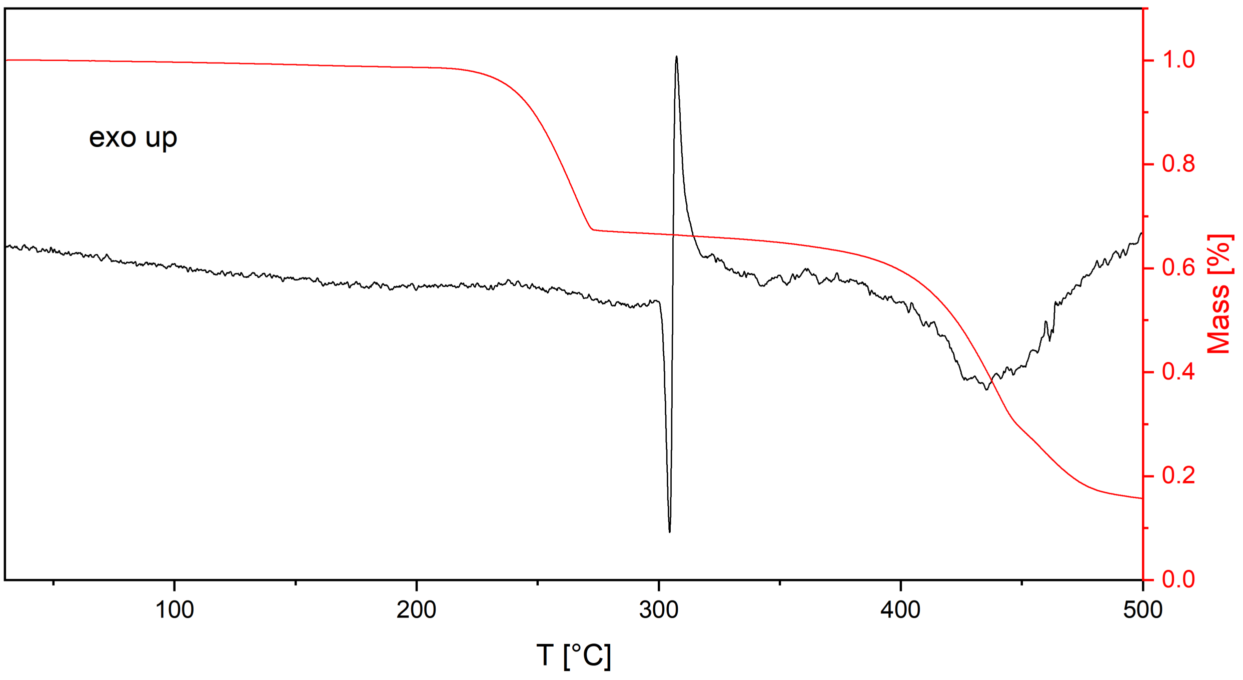

Figure S31. DTA (black) and TGA (red) curves of ammelinium nitrate (**3**).

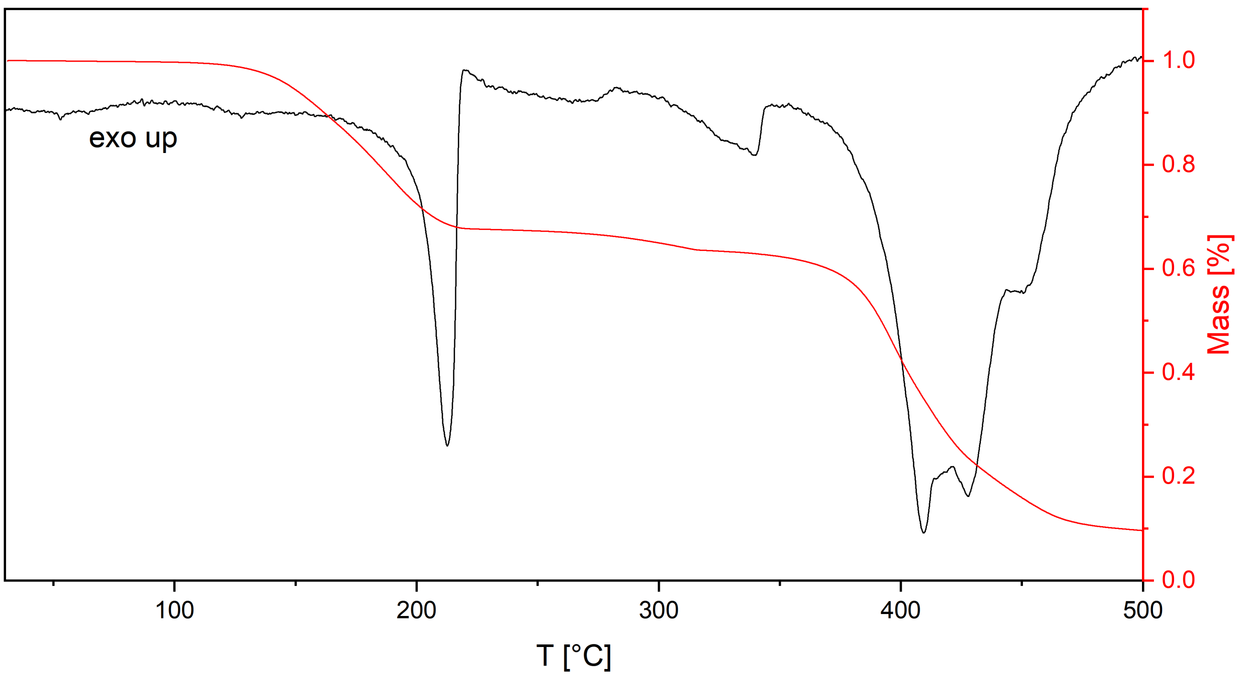

Figure S32. DTA (black) and TGA (red) curves of ammelidium nitrate (**4**).

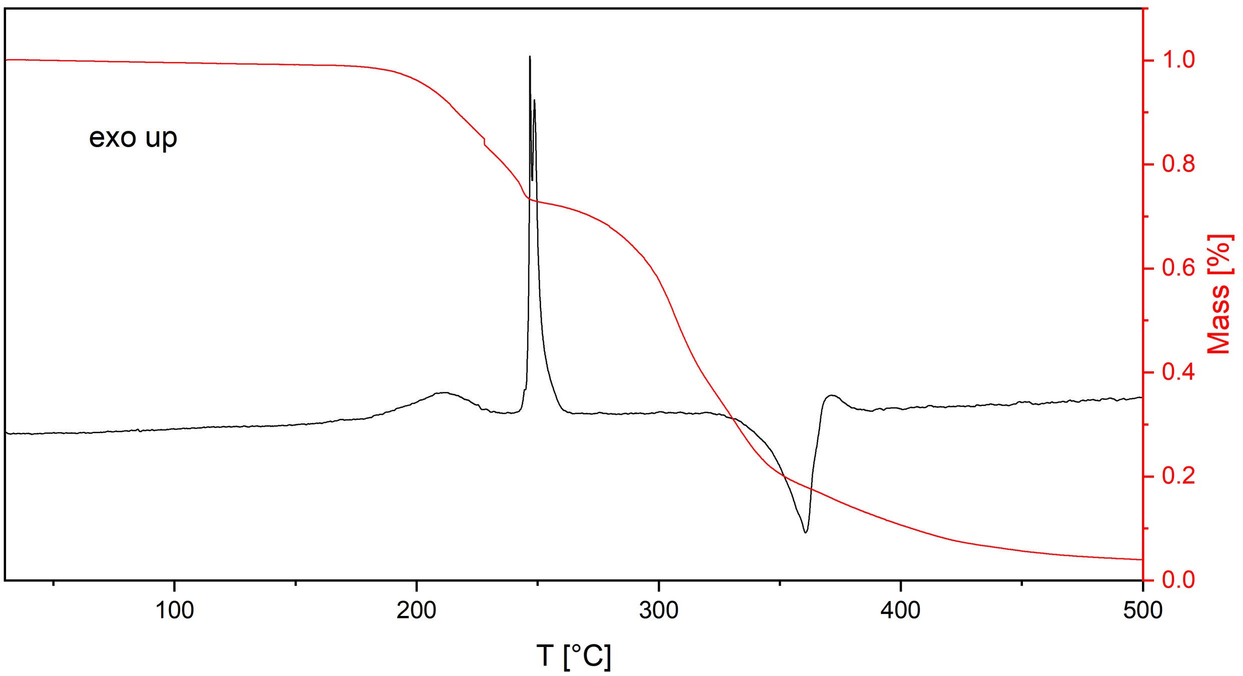

Figure S33. DTA (black) and TGA (red) curves of ammelidium perchlorate (**5**).

Computational Results

Table S19. Calculated VASP ground state energies (U_0) per sum formula of each stacking option of ammeline melanurate (**1·2**) (Figure S34) and their relative difference to the stacking with identical orientation of the layers (ΔU_0).

Stacking option	U_0 [eV]	ΔU_0 [J/mol]
(1)	−182.7205002442	0
(2)	−182.7200734692	+41
(3)	−182.7205165483	−2
(4)	−182.7296888533	−887
(5)	−182.7299336733	−910
(6)	−182.7299895983	−916

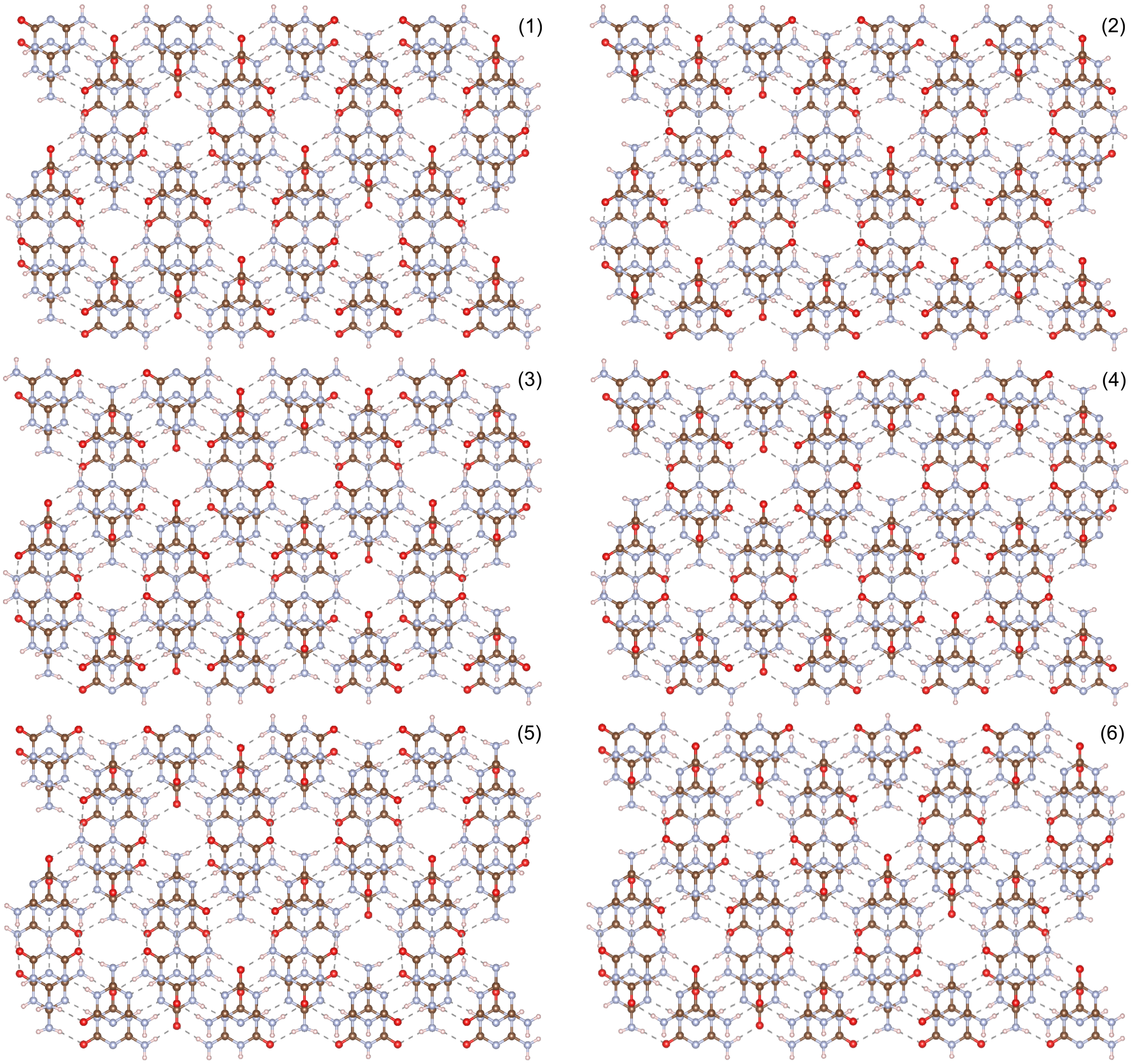

Figure S34. Depiction of the different stacking options of ammeline melanurate (**1·2**) employed during the calculations.

Table S20. Calculated VASP ground state energies (U_0) per sum formula of each stacking option of melamine cyanurate (Figure S35) and their relative difference to the stacking with identical orientation of the layers (ΔU_0).

Stacking option	U_0 [eV]	ΔU_0 [J/mol]
(1)	−180.6481878550	0
(2)	−180.7317877925	−8066

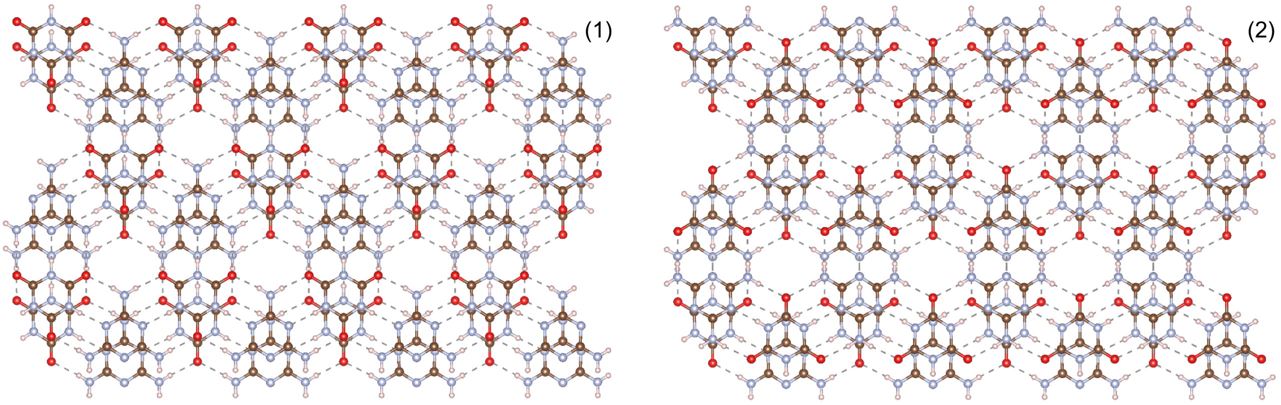

Figure S35. Depiction of the different stacking options of melamine cyanurate employed during the calculations.

Table S21. CBS-QB3 electronic enthalpies ($H°$) and gas phase enthalpies of formation ($\Delta_f H°_{(g)}$) according to ATcT Ver. 1.130 of the different atom sorts A.

Atom sort (A)	Symbol	$H°$(A) [a.u.]	$\Delta_f H°_{(g)}$(A)[12] [kJ/mol]
Hydrogen	H	−0.497457	+217.998
Carbon	C	−37.783015	+716.881
Nitrogen	N	−54.518173	+472.440
Oxygen	O	−74.985259	+249.229
Chlorine	Cl	−459.681232	+121.302

Table S22. Calculated CBS-QB3 electronic enthalpies ($H°$) and gas phase enthalpies of formation ($\Delta_f H°_{(g)}$) of the individual molecules M.

Molecule (M)	Molecular formula	$H°$(M) [a.u.]	$\Delta_f H°_{(g)}$(M) [kJ/mol]
Ammelinium	$C_3H_6N_5O^+$	−466.106451	+461.5
Ammelidium	$C_3H_5N_4O_2^+$	−485.903483	+315.0
Nitrate	NO_3^-	−280.061575	−322.7
Perchlorate	ClO_4^-	−760.151782	−272.0

Table S23. Calculated VASP ground state energies (U_0) per sum formula F in the crystal and of the individual molecular cations M^+ and anions M^- in isolation.

Compound	Sum formula	U_0(F) [eV]	U_0(M^+) [eV]	U_0(M^-) [eV]
**3**	$(C_3H_6N_5O)NO_3$	−121.8920835	−92.22234035	−23.12186050
**4**	$(C_3H_5N_4O_2)NO_3$	−117.1888292	−87.61341499	−23.11644973
**5**	$(C_3H_5N_4O_2)ClO_4$	−110.9386164	−84.21983362	−19.51815901

Table S24. Results of the computations for compounds **3**–**5**.

Compound	Sum formula	ΔU_L[a] [kJ/mol]	Δn_L[b]	ΔH_L[c] [kJ/mol]	$\Delta_f H°_{(s)}$[d] [kJ/mol]	$\Delta_f n$[e]	$\Delta_f U°_{(s)}$[f] [kJ/mol]
**3**	$(C_3H_6N_5O)NO_3$	−631.7	−2	−636.7	−497.9	−8.0	−478.1
**4**	$(C_3H_5N_4O_2)NO_3$	−623.2	−2	−628.2	−635.8	−7.5	−617.2
**5**	$(C_3H_5N_4O_2)ClO_4$	−694.8	−2	−699.7	−656.7	−8.0	−637.9

[a] Lattice energy. [b] Change of moles of gaseous components during crystallization from the gaseous state. [c] Lattice enthalpy. [d] Solid-state enthalpy of formation. [e] Change of moles of gaseous components when formed from elements in their standard state. [f] Solid-state energy of formation.

References

[1] *Origin 2019b*, OriginLab Corporation, Northampton (USA), **2019**.
[2] *ChemDraw 20*, PerkinElmer, Walton (USA), **2021**.
[3] *APEX3*, Bruker AXS, Karlsruhe (Germany), **2018**.
[4] G. M. Sheldrick, *Acta Crystallogr. Sect. A* **2008**, *64*, 112–122.
[5] K. Momma, F. Izumi, *J. Appl. Crystallogr.* **2008**, *41*, 653–658.
[6] *WinXPow 3.0.2.1*, STOE & Cie. GmbH, Darmstadt (Germany), **2011**.
[7] H. Rietveld, *J. Appl. Crystallogr.* **1969**, *2*, 65–71.
[8] *TOPAS 6*, Bruker AXS, Karlsruhe (Germany), **2016**.
[9] a) G. Kresse, J. Hafner, *Phys. Rev. B* **1993**, *47,* 558–561; b) G. Kresse, J. Hafner, *Phys. Rev. B* **1994**, *47,* 14251–14269; c) G. Kresse, J. Furthmüller, *Comput. Mat. Sci.* **1996,** *6*, 15–50*; d)* G. Kresse, J. Furthmüller, *Phys. Rev. B* **1996**, *54,* 11169–11186.
[10] a) P. E. Blöchl, *Phys. Rev. B* **1994**, *50*, 17953–17979; b) G. Kresse, D. Joubert, *Phys. Rev. B* **1999**, *59,* 1758–1175.
[11] J. P. Perdew, K. Burke, M. Ernzerhof, *Phys. Rev. Lett.* **1996**, *77*, 3865–3868.
[12] S. Grimme, S. Ehrlich, L. Goerigk, *J. Comput. Chem.* **2011**, *32*, 1456–1465.
[13] P. Pulay, *Chem. Phys. Lett.* **1980**, *73*, 393–398.
[14] M. J. Frisch, G. W. Trucks, H. B. Schlegel, G. E. Scuseria, M. A. Robb, J. R. Cheeseman, G. Scalmani, V. Barone, G. A. Petersson, H. Nakatsuji, X. Li, M. Caricato, A. V. Marenich, J. Bloino, B. G. Janesko, R. Gomperts, B. Mennucci, H. P. Hratchian, J. V. Ortiz, A. F. Izmaylov, J. L. Sonnenberg, D. Williams-Young, F. Ding, F. Lipparini, F. Egidi, J. Goings, B. Peng, A. Petrone, T. Henderson, D. Ranasinghe, V. G. Zakrzewski, J. Gao, N. Rega, G. Zheng, W. Liang, M. Hada, M. Ehara, K. Toyota, R. Fukuda, J. Hasegawa, M. Ishida, T. Nakajima, Y. Honda, O. Kitao, H. Nakai, T. Vreven, K. Throssell, J. A. Montgomery, Jr., J. E. Peralta, F. Ogliaro, M. J. Bearpark, J. J. Heyd, E. N. Brothers, K. N. Kudin, V. N. Staroverov, T. A. Keith, R. Kobayashi, J. Normand, K. Raghavachari, A. P. Rendell, J. C. Burant, S. S. Iyengar, J. Tomasi, M. Cossi, J. M. Millam, M. Klene, C. Adamo, R. Cammi, J. W. Ochterski, R. L. Martin, K. Morokuma, O. Farkas, J. B. Foresman, D. J. Fox, *Gaussian 16, Revision C.02*, Gaussian, Inc., Wallingford CT, **2019**.
[15] a) J. A. Montgomery Jr., M. J. Frisch, J. W. Ochterski, G. A. Petersson, *J. Chem. Phys.* **2000**, *112*, 6532–6542; b) S. Parthiban, G. de Oliveira, J. M. L. Martin, *J. Phys. Chem. A* **2001**, *105*, 895–904.
[16] a) B. Ruscic, R. E. Pinzon, M. L. Morton, G. von Laszevski, S. J. Bittner, S. G. Nijsure, K. A. Amin, M. Minkoff, A. F. Wagner, *J. Phys. Chem. A* **2004**, *108*, 9979–9997; b) B. Ruscic, D. H. Bross, *Active Thermochemical Tables (ATcT), Ver. 1.130*, **2023**.
[17] a) M. Sućeska, *Propellants, Explos., Pyrotech.* **1991**, *16*, 197–202; b) M. Sućeska, *EXPLO5 V7.01.01*, OZM Research, Hrochův Týnec (Czech Republic), **2023**.
[18] T. J. Koller, S. M. J. Endraß, M. Rösch, K. Witthaut, T. M. Klapötke, W. Schnick, *Angew. Chem. Int. Ed.* **2024**, *63*, e202404927.
[19] T. M. Klapötke, C. M. Sabaté, *Z. Anorg. Allg. Chem.* **2010**, *636*, 163–175.
[20] P. Li, H. D. Arman, H. Wang, L. Weng, K. Alfooty, R. F. Angawi, B. Chen, *Cryst. Growth Des.* **2015**, *15*, 1871–1875.
[21] S. Söllradl, M. Greiwe, V. J. Bukas, M. R. Buchner, M. Widenmeyer, T. Kandemir, T. Zweifel, A. Senyshyn, S. Günther, T. Nilges, A. Türler, R. Niewa, *Chem. Mater.* **2015**, *27*, 4188–4195.
[22] T. J. Prior, J. A. Armstrong, D. M. Benoit, K. L. Marshall, *CrystEngComm* **2013**, *15*, 5838–5843.

Author Contributions

Thaddäus J. Koller: Conceptualization (Lead), Formal Analysis (Lead), Investigation (Lead), Validation (Equal), Visualization (Lead), Writing – original draft (Lead), Writing – review & editing (Lead)
Kristian Witthaut: Methodology (Lead), Formal Analysis (Supporting), Validation (Supporting), Writing – review & editing (Supporting)
Reinhard M. Pritzl: Formal Analysis (Supporting), Validation (Supporting), Writing – review & editing (Supporting)
Simon M. J. Endraß: Methodology (Supporting), Formal Analysis (Supporting), Validation (Supporting), Visualization (Supporting), Writing – review & editing (Supporting)
Markus Rösch: Methodology (Supporting), Formal Analysis (Supporting), Validation (Supporting), Writing – review & editing (Supporting)
Georg Krach: Formal Analysis (Supporting), Validation (Supporting), Writing – review & editing (Supporting)
Nadine Lammer: Investigation (Supporting), Validation (Supporting), Writing – review & editing (Supporting)
Thomas M. Klapötke: Funding acquisition (Supporting), Resources (Supporting), Supervision (Supporting)
Wolfgang Schnick: Funding acquisition (Lead), Resources (Lead), Supervision (Lead), Validation (Equal), Writing – original draft (Supporting), Writing – review & editing (Supporting)
