# Supplementary material for: Resolving a Nearly Two‐Centuries‐Old Mystery: On the Structural Chemistry and Physicochemical Properties of Compounds Associated With the Term Ammelide
Source: Chemistry. 2025 Dec 31;32(9):e03587. doi: 10.1002/chem.202503587 (PMC12958097; doi:10.1002/chem.202503587)

checkCIF/PLATON report

Structure factors have been supplied for datablock(s) shelx

THIS REPORT IS FOR GUIDANCE ONLY. IF USED AS PART OF A REVIEW PROCEDURE FOR PUBLICATION, IT SHOULD NOT REPLACE THE EXPERTISE OF AN EXPERIENCED CRYSTALLOGRAPHIC REFEREE.

No syntax errors found. CIF dictionary Interpreting this report

Datablock: shelx

Bond precision: N- C = 0.0027 A Wavelength=0.71073

Cell:	a=14.726(4)	b=9.570(2)	c=3.5570(9)
	alpha=90	beta=92.571(9)	gamma=90
Temperature:	173 K		

	Calculated	Reported
Volume	500.8(2)	500.8(2)
Space group	C 2/m	C 2/m
Hall group	-C 2y	-C 2y
Moiety formula	C6 H9 N9 O3	?
Sum formula	C6 H9 N9 O3	C6 H9 N9 O3
Mr	255.22	255.22
Dx,g cm-3	1.692	1.693
Z	2	2
Mu (mm-1)	0.139	0.139
F000	264.0	264.0
F000'	264.10	
h,k,lmax	17,11,4	17,11,4
Nref	470	466
Tmin,Tmax	0.993,0.993	0.822,1.000
Tmin'	0.993	

Correction method= # Reported T Limits: Tmin=0.822 Tmax=1.000
AbsCorr = MULTI-SCAN

Data completeness= 0.991 Theta(max)= 24.980

R(reflections)= 0.0598(410) wR2(reflections)= 0.1607(466)

S = 1.111 Npar= 51

The following ALERTS were generated. Each ALERT has the format
test-name_ALERT_alert-type_alert-level.
Click on the hyperlinks for more details of the test.

Alert level C

```
PLAT088_ALERT_3_C Poor Data / Parameter Ratio ....................       9.14 Note
PLAT250_ALERT_2_C Large U3/U1 Ratio for <U(i,j)> Tensor(Resd    1)        2.6 Note
PLAT906_ALERT_3_C Large K Value in the Analysis of Variance ......      2.688 Check
PLAT911_ALERT_3_C Missing FCF Refl Between Thmin & STh/L=    0.594          2 Report
               -9  9  1,   2  0  1,
PLAT975_ALERT_2_C Check Calcd Resid. Dens.  0.88Ang From N4      .       0.50 eA-3
```

Alert level G

```
PLAT002_ALERT_2_G Number of Distance or Angle Restraints on AtSite          6 Note
PLAT007_ALERT_5_G Number of Unrefined Donor-H Atoms .............           5 Report
              H1    H2    H3    H4    H5
PLAT171_ALERT_4_G The CIF-Embedded .res File Contains EADP Records          2 Report
PLAT176_ALERT_4_G The CIF-Embedded .res File Contains SADI Records          2 Report
PLAT299_ALERT_4_G Atom Site Occupancy Constrained at ............         0.5 Check
              O1      O2      N1      N2      H1      H2      H3      H4
              H5
PLAT301_ALERT_3_G Main Residue  Disorder .............(Resd    1)         40% Note
PLAT304_ALERT_4_G Non-Integer Number of Atoms in ..... (Resd    1)      13.50 Check
PLAT779_ALERT_4_G Suspect or Irrelevant (Bond) Angle(s) in CIF ...       2.00 Deg.
              O1   -C1   -N1      1_555   1_555   1_555 ......    #    3 Check
PLAT779_ALERT_4_G Suspect or Irrelevant (Bond) Angle(s) in CIF ...       7.00 Deg.
              O2   -C2   -N2      1_555   1_555   1_555 ......    #    9 Check
PLAT811_ALERT_5_G No ADDSYM Analysis: Too Many Excluded Atoms ....          ! Info
PLAT860_ALERT_3_G Number of Least-Squares Restraints ............           2 Note
PLAT883_ALERT_1_G Absent Datum for _atom_sites_solution_primary ..     Please Do !
PLAT899_ALERT_4_G SHELXL2018 is Outdated   and Succeeded by SHELXL     2019/3 Note
PLAT909_ALERT_3_G Percentage of I>2sig(I) Data at Theta(Max) Still        84% Note
PLAT910_ALERT_3_G Missing FCF Reflection(s) Below Theta(Min)[Deg]=       4.26 Note
                1  1  0,   2  0  0,
PLAT913_ALERT_3_G Missing # of Very Strong Reflections in FCF ....          1 Note
                2  0  1,
PLAT961_ALERT_5_G Dataset Contains no Negative Intensities ......     Please Check
PLAT967_ALERT_5_G Note: Two-Theta Cutoff Value in Embedded .res ..       50.0 Degree
PLAT969_ALERT_5_G The 'Henn et al.' R-Factor-gap value ..........      5.120 Note
              Predicted wR2: Based on SigI**2  3.14 or SHELX Weight 14.47
```

0 **ALERT level A** = Most likely a serious problem - resolve or explain
0 **ALERT level B** = A potentially serious problem, consider carefully
5 **ALERT level C** = Check. Ensure it is not caused by an omission or oversight
19 **ALERT level G** = General information/check it is not something unexpected

1 ALERT type 1 CIF construction/syntax error, inconsistent or missing data
3 ALERT type 2 Indicator that the structure model may be wrong or deficient
8 ALERT type 3 Indicator that the structure quality may be low
7 ALERT type 4 Improvement, methodology, query or suggestion
5 ALERT type 5 Informative message, check

It is advisable to attempt to resolve as many as possible of the alerts in all categories. Often the minor alerts point to easily fixed oversights, errors and omissions in your CIF or refinement strategy, so attention to these fine details can be worthwhile. In order to resolve some of the more serious problems it may be necessary to carry out additional measurements or structure refinements. However, the purpose of your study may justify the reported deviations and the more serious of these should normally be commented upon in the discussion or experimental section of a paper or in the "special_details" fields of the CIF. checkCIF was carefully designed to identify outliers and unusual parameters, but every test has its limitations and alerts that are not important in a particular case may appear. Conversely, the absence of alerts does not guarantee there are no aspects of the results needing attention. It is up to the individual to critically assess their own results and, if necessary, seek expert advice.

Publication of your CIF in IUCr journals

A basic structural check has been run on your CIF. These basic checks will be run on all CIFs submitted for publication in IUCr journals (*Acta Crystallographica*, *Journal of Applied Crystallography*, *Journal of Synchrotron Radiation*); however, if you intend to submit to *Acta Crystallographica Section C* or *E* or *IUCrData*, you should make sure that full publication checks are run on the final version of your CIF prior to submission.

Publication of your CIF in other journals

Please refer to the *Notes for Authors* of the relevant journal for any special instructions relating to CIF submission.

PLATON version of 04/06/2025; check.def file version of 30/05/2025

Datablock shelx - ellipsoid plot

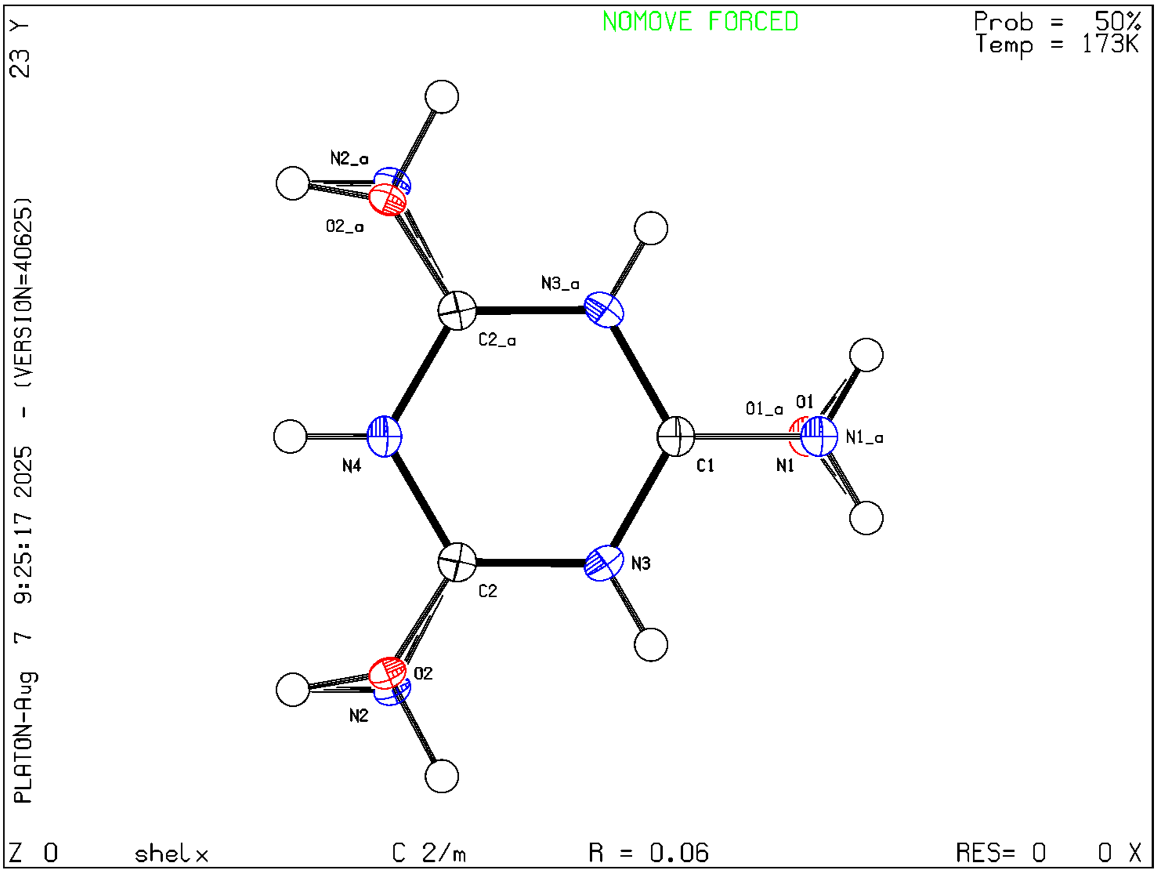

checkCIF/PLATON report

Structure factors have been supplied for datablock(s) shelx

THIS REPORT IS FOR GUIDANCE ONLY. IF USED AS PART OF A REVIEW PROCEDURE FOR PUBLICATION, IT SHOULD NOT REPLACE THE EXPERTISE OF AN EXPERIENCED CRYSTALLOGRAPHIC REFEREE.

No syntax errors found.   CIF dictionary   Interpreting this report

Datablock: shelx

```
Bond precision:   N- C = 0.0023 A          Wavelength=0.71073

Cell:              a=16.5876(14)     b=9.5937(8)          c=6.5959(6)
                   alpha=90          beta=104.985(3)      gamma=90
Temperature:       173 K

                 Calculated                    Reported
Volume           1013.95(15)                   1013.95(15)
Space group      C 2/c                         C 2/c
Hall group       -C 2yc                        -C 2yc
Moiety formula   2(C3 H4.50 N4.50 O1.50)       ?
Sum formula      C6 H9 N9 O3                   C6 H9 N9 O3
Mr               255.22                        255.22
Dx,g cm-3        1.672                         1.672
Z                4                             4
Mu (mm-1)        0.137                         0.137
F000             528.0                         528.0
F000'            528.20
h,k,lmax         19,11,7                       19,11,7
Nref             897                           894
Tmin,Tmax        0.993,0.993                   0.906,1.000
Tmin'            0.993

Correction method= # Reported T Limits: Tmin=0.906 Tmax=1.000
AbsCorr = MULTI-SCAN

Data completeness= 0.997           Theta(max)= 24.992

                                                     wR2(reflections)=
R(reflections)= 0.0439( 664)                         0.1143( 894)

S = 1.037                  Npar= 91
```

The following ALERTS were generated. Each ALERT has the format
test-name_ALERT_alert-type_alert-level.
Click on the hyperlinks for more details of the test.

Alert level C

```
PLAT088_ALERT_3_C Poor Data / Parameter Ratio ....................       9.82 Note
PLAT906_ALERT_3_C Large K Value in the Analysis of Variance ......      8.852 Check
```

Alert level G

```
PLAT002_ALERT_2_G Number of Distance or Angle Restraints on AtSite          9 Note
PLAT007_ALERT_5_G Number of Unrefined Donor-H Atoms ..............          9 Report
              H1    H2    H3    H4    H5    H6    H7    H8    H9
PLAT171_ALERT_4_G The CIF-Embedded .res File Contains EADP Records          3 Report
PLAT176_ALERT_4_G The CIF-Embedded .res File Contains SADI Records          2 Report
PLAT299_ALERT_4_G Atom Site Occupancy Constrained at .............        0.5 Check
              O1      O2      O3      N1      N2      N3      H1      H2
              H3      H4      H5      H6      H7      H8      H9
PLAT301_ALERT_3_G Main Residue  Disorder ..............(Resd    1)        33% Note
PLAT304_ALERT_4_G Non-Integer Number of Atoms in ..... (Resd    1)      13.50 Check
PLAT779_ALERT_4_G Suspect or Irrelevant (Bond) Angle(s) in CIF ...       4.00 Deg.
              O1   -C1   -N1      1_555   1_555   1_555 ......     #    4 Check
PLAT779_ALERT_4_G Suspect or Irrelevant (Bond) Angle(s) in CIF ...       5.00 Deg.
              O2   -C2   -N2      1_555   1_555   1_555 ......     #   10 Check
PLAT779_ALERT_4_G Suspect or Irrelevant (Bond) Angle(s) in CIF ...       4.00 Deg.
              O3   -C3   -N3      1_555   1_555   1_555 ......     #   16 Check
PLAT811_ALERT_5_G No ADDSYM Analysis: Too Many Excluded Atoms ....          ! Info
PLAT860_ALERT_3_G Number of Least-Squares Restraints .............          6 Note
PLAT883_ALERT_1_G Absent Datum for _atom_sites_solution_primary ..     Please Do !
PLAT899_ALERT_4_G SHELXL2018 is Outdated   and Succeeded by SHELXL     2019/3 Note
PLAT909_ALERT_3_G Percentage of I>2sig(I) Data at Theta(Max) Still        58% Note
PLAT910_ALERT_3_G Missing FCF Reflection(s) Below Theta(Min)[Deg]=       3.78 Note
                1  1  0,   2  0  0,
PLAT913_ALERT_3_G Missing # of Very Strong Reflections in FCF ....          1 Note
                0  0  2,
PLAT933_ALERT_2_G Number of HKL-OMIT Records in Embedded .res File          1 Note
                0  0  2,
PLAT961_ALERT_5_G Dataset Contains no Negative Intensities .......     Please Check
PLAT967_ALERT_5_G Note: Two-Theta Cutoff Value in Embedded .res ..       50.0 Degree
PLAT969_ALERT_5_G The 'Henn et al.' R-Factor-gap value ...........      3.585 Note
              Predicted wR2: Based on SigI**2  3.19 or SHELX Weight 11.03
```

   0 **ALERT level A** = Most likely a serious problem - resolve or explain
   0 **ALERT level B** = A potentially serious problem, consider carefully
   2 **ALERT level C** = Check. Ensure it is not caused by an omission or oversight
  21 **ALERT level G** = General information/check it is not something unexpected

```
   1 ALERT type 1 CIF construction/syntax error, inconsistent or missing data
   2 ALERT type 2 Indicator that the structure model may be wrong or deficient
   7 ALERT type 3 Indicator that the structure quality may be low
   8 ALERT type 4 Improvement, methodology, query or suggestion
   5 ALERT type 5 Informative message, check
```

It is advisable to attempt to resolve as many as possible of the alerts in all categories. Often the minor alerts point to easily fixed oversights, errors and omissions in your CIF or refinement strategy, so attention to these fine details can be worthwhile. In order to resolve some of the more serious problems it may be necessary to carry out additional measurements or structure refinements. However, the purpose of your study may justify the reported deviations and the more serious of these should normally be commented upon in the discussion or experimental section of a paper or in the "special_details" fields of the CIF. checkCIF was carefully designed to identify outliers and unusual parameters, but every test has its limitations and alerts that are not important in a particular case may appear. Conversely, the absence of alerts does not guarantee there are no aspects of the results needing attention. It is up to the individual to critically assess their own results and, if necessary, seek expert advice.

Publication of your CIF in IUCr journals

A basic structural check has been run on your CIF. These basic checks will be run on all CIFs submitted for publication in IUCr journals (*Acta Crystallographica*, *Journal of Applied Crystallography*, *Journal of Synchrotron Radiation*); however, if you intend to submit to *Acta Crystallographica Section C* or *E* or *IUCrData*, you should make sure that full publication checks are run on the final version of your CIF prior to submission.

Publication of your CIF in other journals

Please refer to the *Notes for Authors* of the relevant journal for any special instructions relating to CIF submission.

PLATON version of 04/06/2025; check.def file version of 30/05/2025

Datablock shelx - ellipsoid plot

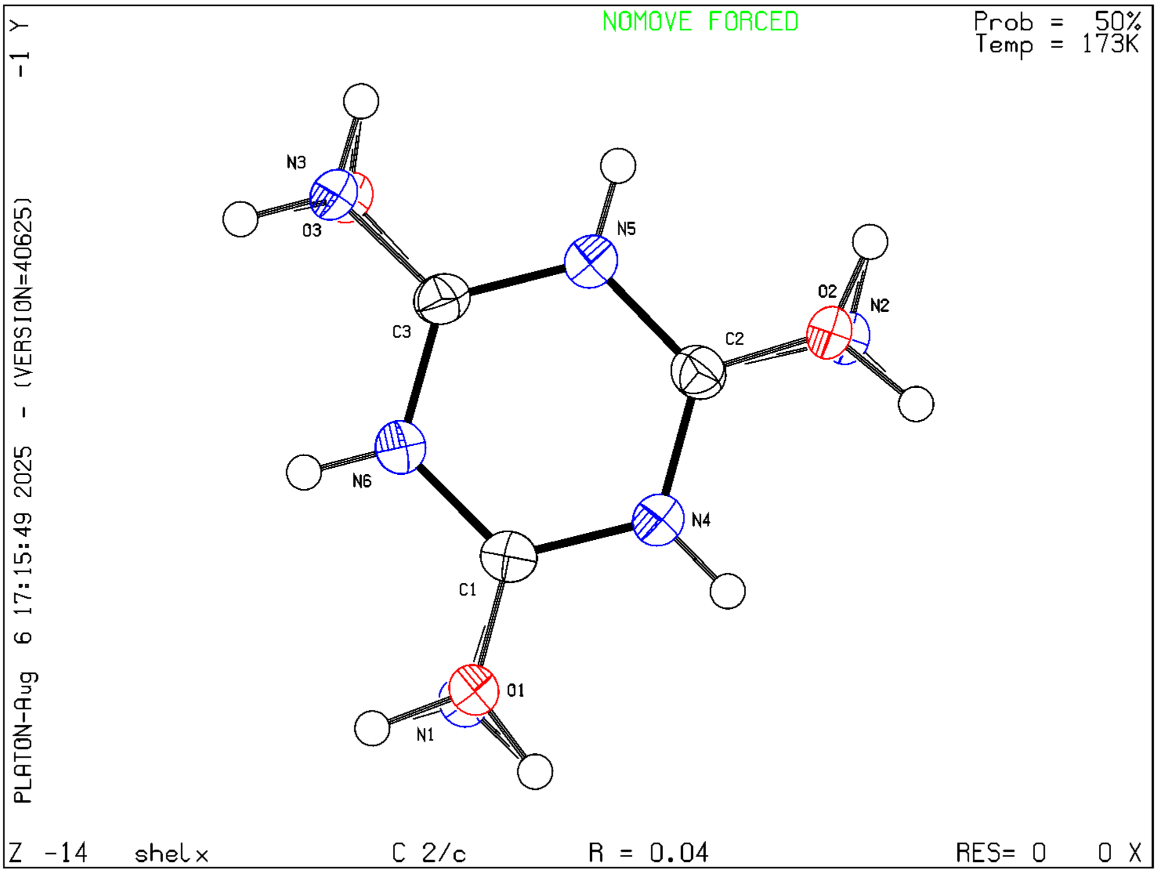

checkCIF/PLATON report

Structure factors have been supplied for datablock(s) shelx

THIS REPORT IS FOR GUIDANCE ONLY. IF USED AS PART OF A REVIEW PROCEDURE FOR PUBLICATION, IT SHOULD NOT REPLACE THE EXPERTISE OF AN EXPERIENCED CRYSTALLOGRAPHIC REFEREE.

No syntax errors found. CIF dictionary Interpreting this report

Datablock: shelx

```
Bond precision:   N- C = 0.0090 A          Wavelength=0.71073

Cell:                a=9.5855(6)       b=9.5855(6)       c=9.4906(4)
                     alpha=90          beta=90           gamma=120
Temperature:         173 K
```

	Calculated	Reported
Volume	755.19(10)	755.19(10)
Space group	P 31 2 1	P 31 2 1
Hall group	P 31 2"	P 31 2"
Moiety formula	2(C3 H4.50 N4.50 O1.50)	?
Sum formula	C6 H9 N9 O3	C6 H9 N9 O3
Mr	255.22	255.22
Dx,g cm-3	1.684	1.684
Z	3	3
Mu (mm-1)	0.138	0.138
F000	396.0	396.0
F000'	396.15	
h,k,lmax	11,11,11	11,11,11
Nref	927[548]	923
Tmin,Tmax	0.993,0.993	0.900,1.000
Tmin'	0.993	

```
Correction method= # Reported T Limits: Tmin=0.900 Tmax=1.000
AbsCorr = MULTI-SCAN

Data completeness= 1.68/1.00        Theta(max)= 25.346

R(reflections)= 0.0499( 753)                    wR2(reflections)=
                                                0.1358( 923)
S = 1.157                   Npar= 85
```

The following ALERTS were generated. Each ALERT has the format
test-name_ALERT_alert-type_alert-level.
Click on the hyperlinks for more details of the test.

Alert level C

```
DIFMX02_ALERT_1_C  The maximum difference density is > 0.1*ZMAX*0.75
            The relevant atom site should be identified.
STRVA01_ALERT_4_C           Flack test results are meaningless.
           From the CIF: _refine_ls_abs_structure_Flack    0.700
           From the CIF: _refine_ls_abs_structure_Flack_su    1.000
PLAT089_ALERT_3_C Poor Data / Parameter Ratio (Zmax < 18) ........       6.40 Note
PLAT094_ALERT_2_C Ratio of Maximum / Minimum Residual Density ....       2.13 Report
PLAT097_ALERT_2_C Large Reported Max.  (Positive) Residual Density       0.70 eA-3
PLAT215_ALERT_3_C Disordered O1        has ADP max/min Ratio .....        3.6 Note
PLAT215_ALERT_3_C Disordered O2        has ADP max/min Ratio .....        3.2 Note
PLAT215_ALERT_3_C Disordered O3        has ADP max/min Ratio .....        3.2 Note
PLAT215_ALERT_3_C Disordered N1        has ADP max/min Ratio .....        3.6 Note
PLAT215_ALERT_3_C Disordered N2        has ADP max/min Ratio .....        3.2 Note
PLAT215_ALERT_3_C Disordered N3        has ADP max/min Ratio .....        3.2 Note
PLAT230_ALERT_2_C Hirshfeld Test Diff for    N4       --C2       .        5.3 s.u.
PLAT250_ALERT_2_C Large U3/U1 Ratio for <U(i,j)> Tensor(Resd    1)        2.7 Note
PLAT907_ALERT_2_C Flack x > 0.5, Structure Needs to be Inverted? .       0.70 Check
PLAT911_ALERT_3_C Missing FCF Refl Between Thmin & STh/L=    0.600          3 Report
                0  3  1,   0  0  6,   0  0  9,
PLAT927_ALERT_1_C Reported and Calculated  wR2 Differ by .........    -0.0018 Check
PLAT975_ALERT_2_C Check Calcd Resid. Dens.  0.91Ang From N6      .       0.44 eA-3
```

Alert level G

```
PLAT002_ALERT_2_G Number of Distance or Angle Restraints on AtSite          9 Note
PLAT007_ALERT_5_G Number of Unrefined Donor-H Atoms ..............          9 Report
              H1    H2    H3    H4    H5    H6    H7    H8    H9
PLAT032_ALERT_4_G Std. Uncertainty on Flack Parameter Value High .      1.000 Report
PLAT171_ALERT_4_G The CIF-Embedded .res File Contains EADP Records          2 Report
PLAT176_ALERT_4_G The CIF-Embedded .res File Contains SADI Records          2 Report
PLAT299_ALERT_4_G Atom Site Occupancy Constrained at ............         0.5 Check
              O1      O2      O3      N1      N2      N3      H1      H2
              H3      H4      H5      H6      H7      H8      H9
PLAT301_ALERT_3_G Main Residue  Disorder ..............(Resd    1)        33% Note
PLAT304_ALERT_4_G Non-Integer Number of Atoms in ..... (Resd    1)      13.50 Check
PLAT779_ALERT_4_G Suspect or Irrelevant (Bond) Angle(s) in CIF ...       1.00 Deg.
              O1   -C1   -N1      1_555   1_555   1_555 ......     #    5 Check
PLAT779_ALERT_4_G Suspect or Irrelevant (Bond) Angle(s) in CIF ...       8.00 Deg.
              O2   -C2   -N2      1_555   1_555   1_555 ......     #   11 Check
PLAT779_ALERT_4_G Suspect or Irrelevant (Bond) Angle(s) in CIF ...       7.00 Deg.
              O3   -C3   -N3      1_555   1_555   1_555 ......     #   17 Check
PLAT811_ALERT_5_G No ADDSYM Analysis: Too Many Excluded Atoms ....          ! Info
PLAT860_ALERT_3_G Number of Least-Squares Restraints ............           6 Note
PLAT883_ALERT_1_G Absent Datum for _atom_sites_solution_primary ..     Please Do !
PLAT899_ALERT_4_G SHELXL2018 is Outdated   and Succeeded by SHELXL     2019/3 Note
PLAT910_ALERT_3_G Missing FCF Reflection(s) Below Theta(Min)[Deg]=       3.26 Note
                0  1  0,
PLAT916_ALERT_2_G Hooft y and Flack x Parameter Values Differ by .       0.80 Check
PLAT933_ALERT_2_G Number of HKL-OMIT Records in Embedded .res File          3 Note
                0  0  6,   0  0  9,   0  3  1,
```

```
PLAT969_ALERT_5_G The 'Henn et al.' R-Factor-gap value ..........      6.963 Note
            Predicted wR2: Based on SigI**2  1.95 or SHELX Weight 11.74
```

```
  0 ALERT level A = Most likely a serious problem - resolve or explain
  0 ALERT level B = A potentially serious problem, consider carefully
 17 ALERT level C = Check. Ensure it is not caused by an omission or oversight
 19 ALERT level G = General information/check it is not something unexpected

  3 ALERT type 1 CIF construction/syntax error, inconsistent or missing data
  9 ALERT type 2 Indicator that the structure model may be wrong or deficient
 11 ALERT type 3 Indicator that the structure quality may be low
 10 ALERT type 4 Improvement, methodology, query or suggestion
  3 ALERT type 5 Informative message, check
```

It is advisable to attempt to resolve as many as possible of the alerts in all categories. Often the minor alerts point to easily fixed oversights, errors and omissions in your CIF or refinement strategy, so attention to these fine details can be worthwhile. In order to resolve some of the more serious problems it may be necessary to carry out additional measurements or structure refinements. However, the purpose of your study may justify the reported deviations and the more serious of these should normally be commented upon in the discussion or experimental section of a paper or in the "special_details" fields of the CIF. checkCIF was carefully designed to identify outliers and unusual parameters, but every test has its limitations and alerts that are not important in a particular case may appear. Conversely, the absence of alerts does not guarantee there are no aspects of the results needing attention. It is up to the individual to critically assess their own results and, if necessary, seek expert advice.

Publication of your CIF in IUCr journals

A basic structural check has been run on your CIF. These basic checks will be run on all CIFs submitted for publication in IUCr journals (*Acta Crystallographica*, *Journal of Applied Crystallography*, *Journal of Synchrotron Radiation*); however, if you intend to submit to *Acta Crystallographica Section C* or *E* or *IUCrData*, you should make sure that full publication checks are run on the final version of your CIF prior to submission.

Publication of your CIF in other journals

Please refer to the *Notes for Authors* of the relevant journal for any special instructions relating to CIF submission.

PLATON version of 04/06/2025; check.def file version of 30/05/2025

Datablock shelx - ellipsoid plot

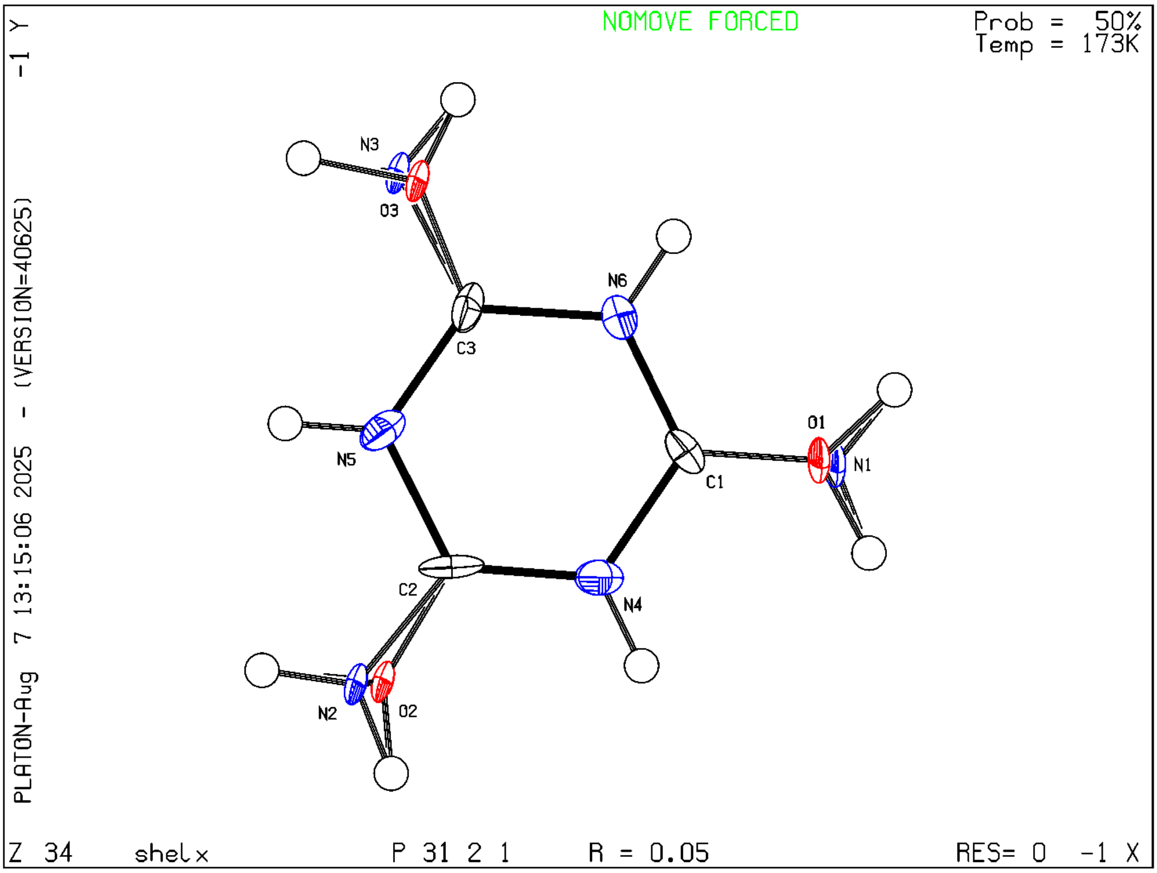

checkCIF/PLATON report

Structure factors have been supplied for datablock(s) shelx

THIS REPORT IS FOR GUIDANCE ONLY. IF USED AS PART OF A REVIEW PROCEDURE FOR PUBLICATION, IT SHOULD NOT REPLACE THE EXPERTISE OF AN EXPERIENCED CRYSTALLOGRAPHIC REFEREE.

No syntax errors found. CIF dictionary Interpreting this report

Datablock: shelx

Bond precision:	O- C = 0.0060 A		Wavelength=0.71073
Cell:	a=6.3345(12)	b=9.7509(13)	c=7.9355(14)
	alpha=90	beta=98.535(9)	gamma=90
Temperature:	173 K		

	Calculated	Reported
Volume	484.72(14)	484.72(14)
Space group	P 21/c	P 21/c
Hall group	-P 2ybc	-P 2ybc
Moiety formula	C3 H4 N4 O2	?
Sum formula	C3 H4 N4 O2	C3 H4 N4 O2
Mr	128.10	128.10
Dx,g cm-3	1.755	1.755
Z	4	4
Mu (mm-1)	0.149	0.149
F000	264.0	264.0
F000’	264.12	
h,k,lmax	7,11,9	7,11,9
Nref	854	801
Tmin,Tmax	0.993,0.993	0.891,1.000
Tmin’	0.993	

Correction method= # Reported T Limits: Tmin=0.891 Tmax=1.000
AbsCorr = MULTI-SCAN

Data completeness= 0.938 Theta(max)= 24.993

R(reflections)= 0.0947(509) wR2(reflections)= 0.2448(801)

S = 1.019 Npar= 82

The following ALERTS were generated. Each ALERT has the format
test-name_ALERT_alert-type_alert-level.
Click on the hyperlinks for more details of the test.

Alert level A

PLAT029_ALERT_3_A _diffrn_measured_fraction_theta_full value Low . 0.938 Why?

Author Response: This results from the intergrowth of the measured crystal. As a consequence, several reflections were removed during the absorption correction. However, the resulting data was still sufficient for a reasonable structure solution.

Alert level B

PLAT097_ALERT_2_B Large Reported Max. (Positive) Residual Density 0.97 eA-3

Author Response: This results from the intergrowth of the measured crystal. As a consequence, several reflections have higher intensity than expected, which in return leads to this high positve residual density. However, the resulting data was still sufficient for a reasonable structure solution.

```
PLAT911_ALERT_3_B Missing FCF Refl Between Thmin & STh/L=    0.594          53 Report
                3 10  0,   2 11  0,  -6  7  1,  -5  8  1,  -3 10  1,  -2 10  1,
               -2 11  1,   2 11  1,  -4  9  2,  -3 10  2,  -2 10  2,   0 10  2,
                6  2  3,   6  3  3,   6  4  3,   5  6  3,   5  7  3,  -4  9  3,
               -3 10  3,  -2 10  3,  -1 10  3,   0 10  3,   6  0  4,   6  1  4,
                6  2  4,   5  6  4,  -1  9  4,   3  9  4,  -2 10  4,   5  4  5,
                   (   23 More Missing: see the .ckf listing file)
```

Author Response: This results from the intergrowth of the measured crystal. As a consequence, several reflections were removed during the absorption correction. However, the resulting data was still sufficient for a reasonable structure solution.

Alert level C

```
DIFMX02_ALERT_1_C  The maximum difference density is > 0.1*ZMAX*0.75
            The relevant atom site should be identified.
PLAT088_ALERT_3_C Poor Data / Parameter Ratio ....................       9.77 Note
PLAT094_ALERT_2_C Ratio of Maximum / Minimum Residual Density ....       3.37 Report
PLAT250_ALERT_2_C Large U3/U1 Ratio for <U(i,j)> Tensor(Resd    1)        2.1 Note
PLAT906_ALERT_3_C Large K Value in the Analysis of Variance ......     42.717 Check
PLAT906_ALERT_3_C Large K Value in the Analysis of Variance ......      3.395 Check
PLAT906_ALERT_3_C Large K Value in the Analysis of Variance ......      2.027 Check
PLAT975_ALERT_2_C Check Calcd Resid. Dens.  0.98Ang From O1       .      0.69 eA-3
PLAT975_ALERT_2_C Check Calcd Resid. Dens.  1.07Ang From N4       .      0.64 eA-3
```

Alert level G

```
PLAT007_ALERT_5_G Number of Unrefined Donor-H Atoms .............          4 Report
              H1    H2    H3    H4
PLAT883_ALERT_1_G Absent Datum for _atom_sites_solution_primary ..     Please Do !
PLAT899_ALERT_4_G SHELXL2018 is Outdated   and Succeeded by SHELXL     2019/3 Note
PLAT909_ALERT_3_G Percentage of I>2sig(I) Data at Theta(Max) Still        58% Note
PLAT933_ALERT_2_G Number of HKL-OMIT Records in Embedded .res File          9 Note
               -3 10  2,  -4  9  3,  -2 10  2,  -4  9  2,  -3 10  1,   5  7  3,
                5  6  4,  -2 10  1,   6  3  3,
PLAT961_ALERT_5_G Dataset Contains no Negative Intensities .......     Please Check
PLAT967_ALERT_5_G Note: Two-Theta Cutoff Value in Embedded .res ..       50.0 Degree
PLAT969_ALERT_5_G The 'Henn et al.' R-Factor-gap value ..........      6.517 Note
              Predicted wR2: Based on SigI**2  3.76 or SHELX Weight 24.03
```

---

1 **ALERT level A** = Most likely a serious problem - resolve or explain
2 **ALERT level B** = A potentially serious problem, consider carefully
9 **ALERT level C** = Check. Ensure it is not caused by an omission or oversight
8 **ALERT level G** = General information/check it is not something unexpected

2 ALERT type 1 CIF construction/syntax error, inconsistent or missing data
6 ALERT type 2 Indicator that the structure model may be wrong or deficient
7 ALERT type 3 Indicator that the structure quality may be low
1 ALERT type 4 Improvement, methodology, query or suggestion
4 ALERT type 5 Informative message, check

It is advisable to attempt to resolve as many as possible of the alerts in all categories. Often the minor alerts point to easily fixed oversights, errors and omissions in your CIF or refinement strategy, so attention to these fine details can be worthwhile. In order to resolve some of the more serious problems it may be necessary to carry out additional measurements or structure refinements. However, the purpose of your study may justify the reported deviations and the more serious of these should normally be commented upon in the discussion or experimental section of a paper or in the "special_details" fields of the CIF. checkCIF was carefully designed to identify outliers and unusual parameters, but every test has its limitations and alerts that are not important in a particular case may appear. Conversely, the absence of alerts does not guarantee there are no aspects of the results needing attention. It is up to the individual to critically assess their own results and, if necessary, seek expert advice.

Publication of your CIF in IUCr journals

A basic structural check has been run on your CIF. These basic checks will be run on all CIFs submitted for publication in IUCr journals (*Acta Crystallographica*, *Journal of Applied Crystallography*, *Journal of Synchrotron Radiation*); however, if you intend to submit to *Acta Crystallographica Section C* or *E* or *IUCrData*, you should make sure that full publication checks are run on the final version of your CIF prior to submission.

Publication of your CIF in other journals

Please refer to the *Notes for Authors* of the relevant journal for any special instructions relating to CIF submission.

PLATON version of 04/06/2025; check.def file version of 30/05/2025

Datablock shelx - ellipsoid plot

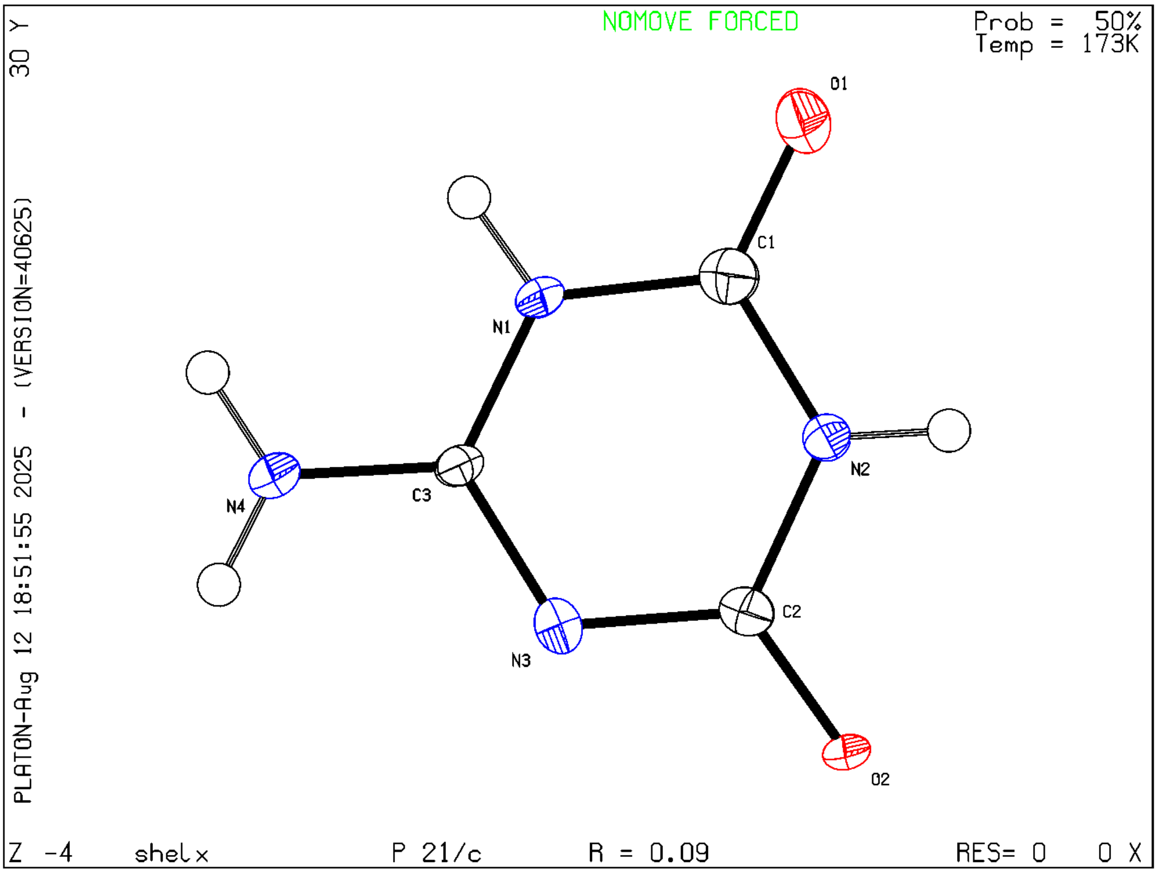

checkCIF/PLATON report

Structure factors have been supplied for datablock(s) shelx

THIS REPORT IS FOR GUIDANCE ONLY. IF USED AS PART OF A REVIEW PROCEDURE FOR PUBLICATION, IT SHOULD NOT REPLACE THE EXPERTISE OF AN EXPERIENCED CRYSTALLOGRAPHIC REFEREE.

No syntax errors found.   CIF dictionary   Interpreting this report

Datablock: shelx

Bond precision: N- C = 0.0023 A   Wavelength=0.71073

Cell:	a=9.2996(4)	b=6.2638(3)	c=13.4898(6)
	alpha=90	beta=108.019(2)	gamma=90
Temperature:	173 K		

	Calculated	Reported
Volume	747.25(6)	747.25(6)
Space group	P 21/c	P 21/c
Hall group	-P 2ybc	-P 2ybc
Moiety formula	C3 H6 N5 O, N O3	?
Sum formula	C3 H6 N6 O4	C3 H6 N6 O4
Mr	190.14	190.14
Dx,g cm-3	1.690	1.690
Z	4	4
Mu (mm-1)	0.153	0.153
F000	392.0	392.0
F000'	392.21	
h,k,lmax	12,8,17	12,8,17
Nref	1714	1710
Tmin,Tmax	0.992,0.992	0.931,1.000
Tmin'	0.992	

Correction method= # Reported T Limits: Tmin=0.931 Tmax=1.000
AbsCorr = MULTI-SCAN

Data completeness= 0.998   Theta(max)= 27.497

R(reflections)= 0.0458(1373)   wR2(reflections)= 0.1060(1710)

S = 1.082   Npar= 142

The following ALERTS were generated. Each ALERT has the format
test-name_ALERT_alert-type_alert-level.
Click on the hyperlinks for more details of the test.

Alert level C

```
PLAT906_ALERT_3_C Large K Value in the Analysis of Variance ......      8.775 Check
```

Alert level G

```
PLAT883_ALERT_1_G Absent Datum for _atom_sites_solution_primary ..     Please Do !
PLAT899_ALERT_4_G SHELXL2018 is Outdated   and Succeeded by SHELXL     2019/3 Note
PLAT910_ALERT_3_G Missing FCF Reflection(s) Below Theta(Min)[Deg]=       3.62 Note
                1  0  0,  -1  0  2,   0  0  2,
PLAT933_ALERT_2_G Number of HKL-OMIT Records in Embedded .res File          2 Note
               -1  0  2,   0  0  2,
PLAT961_ALERT_5_G Dataset Contains no Negative Intensities .......     Please Check
PLAT967_ALERT_5_G Note: Two-Theta Cutoff Value in Embedded .res ..       55.0 Degree
PLAT969_ALERT_5_G The 'Henn et al.' R-Factor-gap value ..........      4.099 Note
              Predicted wR2: Based on SigI**2  2.59 or SHELX Weight  9.80
```

0 **ALERT level A** = Most likely a serious problem - resolve or explain
0 **ALERT level B** = A potentially serious problem, consider carefully
1 **ALERT level C** = Check. Ensure it is not caused by an omission or oversight
7 **ALERT level G** = General information/check it is not something unexpected

1 ALERT type 1 CIF construction/syntax error, inconsistent or missing data
1 ALERT type 2 Indicator that the structure model may be wrong or deficient
2 ALERT type 3 Indicator that the structure quality may be low
1 ALERT type 4 Improvement, methodology, query or suggestion
3 ALERT type 5 Informative message, check

It is advisable to attempt to resolve as many as possible of the alerts in all categories. Often the minor alerts point to easily fixed oversights, errors and omissions in your CIF or refinement strategy, so attention to these fine details can be worthwhile. In order to resolve some of the more serious problems it may be necessary to carry out additional measurements or structure refinements. However, the purpose of your study may justify the reported deviations and the more serious of these should normally be commented upon in the discussion or experimental section of a paper or in the "special_details" fields of the CIF. checkCIF was carefully designed to identify outliers and unusual parameters, but every test has its limitations and alerts that are not important in a particular case may appear. Conversely, the absence of alerts does not guarantee there are no aspects of the results needing attention. It is up to the individual to critically assess their own results and, if necessary, seek expert advice.

Publication of your CIF in IUCr journals

A basic structural check has been run on your CIF. These basic checks will be run on all CIFs submitted for publication in IUCr journals (*Acta Crystallographica*, *Journal of Applied Crystallography*, *Journal of Synchrotron Radiation*); however, if you intend to submit to *Acta Crystallographica Section C* or *E* or *IUCrData*, you should make sure that full publication checks are run on the final version of your CIF prior to submission.

Publication of your CIF in other journals

Please refer to the *Notes for Authors* of the relevant journal for any special instructions relating to CIF submission.

PLATON version of 04/06/2025; check.def file version of 30/05/2025

Datablock shelx - ellipsoid plot

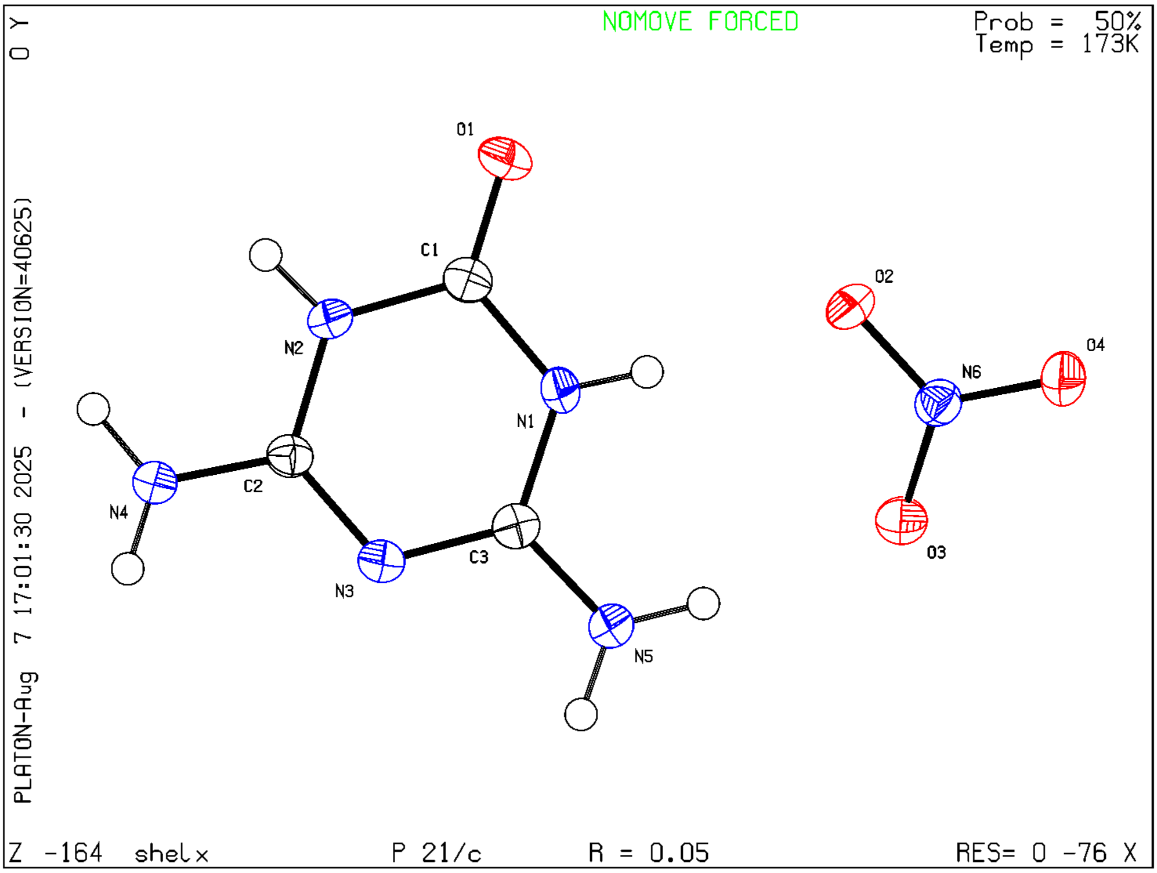

checkCIF/PLATON report

Structure factors have been supplied for datablock(s) shelx

THIS REPORT IS FOR GUIDANCE ONLY. IF USED AS PART OF A REVIEW PROCEDURE FOR PUBLICATION, IT SHOULD NOT REPLACE THE EXPERTISE OF AN EXPERIENCED CRYSTALLOGRAPHIC REFEREE.

No syntax errors found. CIF dictionary Interpreting this report

Datablock: shelx

Bond precision:	N- C = 0.0023 A		Wavelength=0.71073
Cell:	a=6.2224(4)	b=4.6285(2)	c=23.7842(15)
	alpha=90	beta=94.006(2)	gamma=90
Temperature:	173 K		

	Calculated	Reported
Volume	683.32(7)	683.32(7)
Space group	P 21/n	P 21/n
Hall group	-P 2yn	-P 2yn
Moiety formula	C3 H5 N4 O2, N O3	?
Sum formula	C3 H5 N5 O5	C3 H5 N5 O5
Mr	191.12	191.12
Dx,g cm-3	1.858	1.858
Z	4	4
Mu (mm-1)	0.175	0.175
F000	392.0	392.0
F000’	392.24	
h,k,lmax	7,5,29	7,5,29
Nref	1399	1465
Tmin,Tmax	0.991,0.991	0.834,1.000
Tmin’	0.991	

Correction method= # Reported T Limits: Tmin=0.834 Tmax=1.000
AbsCorr = MULTI-SCAN

Data completeness= 1.047 Theta(max)= 26.388

R(reflections)= 0.0319(1381) wR2(reflections)= 0.0844(1465)

S = 1.161 Npar= 139

```
The following ALERTS were generated. Each ALERT has the format
       test-name_ALERT_alert-type_alert-level.
Click on the hyperlinks for more details of the test.
```

Alert level B

```
PLAT430_ALERT_2_B Short Inter D...A Contact  O3       ..O3       .       2.66 Ang.
                                                1-x,2-y,1-z  =      3_676 Check
```

Author Response: The lone pairs of the oxygen atoms do not point directly towards each other and form instead hydrogen bonds to nearby hydrogen atoms.

```
PLAT430_ALERT_2_B Short Inter D...A Contact  O5       ..O5       .       2.68 Ang.
                                                2-x,1-y,1-z  =      3_766 Check
```

Author Response: The lone pairs of the oxygen atoms do not point directly towards each other and form instead hydrogen bonds to nearby hydrogen atoms.

Alert level C

```
PLAT088_ALERT_3_C Poor Data / Parameter Ratio ...................       9.90 Note
PLAT911_ALERT_3_C Missing FCF Refl Between Thmin & STh/L=    0.600         13 Report
                1  5  0,  -1  5  1,   1  5  1,   1  5  2,   0  0  4,   1  1  4,
               -1  5  6,  -1  5  7,  -1  5  8,  -1  5  9,  -1  5 12,   1  3 19,
               -3  0 21,
```

Alert level G

```
PLAT870_ALERT_4_G ALERTS Related to Twinning Effects Suppressed ..          ! Info
PLAT883_ALERT_1_G Absent Datum for _atom_sites_solution_primary ..     Please Do !
PLAT899_ALERT_4_G SHELXL2018 is Outdated   and Succeeded by SHELXL     2019/3 Note
PLAT910_ALERT_3_G Missing # of FCF Reflection(s) Below Theta(Min).          1 Note
                0  0  2,
PLAT912_ALERT_4_G Missing # of FCF Reflections Above STh/L=  0.600          9 Note
PLAT913_ALERT_3_G Missing # of Very Strong Reflections in FCF ....          1 Note
                1  1  4,
PLAT931_ALERT_5_G CIFcalcFCF  Twin Law         [ 1 0 4] Est.d BASF       0.18 Check
PLAT941_ALERT_3_G Average HKL Measurement Multiplicity ..........         1.0 Low
PLAT960_ALERT_3_G Number of Intensities with I < - 2*Sigma(I) ....          4 Check
PLAT967_ALERT_5_G Note: Two-Theta Cutoff Value in Embedded .res ..       55.0 Degree
PLAT969_ALERT_5_G The 'Henn et al.' R-Factor-gap value ..........       3.321 Note
              Predicted wR2: Based on SigI**2  2.54 or SHELX Weight  7.28
```

```
   0 ALERT level A = Most likely a serious problem - resolve or explain
   2 ALERT level B = A potentially serious problem, consider carefully
   2 ALERT level C = Check. Ensure it is not caused by an omission or oversight
  11 ALERT level G = General information/check it is not something unexpected
```

```
 1 ALERT type 1 CIF construction/syntax error, inconsistent or missing data
 2 ALERT type 2 Indicator that the structure model may be wrong or deficient
 6 ALERT type 3 Indicator that the structure quality may be low
 3 ALERT type 4 Improvement, methodology, query or suggestion
 3 ALERT type 5 Informative message, check
```

It is advisable to attempt to resolve as many as possible of the alerts in all categories. Often the minor alerts point to easily fixed oversights, errors and omissions in your CIF or refinement strategy, so attention to these fine details can be worthwhile. In order to resolve some of the more serious problems it may be necessary to carry out additional measurements or structure refinements. However, the purpose of your study may justify the reported deviations and the more serious of these should normally be commented upon in the discussion or experimental section of a paper or in the "special_details" fields of the CIF. checkCIF was carefully designed to identify outliers and unusual parameters, but every test has its limitations and alerts that are not important in a particular case may appear. Conversely, the absence of alerts does not guarantee there are no aspects of the results needing attention. It is up to the individual to critically assess their own results and, if necessary, seek expert advice.

Publication of your CIF in IUCr journals

A basic structural check has been run on your CIF. These basic checks will be run on all CIFs submitted for publication in IUCr journals (*Acta Crystallographica*, *Journal of Applied Crystallography*, *Journal of Synchrotron Radiation*); however, if you intend to submit to *Acta Crystallographica Section C* or *E* or *IUCrData*, you should make sure that full publication checks are run on the final version of your CIF prior to submission.

Publication of your CIF in other journals

Please refer to the *Notes for Authors* of the relevant journal for any special instructions relating to CIF submission.

PLATON version of 19/12/2024; check.def file version of 19/12/2024

Datablock shelx - ellipsoid plot

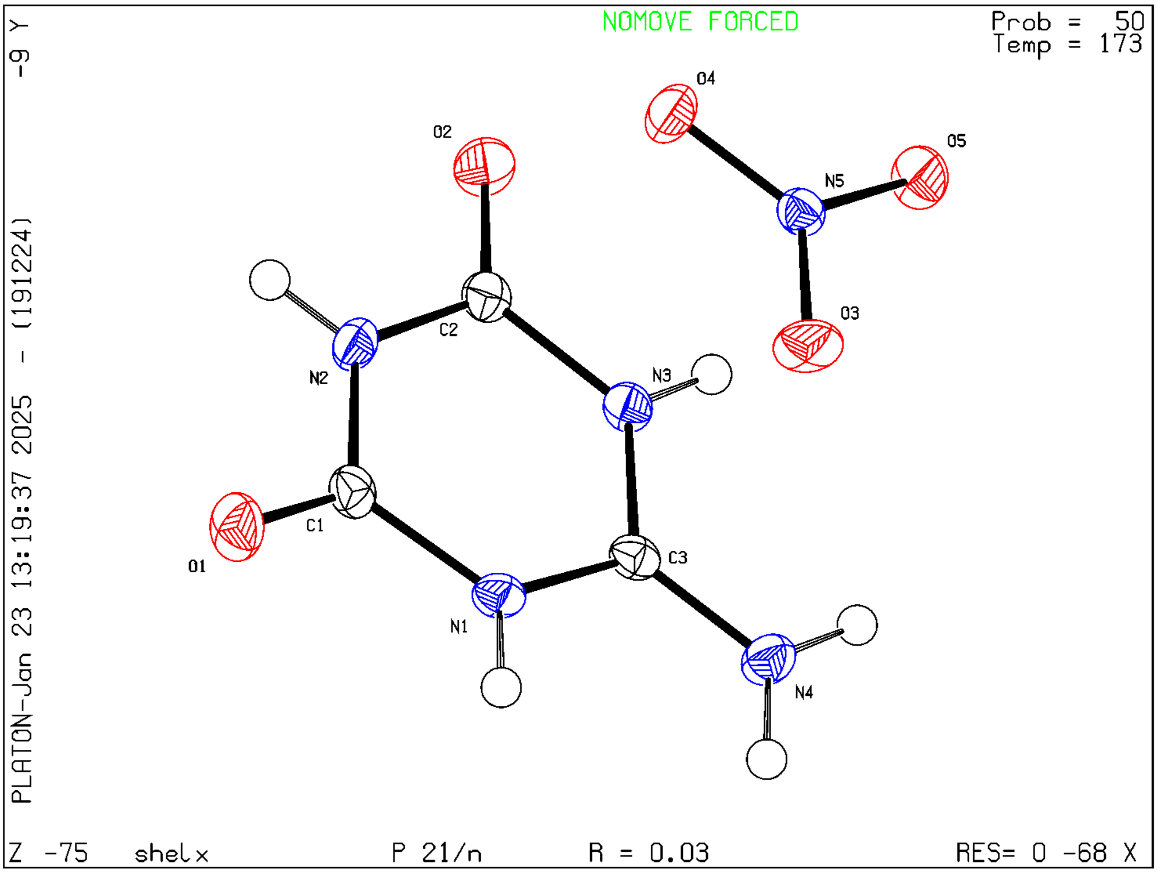

checkCIF/PLATON report

Structure factors have been supplied for datablock(s) shelx

THIS REPORT IS FOR GUIDANCE ONLY. IF USED AS PART OF A REVIEW PROCEDURE FOR PUBLICATION, IT SHOULD NOT REPLACE THE EXPERTISE OF AN EXPERIENCED CRYSTALLOGRAPHIC REFEREE.

No syntax errors found. CIF dictionary Interpreting this report

Datablock: shelx

Bond precision:	O- C = 0.0020 A		Wavelength=0.71073
Cell:	a=7.6704(5)	b=10.2444(6)	c=10.2540(6)
	alpha=90	beta=90.731(2)	gamma=90
Temperature:	173 K		

	Calculated	Reported
Volume	805.68(9)	805.68(8)
Space group	P 21/n	P 21/n
Hall group	-P 2yn	-P 2yn
Moiety formula	C3 H5 N4 O2, Cl O4	?
Sum formula	C3 H5 Cl N4 O6	C3 H5 Cl N4 O6
Mr	228.56	228.56
Dx,g cm-3	1.884	1.884
Z	4	4
Mu (mm-1)	0.491	0.491
F000	464.0	464.0
F000'	464.88	
h,k,lmax	9,13,13	9,13,13
Nref	1853	1851
Tmin,Tmax	0.976,0.976	0.946,1.000
Tmin'	0.976	

Correction method= # Reported T Limits: Tmin=0.946 Tmax=1.000
AbsCorr = MULTI-SCAN

Data completeness= 0.999 Theta(max)= 27.490

R(reflections)= 0.0335(1768) wR2(reflections)= 0.0895(1851)

S = 1.141 Npar= 148

The following ALERTS were generated. Each ALERT has the format

test-name_ALERT_alert-type_alert-level.

Click on the hyperlinks for more details of the test.

Alert level C

```
PLAT242_ALERT_2_C Low    'MainMol' Ueq as Compared to Neighbors of        Cl1 Check
PLAT430_ALERT_2_C Short Inter D...A Contact  O1        ..O3        .       2.89 Ang.
                                         1/2+x,1/2-y,-1/2+z  =      4_665 Check
```

Alert level G

```
PLAT432_ALERT_2_G Short Inter X...Y Contact  O4        ..C3        .       2.91 Ang.
                                                     x,y,z  =      1_555 Check
PLAT432_ALERT_2_G Short Inter X...Y Contact  O6        ..C1        .       2.97 Ang.
                                                     x,y,z  =      1_555 Check
PLAT870_ALERT_4_G ALERTS Related to Twinning Effects Suppressed ..           ! Info
PLAT883_ALERT_1_G Absent Datum for _atom_sites_solution_primary ..     Please Do !
PLAT899_ALERT_4_G SHELXL2018 is Outdated   and Succeeded by SHELXL     2019/3 Note
PLAT910_ALERT_3_G Missing # of FCF Reflection(s) Below Theta(Min).           1 Note
                0  1  1,
PLAT933_ALERT_2_G Number of HKL-OMIT Records in Embedded .res File           1 Note
                2  0  0,
PLAT961_ALERT_5_G Dataset Contains no Negative Intensities .......     Please Check
PLAT967_ALERT_5_G Note: Two-Theta Cutoff Value in Embedded .res ..       55.0 Degree
PLAT969_ALERT_5_G The 'Henn et al.' R-Factor-gap value ..........      5.088 Note
              Predicted wR2: Based on SigI**2  1.76 or SHELX Weight  7.85
```

0 **ALERT level A** = Most likely a serious problem - resolve or explain
0 **ALERT level B** = A potentially serious problem, consider carefully
2 **ALERT level C** = Check. Ensure it is not caused by an omission or oversight
10 **ALERT level G** = General information/check it is not something unexpected

1 ALERT type 1 CIF construction/syntax error, inconsistent or missing data
5 ALERT type 2 Indicator that the structure model may be wrong or deficient
1 ALERT type 3 Indicator that the structure quality may be low
2 ALERT type 4 Improvement, methodology, query or suggestion
3 ALERT type 5 Informative message, check

It is advisable to attempt to resolve as many as possible of the alerts in all categories. Often the minor alerts point to easily fixed oversights, errors and omissions in your CIF or refinement strategy, so attention to these fine details can be worthwhile. In order to resolve some of the more serious problems it may be necessary to carry out additional measurements or structure refinements. However, the purpose of your study may justify the reported deviations and the more serious of these should normally be commented upon in the discussion or experimental section of a paper or in the "special_details" fields of the CIF. checkCIF was carefully designed to identify outliers and unusual parameters, but every test has its limitations and alerts that are not important in a particular case may appear. Conversely, the absence of alerts does not guarantee there are no aspects of the results needing attention. It is up to the individual to critically assess their own results and, if necessary, seek expert advice.

Publication of your CIF in IUCr journals

A basic structural check has been run on your CIF. These basic checks will be run on all CIFs submitted for publication in IUCr journals (*Acta Crystallographica*, *Journal of Applied Crystallography*, *Journal of Synchrotron Radiation*); however, if you intend to submit to *Acta Crystallographica Section C* or *E* or *IUCrData*, you should make sure that full publication checks are run on the final version of your CIF prior to submission.

Publication of your CIF in other journals

Please refer to the *Notes for Authors* of the relevant journal for any special instructions relating to CIF submission.

PLATON version of 19/12/2024; check.def file version of 19/12/2024

Datablock shelx - ellipsoid plot

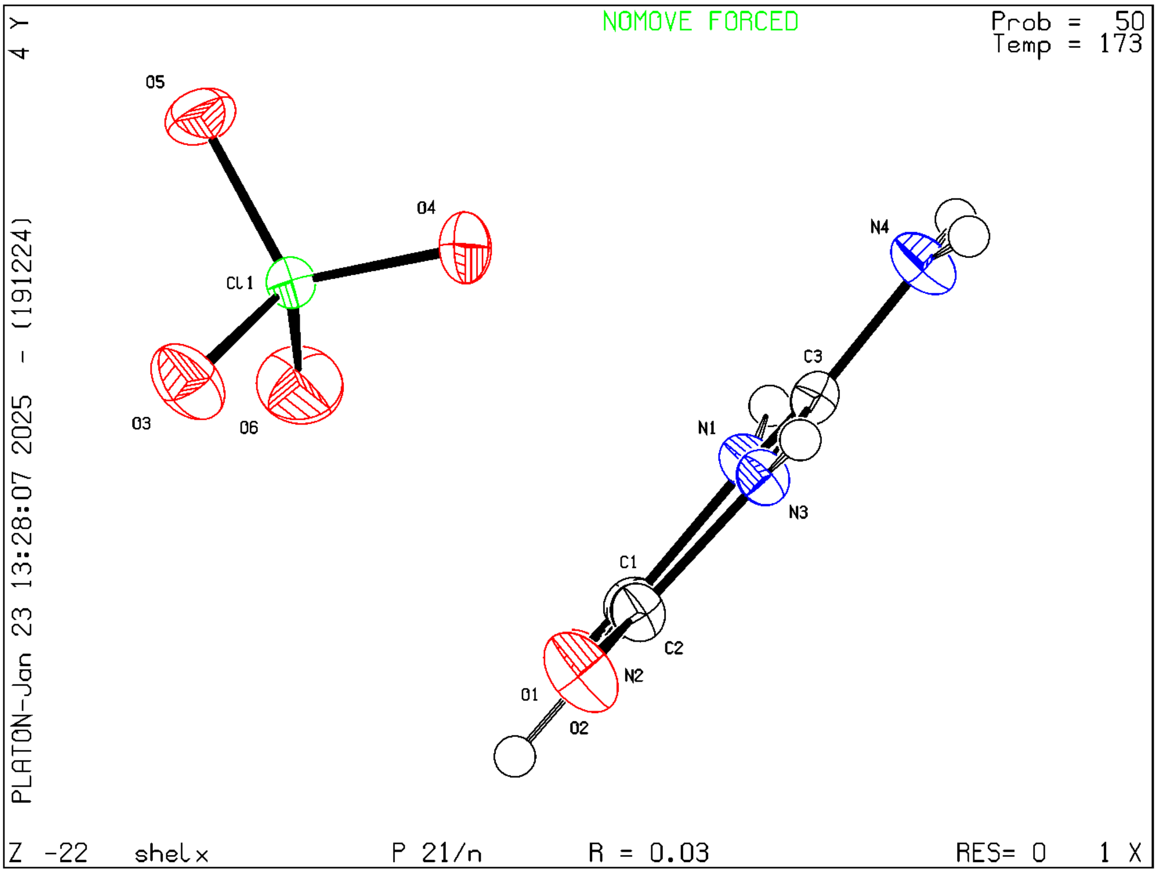

Supplement: Supplementary file 2 — Supporting File 1: chem70637‐sup‐0002‐SuppMat.zip [file CHEM-32-e03587-s001.zip › checkcif_combined.pdf]
